# Natural and Nanomaterial Additives in Biodegradable PLA/PBAT Films: Towards Advanced Packaging Materials

**DOI:** 10.3390/polym18172158

**Published:** 2026-09-03

**Authors:** Mariia Dmitrenko, Ilnur Dzhakashov, Daniel Pasquini, Anna Kuzminova, Anton Mazur, Sabu Thomas, Rongxin Su, Anastasia Penkova

**Affiliations:** 1 St. Petersburg State University, 7/9 Universitetskaya nab., St. Petersburg 199034, Russia; st117543@student.spbu.ru (I.D.); a.kuzminova@spbu.ru (A.K.); a.mazur@spbu.ru (A.M.); 2 Instituto de Química, Universidade Federal de Uberlândia, Campus Santa Mônica, Av. João Naves de Ávila, 2121, Uberlândia 38400-902, Minas Gerais, Brazil; daniel.pasquini@ufu.br; 3 School of Chemical Sciences, Trivandrum Engineering Science & Technology Research Park, Mahatma Gandhi University, Kottayam 686560, Kerala, India; sabuthomas@mgu.ac.in; 4 State Key Laboratory of Chemical Engineering, School of Chemical Engineering and Technology, Tianjin University, Tianjin 300072, China; surx@tju.edu.cn

**Keywords:** polylactic acid, polybutylene adipate terephthalate, curcumin, essential oils, nisin, zinc oxide, biodegradable packaging

## Abstract

This study reports a systematic, single-additive investigation of biodegradable films based on polylactic acid (PLA) and polybutylene adipate terephthalate (PBAT) blends (T2308 and F2332) from Ecovio^®^ for packaging applications by incorporating additives such as nisin, essential oils (tea tree, lemongrass, eucalyptus, clove leaves), curcumin, and zinc oxide nanoparticles. Their effects on structure, morphology, thermal behavior, mechanical properties, barrier performance, and optical properties were evaluated by FTIR, SEM with EDX, TGA, DSC, DMA, XPS, mechanical testing, water vapor permeability, moisture absorption, contact angle measurements and UV/visible transmittance. FTIR confirmed additive incorporation with bonding interactions. SEM revealed matrix-dependent morphologies, with T2308 being denser and more heterogeneous, F2332 being more homogeneous and flexible. DSC/TGA showed curcumin markedly reduces crystallinity and melting enthalpy in T2308 (weaker effects in F2332), while oils generally decrease crystallinity and shift Tg depending on molecular structure. Mechanical testing indicated modulus is highly matrix-dependent: curcumin, nisin, and ZnO decrease stiffness in T2308, whereas F2332 shows smaller or opposite trends. UV shielding increases with curcumin and ZnO, and clove oil improves barrier performance. Biodegradation was assessed only for neat films: weight loss averaged ~4–6% after 49 days, with PET/HDPE remaining largely inert. These results illustrate the importance of matrix–additive interactions in enabling tailored biodegradable packaging materials.

## 1. Introduction

There is widespread concern regarding the production and disposal of plastic materials. Demand for plastic packaging has risen sharply in recent years, driven by population growth and global challenges, including the COVID-19 pandemic [1]. In 2018, plastics accounted for approximately 16% of packaging materials. Non-biodegradable plastic waste, if inadequately managed, can accumulate in ecosystems and cause substantial harm. For example, polyethylene terephthalate (PET), widely used for beverage bottles, is estimated to take about 1179 years to decompose [2]. To reduce the environmental burden associated with this class of materials, alternative options are required. These alternatives must meet several criteria: they should exhibit mechanical, barrier, and optical properties comparable to conventional polymers while providing a significantly shorter biodegradation period.

Polylactic acid (PLA), which is produced from renewable raw materials (such as sugar and corn starch), has recently gained widespread popularity [3]. PLA has a significantly shorter degradation time than petrochemical plastics (with weight loss reaching 77.6% in 177 days under aerobic conditions). PLA plastic production is projected to more than double within 5 years. However, using pure PLA is insufficient for some product-manufacturing applications due to several limitations of the polymer, such as high brittleness and low thermal stability [4]. To overcome these disadvantages, it is proposed to use PLA in a mixture with polybutylene adipate terephthalate (PBAT), which is produced by the polycondensation of 4-butanediol, adipic acid, terephthalic acid [5]. Due to the fact that this polymer is biodegradable, biocompatible, and has excellent mechanical and physicochemical properties, mixing it with PLA allows not only to overcome the problems associated with a pure polymer, but also to improve the mechanical, rheological, and barrier properties of the final material [5,6,7].

Along with the need to obtain packaging material from PLA/PBAT, it is necessary to achieve properties that can improve the characteristics of the film being developed. This can be achieved by introducing additional substances at the synthesis stage, which can enable the development of so-called smart packaging. These films can be used to monitor the condition of the product (for example, by changing the color of the package depending on the pH of the medium), while active packaging can directly affects the packaged product (e.g., through the expression of antioxidant properties) [8,9].

Curcumin (chemical formula: C_21_H_20_O_6_) is the main compound (polyphenol) found in the root of turmeric (Curcuma longa). Curcumin gives turmeric its characteristic bright yellow color [10]. Due to its structure and origin, this substance is virtually insoluble in water and possesses antioxidant, anti-inflammatory, and antimicrobial properties making it a promising additive for food packaging. Curcumin has found widespread use in various areas of human life: in the food industry as a natural colorant known as E100, in medicine, and in cosmetics [10,11]. The addition of curcumin to food packaging has been reported to improve its mechanical, barrier, and optical properties. Another important property of curcumin is its ability to change color depending on the pH of the environment, which can be used to monitor product quality. The study confirmed a color change from yellow to orange in curcumin-infused films during 7 days of chicken storage, confirming the compound’s potential use as a pH-sensitive agent [12].

Nisin is a unique natural antimicrobial peptide (bacteriocin) that occupies a special place in food technology, as it is the only bacteriocin officially approved in most countries as a food preservative (food additive E234). Its use in food packaging is one of the most promising areas for extending the shelf life of products. Nisin is highly effective against Gram-positive bacteria and helps extend the shelf life of various animal-based foods [13]. The availability and properties of nisin make it a promising in the development of packaging materials.

The growth of various types of undesirable fungi and bacteria can be inhibited by adding essential oils. Natural essential oils contain various components capable of selectively and/or specifically preventing bacterial growth, thereby reducing the risk of ill foodborne illness associated with consuming spoiled food. However, some oils, such as clove oil, because of their eugenol and β-caryophyllene content, can exhibit broad-spectrum antimicrobial activity against various bacteria and fungi. Furthermore, essential oils can impart a light, pleasant aroma to food packaging, helping to suppress unpleasant odors in the event of food spoilage, and provide hydrophobic properties, which can help extend shelf life by reducing exposure to moisture.

It is also worth mentioning the trend toward using zinc oxide, which protects food from ultraviolet radiation and enhances the mechanical and barrier properties of food packaging materials [14,15]. Despite its inorganic nature, zinc oxide is considered safe and exhibits negligible migration into the packaged product.

Although numerous studies have reported on PLA/PBAT packaging, a distinct research gap remains in systematically isolating the effects of individual additives within Ecovio^®^-derived blends and distinguishing the novelty of the present work from prior studies. In this study, this gap was addressed by adopting a single-additive approach to incorporate nisin, selected essential oils (tea tree, lemongrass, eucalyptus, clove leaves), curcumin, and zinc oxide nanoparticles into PLA/PBAT films, enabling an independent assessment of each additive’s impact on structure, morphology, crystallinity, thermal behavior, barrier properties, hydrophobicity, and UV shielding. By optimizing additive concentrations and elucidating their interactions within the polymer matrix, it is possible to tailor the films to meet the demanding requirements of packaging applications while maintaining biodegradability and environmental safety. A unified set of characterization techniques (FTIR, SEM, TGA, DSC, DMA, XPS, mechanical testing, etc.) is employed to elucidate interaction mechanisms and stability, thereby guiding the optimization of additive loadings. The novelty lies in the systematic, isolated evaluation of each additive’s influence and in providing a strategic framework for tailoring Ecovio^®^-based films to deliver enhanced functionalities that can support the development of advanced, environmentally friendly packaging materials. This strategy aligns with global efforts to reduce plastic waste.

## 2. Materials and Methods

### 2.1. Materials

Polylactic acid (PLA) and polybutylene adipate terephthalate (PBAT) blends, marketed under two different commercial names—T2308 and F2332—from Ecovio^®^ [16], were used as the film matrix. Among the various commercial grades of Ecovio^®^ (BASF SE), Ecovio^®^ T2308 and Ecovio^®^ F2332 were specifically selected as the base polymer matrices due to their complementary PLA/PBAT blend ratios, processing characteristics, and high industrial relevance for food packaging. Both grades are certified as fully compostable and compliant with international food-contact regulations. Ecovio^®^ T2308 exhibits higher structural rigidity and tensile strength due to a higher PLA content (80–90%), making it suitable for applications requiring dimensional stability. Conversely, Ecovio^®^ F2332 is specifically formulated for blown film extrusion, offering superior ductility, tear resistance, and flexibility owing to a higher PBAT fraction (30–50%). Employing these two distinct grades allows for a systematic comparative investigation of how matrix flexibility, polymer chain mobility, and PLA/PBAT phase morphology influence the entrapment, spatial dispersion, and release kinetics of diverse additives (nisin, curcumin, ZnO nanoparticles, and essential oils), thereby providing a rational approach to tailoring packaging films for specific food preservation needs. The characteristics of T2308 and F2332 are detailed in Table 1.

Additives included nisin (E234, STOING, Foodchem International Corporation, Shanghai, China), essential oils from tea tree (TT), lemongrass (LG), Eucalyptus Globulus (EL), and clove leaves (CL), all purchased from ViaAroma (Uberlândia, Brazil). Additionally, curcumin (turmeric from Pirata, Vilma, Brazil) and zinc oxide nanoparticles (ZnO, Type I, Sisco Research Laboratories Pvt. Ltd. (SRL), Mumbai, India) were used without any prior treatment. Chloroform (CHCl_3_, 99.1 wt.%, Vekton, St. Petersburg, Russia) was used as a solvent.

### 2.2. Film Preparation

The preparation method utilizing T2308 and F2332 (with various PLA/PBAT combinations) was conducted as follows: an appropriate amount of granules (4 g) was weighed and dissolved in 50 mL of chloroform under vigorous stirring for 1.5 h at room temperature until the solution was fully homogenized. The polymer-to-solvent ratio was determined based on a three-stage optimization process, which included: (1) selecting the solvent—chloroform was preferred over acetone due to its superior solubility and its ability to form high-quality film structures; (2) optimizing the concentration to achieve homogeneous dispersion while minimizing solvent usage; and (3) adjusting the time and temperature to ensure complete dissolution and process stability.

Additional components were incorporated by adding them to the PLA/PBAT mixture after 1.5 h of stirring. Subsequently, the solution was stirred for an additional 30 min to ensure proper dispersion and homogenization of the additives. To determine the optimal concentrations of the additives, the following ranges with respect to the polymer weight were tested: curcumin (5–15 wt.%), ZnO nanoparticles (5–10 wt.%), and nisin (10–30 wt.%). The choice of the most effective essential oil (fixed at 5 wt.%) was based on experimental data, including water vapor permeability, moisture absorption, and other relevant parameters.

**Figure 1:**
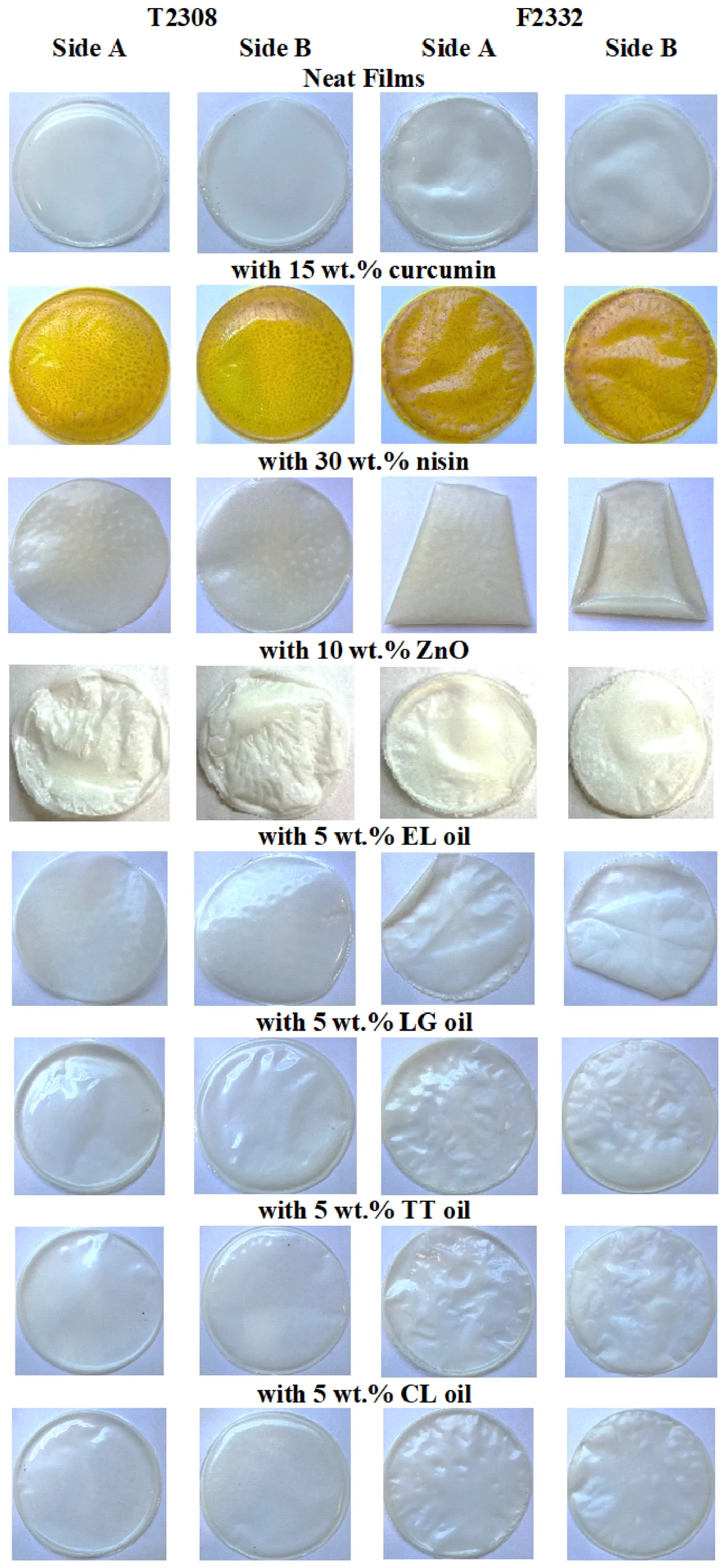
Photographs of F2332 and T2308 films, both without and with the maximum concentration of various additives.

In the final stage, the prepared solution was poured into glass Petri dishes and allowed to dry at room temperature. The film thickness was measured with a digital external micrometer (Schut Geometrical Metrology, Groningen, The Netherlands). To ensure precision, at least seven random measurements were performed at different locations on each sample, and the mean value was calculated. The average thickness was determined to be 234 ± 41 µm. Figure 1 illustrates photographs of the obtained films, both without and with various additives.

### 2.3. Film Characterization

The molecular structure of the synthesized substances and the derived films was examined using Fourier-transform infrared (FTIR) spectroscopy. Spectroscopic analyses were conducted at ambient temperature utilizing a TENSOR 27 instrument (BRUKER, St. Petersburg, Russia), employing the potassium bromide (KBr) pellet method. The chemical composition of the films after biodegradation was characterized through solid-state ^13^C nuclear magnetic resonance (NMR) spectroscopy. Spectra were obtained using a Bruker Avance III 400 WB spectrometer (Billericay, MA, USA, 9.4 T) equipped with a 4 mm CP/MAS probe. The measurements were conducted at a magic angle spinning (MAS) rate of 10 kHz, with a ^13^C resonance frequency of 100.64 MHz. Tetramethylsilane (TMS) served as the external reference standard. The surface and cross-sectional morphologies of the films were analyzed via scanning electron microscopy (SEM). Specifically, the Zeiss AURIGA Laser SEM (Carl Zeiss SMT, Oberkochen, Germany) was used for imaging. Before imaging, samples were cryo-fractured in liquid nitrogen to obtain clean cross-section and then sputter-coated with a thin gold layer to improve electrical conductivity and image quality.

The mechanical properties of the films were assessed through uniaxial tensile testing using a Shimadzu AG-50kNXD Autograph universal testing machine (Kyoto, Japan). Tests were performed according to the standards outlined in ASTM D638-14 [17] and ISO 527-2:2012 [18], at a crosshead speed of 100 mm/min. Samples were cut to dimensions of 5 mm in width and 70 mm in length, with a 30 mm gauge length for tensile testing. Dynamic mechanical analysis (DMA) was conducted to determine the Young’s modulus of the samples. Tests were performed using a Q800 DMA instrument (TA Instruments, New Castle, DE, USA) in the uniaxial tension mode. Stress−strain measurements were recorded from 0 to 18 N with a ramp rate of 3 N/min at 25 °C. Thermogravimetric analysis (TGA) was carried out to evaluate the thermal stability of the films under inert conditions. This analysis was performed using a TG 209 F1 Libra thermogravimetric analyzer (Netzsch, Selb, Germany) under an argon atmosphere (50 mL/min) with a heating rate of 10 °C/min. Differential scanning calorimetry (DSC) analyses of the films were performed on a TA Instruments Q-20 in aluminum pans. A heating ramp was conducted from 25 to 180 °C, followed by a second heating ramp from −50 to 250 °C under a nitrogen flow of 50 mL/min at a heating rate of 10 °C/min. The second heat was presented in figures from 3 °C to avoid the sharp beginning of the curves.

Surface hydrophilicity was assessed by determining the static water contact angle using the sessile drop method with an LK-1 goniometer (NPC “Open Science”, Krasnogorsk, Russia). A water droplet was carefully placed on the film surface, and images were recorded at 3–4 s intervals across at least nine different locations per sample. The captured images were analyzed with DropShape software (version 1, Laboratory of Mathematical Methods of Image Processing, Lomonosov Moscow State University, Moscow, Russia), and the mean contact angle was calculated by averaging the seven most reproducible measurements. Moisture absorption (MA) was assessed based on the procedure: samples, dried to a stable weight (w_i_) at 60 °C, were then placed in a desiccator saturated with NaCl solution, maintaining a relative humidity (RH) of 75%. After 24 h, the samples were reweighed (w_f_), and the moisture absorption was calculated using Equation (1):

(1) $$MA=\frac{\left(w_{f}-w_{i}\right)}{w_{f}}\cdot 100\%,$$

Water vapor permeability (WVP) was measured gravimetrically based on a modified protocol of ASTM E96-95 [19,20]. The samples were sealed within a test assembly that enclosed a chamber containing silica gel (maintaining 0% RH) and then positioned inside a desiccator saturated with NaCl solution to generate approximately 75% RH. The samples were periodically weighed to monitor water uptake, enabling the calculation of the vapor flux. The WVP was then determined using Equation (2):

(2) $$WVP=\frac{∆m\cdot l}{t\cdot A\cdot p\cdot ∆RH},$$

where Δm refers to the mass increase (g), l denotes the mean thickness of the film, t signifies the duration of the experiment (s), A represents the effective area for permeation (m^2^), p is the saturation vapor pressure at 25 °C (3.169 kPa), and ΔRH is the humidity gradient, assumed to be 0.75.

Before conducting the WVP, MA, and contact angle measurements, the samples were dried at 60 °C until a constant mass was achieved.

The optical characteristics, including light transmittance (T), and overall transparency, were measured using a PE-5400UV spectrophotometer [19]. Rectangular film samples measuring 10 × 45 mm were placed inside a glass cuvette, and spectra were recorded over the wavelength range of 350 to 750 nm, using an empty cuvette (air) as the baseline. The percentage of transmittance was calculated using Equation (3):

(3) $$T=10^{-D}\cdot 100\%,$$

### 2.4. Film Biodegradation

The biodegradation performance of the T2308 and F2332 films was evaluated in comparison with reference materials—polyethylene terephthalate (PET), high-density polyethylene (HDPE). The composting experiment was conducted following the ISO 20200 standard [21,22]. Film specimens, with an initial dry weight (w_i_), were placed in perforated containers to facilitate microbial activity, moisture ingress, and subsequent recovery. These containers were incorporated into a synthetic waste matrix composed of 45% of a specific mixture comprising 10% compost, 30% rabbit feed, 10% starch, 5% sugar, 4% oil, 1% urea, and 40% sawdust—and mixed with 55% water. The biodegradation process was maintained under aerobic conditions at 28 °C. At predetermined intervals, samples were retrieved, vacuum-dried at 40 °C for 2 h, and weighed to determine the final mass (w_f_). Each data point represents the mean of at least three replicates. The extent of degradation, expressed as weight loss (WL), was calculated using Equation (4):

(4) $$WL=\frac{\left(w_{i}-w_{f}\right)}{w_{i}}\cdot 100\%,$$

### 2.5. Statistical Analysis

Data were expressed as mean values ± standard deviations, derived from a minimum of three replicate measurements. The assumption of a normal distribution of the data was verified, and any outliers were omitted from subsequent analysis. The final evaluation incorporated the measured values, their associated random uncertainties, the cumulative systematic errors that were not excluded, and the overall uncertainties of the obtained results.

Experimental data were subjected to analysis of variance (ANOVA) to evaluate the existence of significant differences among treatments (presented in the Supplementary Materials). Subsequently, means were compared using Tukey’s test, adopting a significance level of 5% (α = 0.05). All statistical analyses were performed using OriginPro 8.5 software (OriginLab Corporation, Northampton, MA, USA). Comparisons were made only within each group sharing the same polymer and additive.

## 3. Results and Discussion

### 3.1. Structure and Morphology

**Figure 2:**
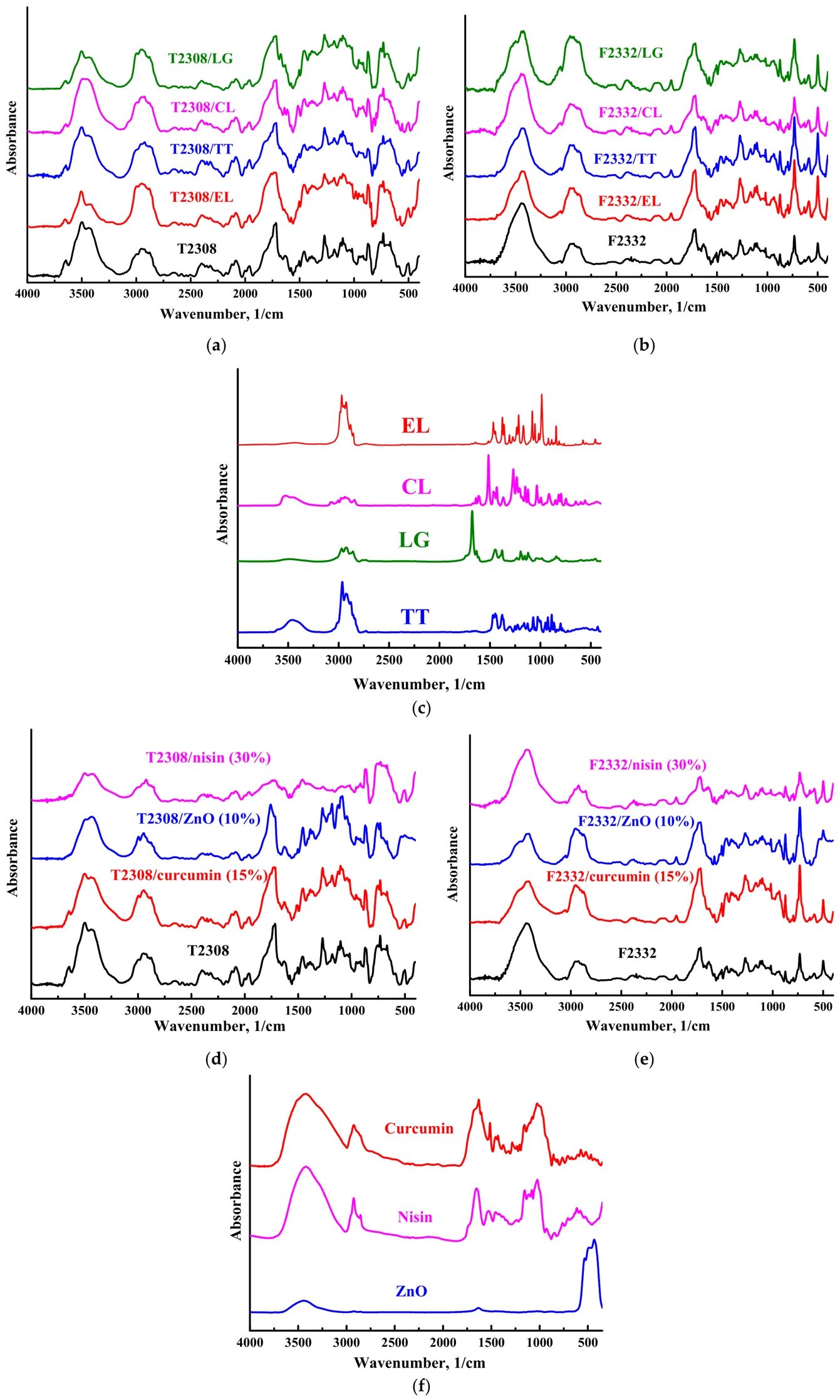
FTIR spectra of (**a**,**b**) T2308 and F2332 films with oils and (**c**) FTIR spectra of pristine oils, (**d**,**e**) T2308 and F2332 films with other additives (nisin, curcumin, ZnO) and (**f**) FTIR spectra of these pristine additives.

The molecular structures of the individual T2308 and F2332 films, along with various additives (oils, nisin, curcumin, and ZnO), as well as the individual components, were analyzed using FTIR spectroscopy (Figure 2).

The FTIR spectra of the T2308 and F2332 films reveal characteristic absorption bands corresponding to both PLA and PBAT components. For PBAT, prominent bands are observed in the 1000–1210 cm^−1^ range, attributable to aromatic benzene ring vibrations, along with a band at 1271 cm^−1^ stemming from the C–O stretching vibrations in ester groups, and a band at 1181 cm^−1^ associated with symmetric C–O vibrations in adipate and terephthalate moieties. Both polymers exhibit an absorption band at 1717 cm^−1^, which corresponds to C=O stretching vibrations [23]. The main bands characteristic of PLA include bands at 1455 cm^−1^, attributed to C–H deformation vibrations in methyl groups (CH_3_), and at 1386 cm^−1^, corresponding to symmetric C–H deformation. Additional bands at 1180 and 872 cm^−1^ are assigned respectively to C–O–C stretching and C–COO vibrations [24]. Specifically for pure PLA, a strong C=O stretching vibration appears at 1731 cm^−1^, with asymmetric and symmetric –CH_3_ stretching modes observed at approximately 2995 and 2949 cm^−1^, respectively, along with C–O stretching bands around 1081 cm^−1^. The spectrum of pristine PLA also features vibrational bands at 1456 cm^−1^ (asymmetric –CH_3_), 1363 cm^−1^ (symmetric –CH_3_), and 1104–874 cm^−1^ assigned to C–O–C and C–COO groups, respectively [24,25]. Additionally, a broad band around 3450 cm^−1^ indicates water absorption, likely due to moisture uptake by the film.

The FTIR spectrum of EL oil is characterized by a distinct band at 987 cm^−1^, attributed to a CH_2_ wagging vibration. Further characteristic bands, though less intense, correspond to its main component, 1,8-cineole. These include the symmetric (1080 cm^−1^) and asymmetric (1215 cm^−1^) C–O–C stretching vibrations, as well as the symmetric CH_3_ deformation modes observed at 1376 cm^−1^ [26]. The spectrum also confirmed the presence of characteristic functional groups in the oil. Bands at 2969 and 2882 cm^−1^ were assigned to symmetric and asymmetric CH_3_ stretching, respectively, while the 2926 cm^−1^ band corresponded to asymmetric CH_2_ stretching. The absorption at 1643 cm^−1^ indicated C=C stretching vibrations of olefinic components. Additionally, a strong band at 1466 cm^−1^ was attributed to the in-plane symmetric C–H bending vibration. Notably, the absence of absorptions in the 3200–3600 cm^−1^ region, characteristic of O–H groups, indicates a low content of compounds such as citronellol, isopulegol, and neo-isopulegol in the isolated oil [26].

The FTIR spectrum of TT oil shows an absorption band at 3460 cm^−1^, which is assigned to the O–H stretching vibration. Strong bands at 2963, 2926, and 2878 cm^−1^ correspond to C–H stretching vibrations, specifically from –CH_2_– groups, as well as asymmetric –CH(CH_3_) and –CH(CH_2_)– stretching vibrations, including symmetric and asymmetric modes. Weak bands in the range of 1690 to 1580 cm^−1^ are due to the stretching vibration of the alkene functional group (C=C) [27].

The FTIR spectrum of CL oil exhibited characteristic absorption bands of its main component, eugenol: a band at around 3524 cm^−1^ attributed to O–H stretching; bands at 3077, 1638, and 914 cm^−1^, assigned to =C–H stretching, C=C stretching, and –CH_2_ bending vibrations, respectively, indicating the presence of a vinyl group; and bands at 1613, 1514, and 1432 cm^−1^, arising from aromatic ring vibrations [28].

The FTIR spectrum of LG oil revealed absorption bands at 1738 cm^−1^, assigned to C=C stretching vibrations, confirming the presence of a conjugated double bond system (C=C–CHO) in citral, which is common in acyclic monoterpenes. The peak at 1632 cm^−1^ indicated the stretching of the C=O bond of the aldehyde group. At the 1379 cm^−1^ peak, bending of the -CH_2_ group was observed [29]. Furthermore, the peak at 3400 cm^−1^, assigned to O–H stretching vibrations, was relatively narrow and not the most intense in the spectrum, indicating a low water content in the LG oil.

The introduction of various essential oils into the T2308 and F2332 matrices induced minor spectral modifications. The emergence or increased intensity of bands associated with alkyl and olefinic groups (e.g., C–H and C=C vibrations) confirms the presence of essential oil constituents within the film structure. Slight intensity variations in characteristic C=O and C–O stretching bands suggest physical compatibility between the oils and the polymer phases. Residual moisture remains evident as broad absorption bands around 3450 cm^−1^. Overall, these spectral modifications confirm the successful incorporation of the essential oils into the PLA/PBAT matrices without causing chemical degradation of the polymer backbone.

The spectrum of commercial ZnO nanoparticle has a characteristic high-intensity band at around 435 cm^−1^, which is attributed to the Zn-O vibrational mode. The low-intensity bands in the region of 3442 cm^−1^ and 1632 cm^−1^ are related to the valence and strain stretching of the O-H bond present in water [30]. The nisin spectrum is characterized by the presence of a wide band in the range of 3500–3300 cm^−1^, which corresponds to the stretching of O-H carbonyl groups and the stretching of N-H in amide and amine groups. The adjacent band at 2850–2950 cm^−1^ is typical of the stretching vibrations of alkyl groups [31]. The characteristic band at 1650 cm^−1^ refers to the stretching of C=O amide fragments (amide-I), and at 1530 cm^−1^ the bending of N-H (amide-II) [32]. The main characteristics bands of pure curcumin were observed at around 3500–3000 cm^−1^ due to the phenolic and alcoholic O–H stretching vibration, with sharp absorption bands at 1605 cm^−1^ due the stretching vibrations of benzene ring, at 1514 cm^−1^ due to the C=O and C=C vibration, at 1431 cm^−1^ due to olefinic C–H bending vibration, at 1284 cm^−1^ due to aromatic C–O stretching vibration and at 1026/856 cm^−1^ due to C–O–C stretching vibrations of the curcumin [33].

Comparative analysis of the FTIR spectra of films containing nisin, ZnO, and curcumin confirms the presence of these agents within the polymer matrices. For ZnO-containing films, the characteristic Zn–O vibrational band around 435 cm^−1^ remains present, alongside water-related absorptions near 3442 and 1632 cm^−1^, as also reported in previous studies [34]. For nisin-modified films, characteristic peptide absorptions (amide-I at ~1650 cm^−1^ and amide-II at ~1530 cm^−1^) coexist with N–H/O–H stretching bands (3500–3300 cm^−1^). In curcumin-containing films, characteristic bands corresponding to benzene ring vibrations (~1605 cm^−1^) and aromatic C–O stretching (~1284 cm^−1^) confirm the retention of the polyphenol structure [35].

In summary, the overlay of FTIR spectra reveals additive-specific absorption bands within the T2308 and F2332 polymer materials, confirming successful incorporation while preserving the main absorption bands of the PLA/PBAT backbone. The observed spectral modifications primarily involve the emergence of additive-specific functional group bands and minor intensity variations. Given that peak shifts are minimal and spectral deconvolution was not performed, these FTIR data are interpreted as evidence of physical incorporation and compatibility, rather than definitive evidence of chemical bonding or structural modification. The effects of these additives on the physical, mechanical, barrier, and functional properties of the materials are evaluated independently and characterized through dedicated measurements in subsequent sections.

**Figure 3:**
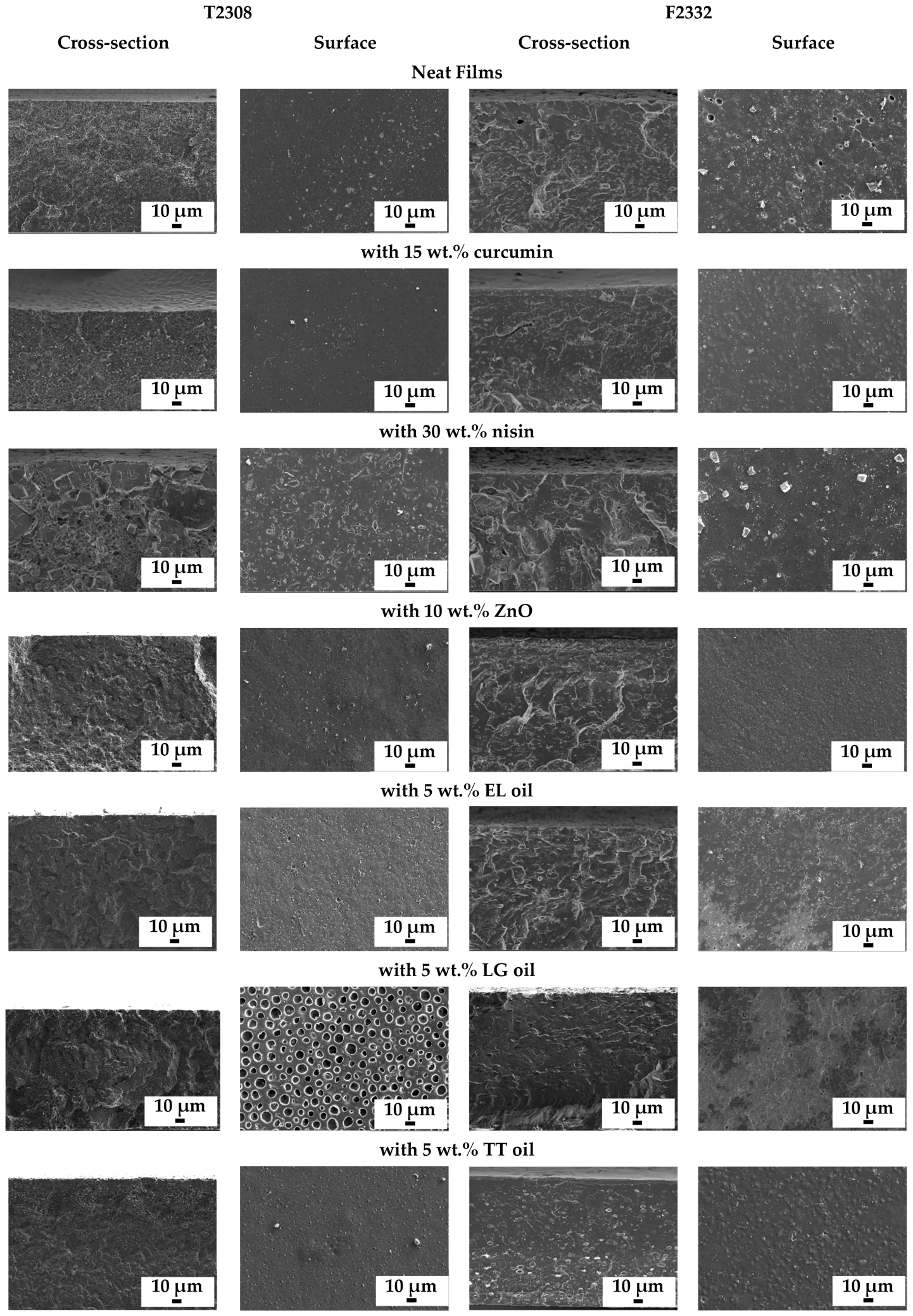

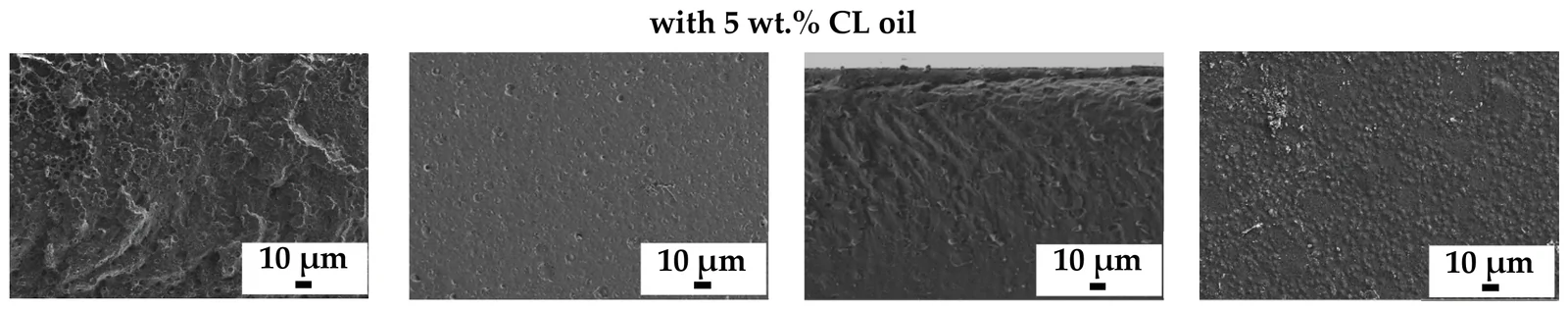
Cross-sectional and surface SEM micrographs of F2332 and T2308 films, acquired at 1.00 k× magnification, both without and with the maximum concentration of various additives.

The surface and cross-sectional morphology of the films was examined using SEM. The resulting SEM images are shown in Figure 3.

The cross-sectional and surface SEM micrographs reveal distinct morphological features in both the neat and additive-modified films. The micrographs consistently show that the base material of T2308 exhibits a relatively rough cross-sectional morphology with microporosity and interconnected structures, along with a smooth surface texture, indicative of a semi-porous matrix. In contrast, the F2332 film presents a rougher cross-section and surface with fewer irregular features.

The addition of various additives induces notable alterations in the structural characteristics. The general cross-sectional trend is as follows: the introduction of any additive into the T2308 film disrupts the structure of the sample, creating new layers of “scales”. In contrast, the structure of the samples from F2332 shows the opposite trend, with the film cross-section becoming more homogeneous and less layered. This is due to the greater tendency of the components to mix with F2332 because of its higher content of PBAT, which has the highly flexible and less densely packed molecular structure compared to PLA [36]. When the film dries, PBAT provides additional free volume for accommodating the additives T2308, due to its denser structure, does not allow sufficient mixing of the PLA/PBAT matrix with curcumin, nisin, and some oils. These morphological observations are quantitatively reflected in the mechanical performance of the composite films. In the case of F2332-based films, the transition toward a more homogeneous, less layered cross-sectional structure restricts local stress localization, which quantitatively correlates with an increase in the Young’s modulus compared to the neat F2332 matrix. Conversely, for the denser T2308 matrix, the insufficient mixing and the formation of “scale-like” layered interfaces and microvoids upon additive incorporation act as mechanical defects.

When 15 wt.% of curcumin is incorporated, the micrographs indicate a slight densification of the matrices [37], with the disappearance of microvoids and a more compact structure, as observed in both T2308 and F2332 formulations. The SEM micrographs with 30 wt.% nisin display an increase in surface heterogeneity, with the appearance of particulate domains and microporosities, particularly in T2308, implying potential phase separation or particle aggregation within the polymer matrices. The inclusion of 10 wt.% ZnO nanoparticles results in a microstructure characterized by dispersed granular particles embedded within the matrices [38]. These particles are clearly discernible in the F2332 formulation, reflecting effective dispersion, though some clustering is still apparent.

The oils—EL, LG, and TT—at 5 wt.% each, induce varying morphological effects. EL oil appears to promote microvoid formation and increased porosity especially in T2308, likely due to its plasticizing effect, which enhances phase separation and void formation [39]. LG oil leads to the development of honeycomb-like structures on the surface [40], most prominently in T2308, with well-defined micropores indicative of a foam-like surface morphology. TT oil maintains a relatively smoother surface with fewer microstructural defects, which indicates better compatibility or dispersion within the blend matrices. CL oil at 5 wt.% produces microvoids and globular inclusions, often associated with the presence of phase-separated regions from the extract, contributing to surface roughness, but to a lesser extent than the use of other oils. Among all the oils evaluated, CL oil exhibited the most balanced morphological characteristics, which may be attributed to eugenol, its main component, and its aromatic structure.

Overall, the SEM micrographs reveal that the T2308 formulation generally exhibits a rougher, more heterogeneous internal structure and surface morphology, both with and without additives, compared to the smoother and more uniform surface features observed in F2332. Additives such as curcumin and nisin increase plastic deformations and heterogeneity, whereas certain oils like LG promote cellular or foam-like structures, and others such as TT and CL oils maintain a more uniform morphology. Zinc oxide nanoparticles are effectively dispersed but contribute to granular surface features. These morphological variations imply that formulation differences and additive interactions significantly influence the internal and surface microstructure, which can impact the physical, barrier, and functional properties of these biodegradable blends.

The homogeneous dispersion and compatibility of the additives within the polymer matrix were also confirmed by X-ray photoelectron spectroscopy (XPS) and field-emission scanning electron microscopy (FE-SEM) coupled with an energy-dispersive X-ray (EDX) detector (presented in the Supplementary materials, Table S1–S4). XPS indicates that the polymer backbone predominates (C1s 72.7–85.0 at%, O1s 12.4–25.3 at%), with trace amounts of N, Cl, and S. Nisin-containing films show surface N1s contents of up to 2.51 at% and S2p ≈ 0.65 at%, while ZnO is evidenced by Zn2p at 0.07–0.10 at%, consistent with nanoparticles being largely encapsulated in the bulk. Lipophilic essential oils, especially TT oil with C1s ≈ 85.0 at%, increase the surface carbon content due to hydrocarbon-rich terpenoids; curcumin maintains the C–O balance (P2p ≈ 0.55 at% in F2332+Curcumin). SEM-EDX shows additive-free films are dense and uniform; nisin induces discrete micron-scale aggregates; ZnO particles are embedded with some aggregation; curcumin and essential oils remain largely uniform, indicating molecular/nano-scale dispersion or seamless integration. Overall, these results provide quantitative evidence for the homogeneous dispersion and compatibility of all additives within both film matrices.

### 3.2. Mechanical and Thermal Properties

**Figure 4:**
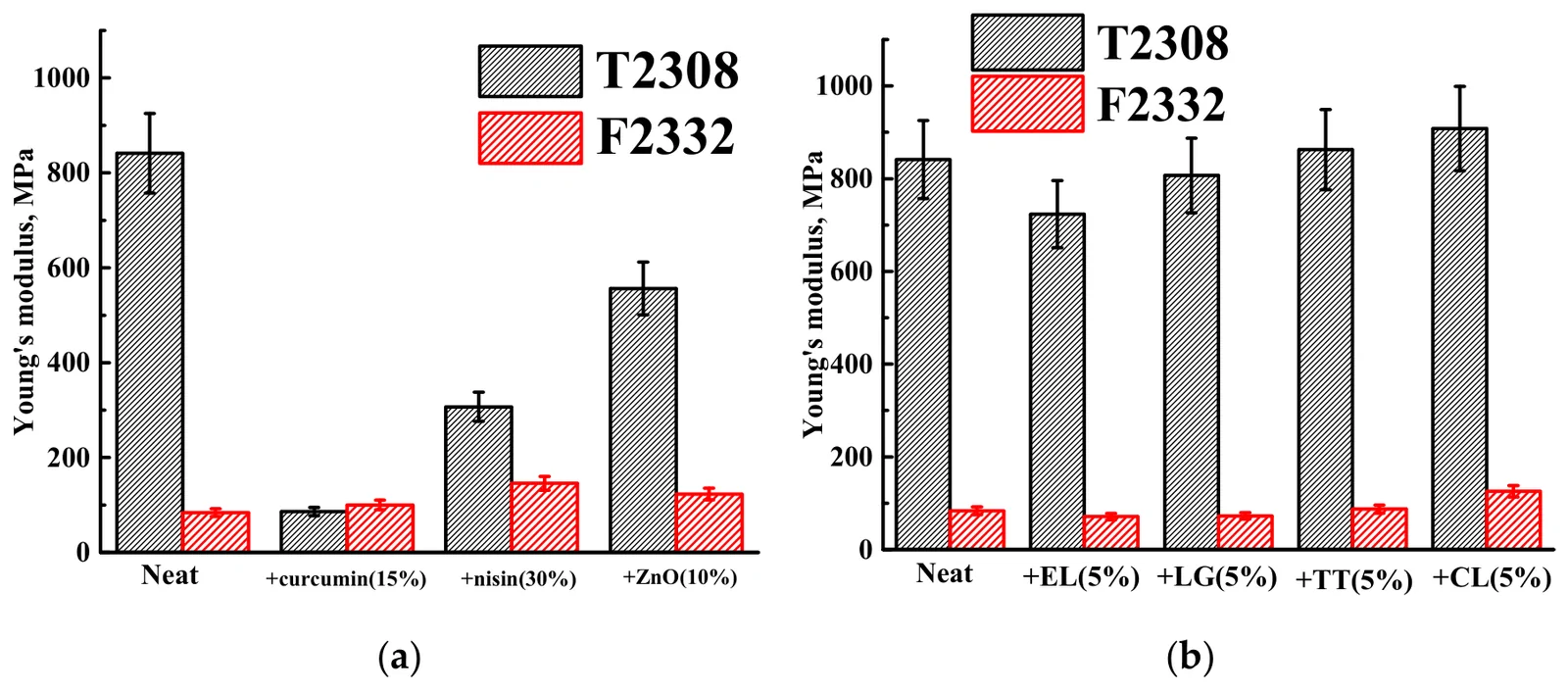
Young’s modulus of the T2308 and F2332 films, both without and with (**a**) the maximum concentration of curcumin, nisin, ZnO and (**b**) oils.

Achieving superior mechanical properties is crucial for guaranteeing the long-term stability and performance of a high-quality packaging material. The summarized information on the Young’s modulus of the films is presented in Figure 4. Maximum elongation and tensile strength values of the films are presented in Table S5 of the Supplementary Materials.

The influence of various additives on the mechanical properties—specifically Young’s modulus—of the T2308 and F2332 films reveals distinct trends depending on both the matrix composition and the nature of the additive. The mechanical data demonstrate clear differences not only between films with and without additives, but also between the T2308 and F2332 formulations themselves. For the T2308 film, the incorporation of curcumin (15%), nisin (30%), and ZnO nanoparticles (10%) each leads to a decrease in Young’s modulus compared to the unmodified sample, indicating a reduction in material stiffness. This decline in modulus indicates that these additives act as plasticizers or disrupt the polymer network, making the material less rigid. F2332 samples show a different response to these additives: in all tested compositions, Young’s modulus remains at approximately the same level, with a slight increase compared to the control sample. However, the overall values for Young’s modulus in the F2332 matrix are lower than those observed for T2308, both with and without additives.

Evaluation of essential oils and comparative analysis of both matrices further highlighted the specificity of the additive effect. EL and LG oils led to reductions compared to the control, while CL and TT oils actually increased the modulus, most notably CL, whose films reached stiffness comparable to or exceeding the control. Certain essential oils can interact synergistically with the matrices, possibly through specific intermolecular interactions or partial cross-linking. Among all the oils evaluated, CL oil provided the most balanced mechanical and morphological performance in T2308 samples and one of the best performances in F2332 samples. This observation is attributed to the influence of the main component of each oil. Table 2 presents the structural characteristics and main components of each oil.

These compounds not only impart a mild, pleasant aroma to the films but can also influence their mechanical properties by providing additional reinforcement. Eugenol, a principal component of CL oil, contains an aromatic (benzene) ring that confers chemical and structural stability [43]. When such aromatic rings are embedded in accessible regions of the films, they can act as rigidifying units that reinforce and stiffen the polymer network, as also reported in a previous study [44]. By contrast, the other oil components lack aromatic systems and contain carbon atoms predominantly in the sp^3^ hybridization state; consequently, molecules such as terpinen-4-ol, eucalyptol and citral exhibit greater conformational flexibility due to free rotation about σ-bonds and act as plasticizers, increasing the free volume between polymer chains [45].

In summary, both the magnitude and direction of the effects of these additives on Young’s modulus were strongly matrix-dependent. T2308-based matrices possessed a substantially higher modulus overall and were more susceptible to reductions in modulus by curcumin, while F2332 matrices started from a lower modulus baseline and in some cases—particularly with nisin—demonstrated an enhancement in modulus. Essential oils had a more variable but generally less drastic effect, with TT and CL oils emerging as potential stiffening agents for both matrices.

**Figure 5:**
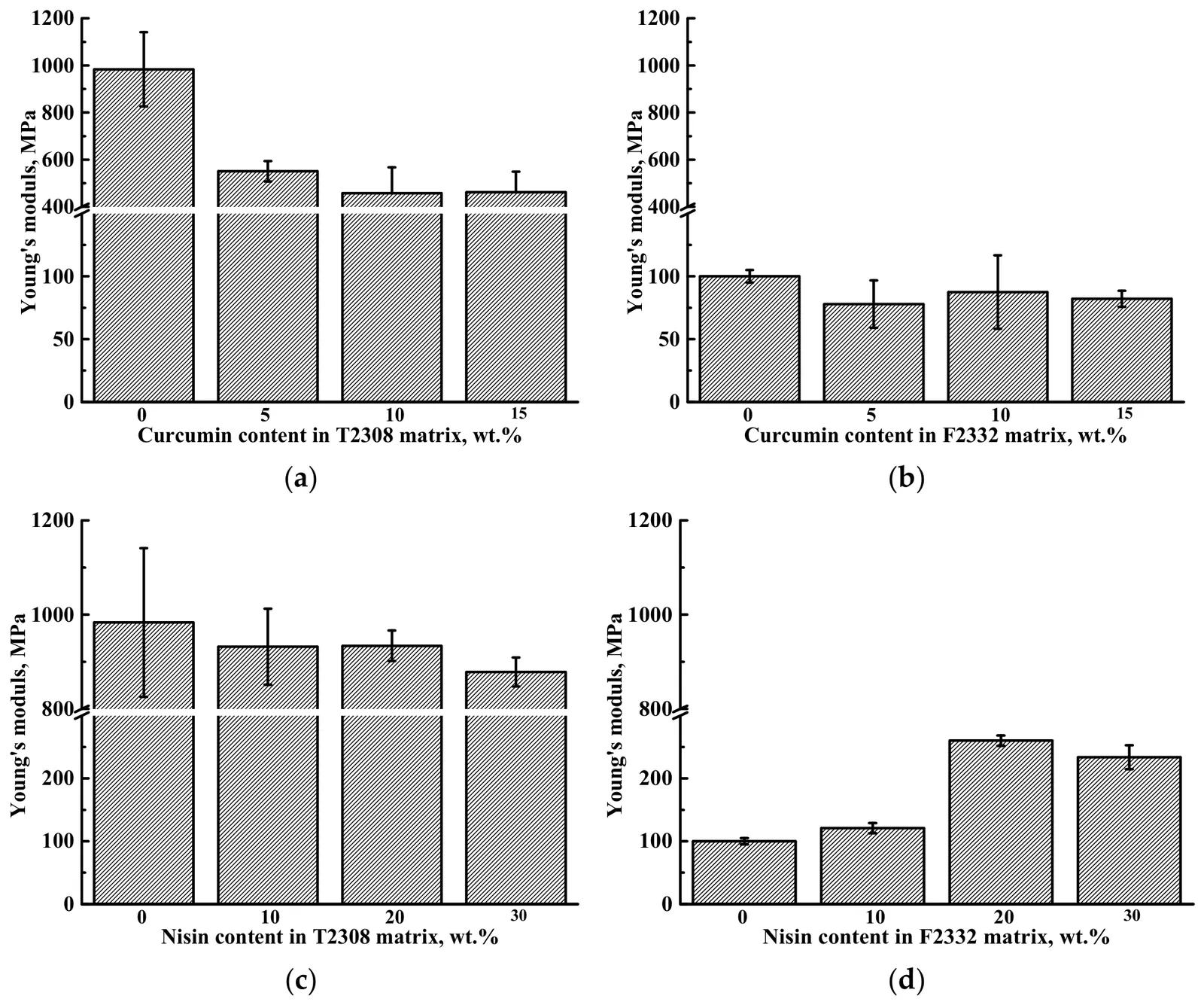
Young’s modulus from DMA of the T2308 and F2332 films, both without and with various concentration of (**a**,**b**) curcumin and (**c**,**d**) nisin, respectively.

The mechanical data obtained can exhibit considerable variability, primarily due to fundamental differences in the operating principles of the measurement devices, the modes of loading applied, and the scales of deformation involved. Specifically, the testing machine typically performs static or quasi-static tests, such as slow tensile or compressive tests until failure, whereas DMA applies periodic, low-amplitude sinusoidal loads to assess viscoelastic properties under conditions that closely mimic real-world operating environments. When analyzing additives with intended mechanical effects—such as curcumin and nisin—these compounds are recognized for their ability to influence mechanical behavior through mechanisms such as plasticization, phase separation, or interfacial modifications. Their impact is more evident in morphological changes (as confirmed by SEM, Figure 3), making it essential to characterize the effects of varying concentrations using DMA, which effectively captures viscoelastic transitions. This, in turn, facilitates a deeper understanding of how these additives modify the polymer matrix’s mechanical response in terms of Young’s modulus (Figure 5).

In both series, the incorporation of curcumin (Figure 5a,b) and nisin (Figure 5c,d) resulted in a notable decrease in Young’s modulus compared to the unmodified matrices, although the extent and magnitude of the reduction differed markedly be-tween the two systems. For T2308 films, the introduction of curcumin led to a sharp reduction in Young’s modulus from 983 MPa in the control sample to about 551 MPa even at the lowest additive content (5 wt.%), and further addition up to 15 wt.% did not produce significant further changes (to approximately 460 MPa), indicating a threshold effect. In contrast, F2332 films exhibited considerably lower initial stiffness (100 MPa for the control), with curcumin addition causing a moderate and incremental decrease, indicating a more gradual plasticization or structural disruption with increasing curcumin concentration.

An analogous matrix-dependent effect was observed upon nisin addition. For T2308, the modulus slightly decreased with nisin up to 10–30 wt.% (from 984 to 878 MPa). Conversely, in F2332 films, the effect was more pronounced: Young’s modulus increased with higher nisin content, rising from 100 MPa (unmodified) to about 234 MPa at 30 wt.%, indicating a possible reinforcing interaction or structuring effect specific to this polymer-additive system. SEM micrographs confirm that nisin is poorly integrated into the T2308 matrix: as its concentration increases, the degree of layering increases and the structural homogeneity decreases (Figure 3). The opposite picture is observed for the F2332 samples, where nisin molecules are more evenly distributed within the PLA/PBAT matrix but from agglomerates on the surface. The F2332 samples have a higher PBAT content than the T2308 samples, and, accordingly, more free volume remains for nisin to be incorporated. Collectively, these results underscore the critical role of matrix–additive compatibility and highlight opportunities for fine-tuning the mechanical properties of films via targeted selection of both blends and additives.

**Figure 6:**
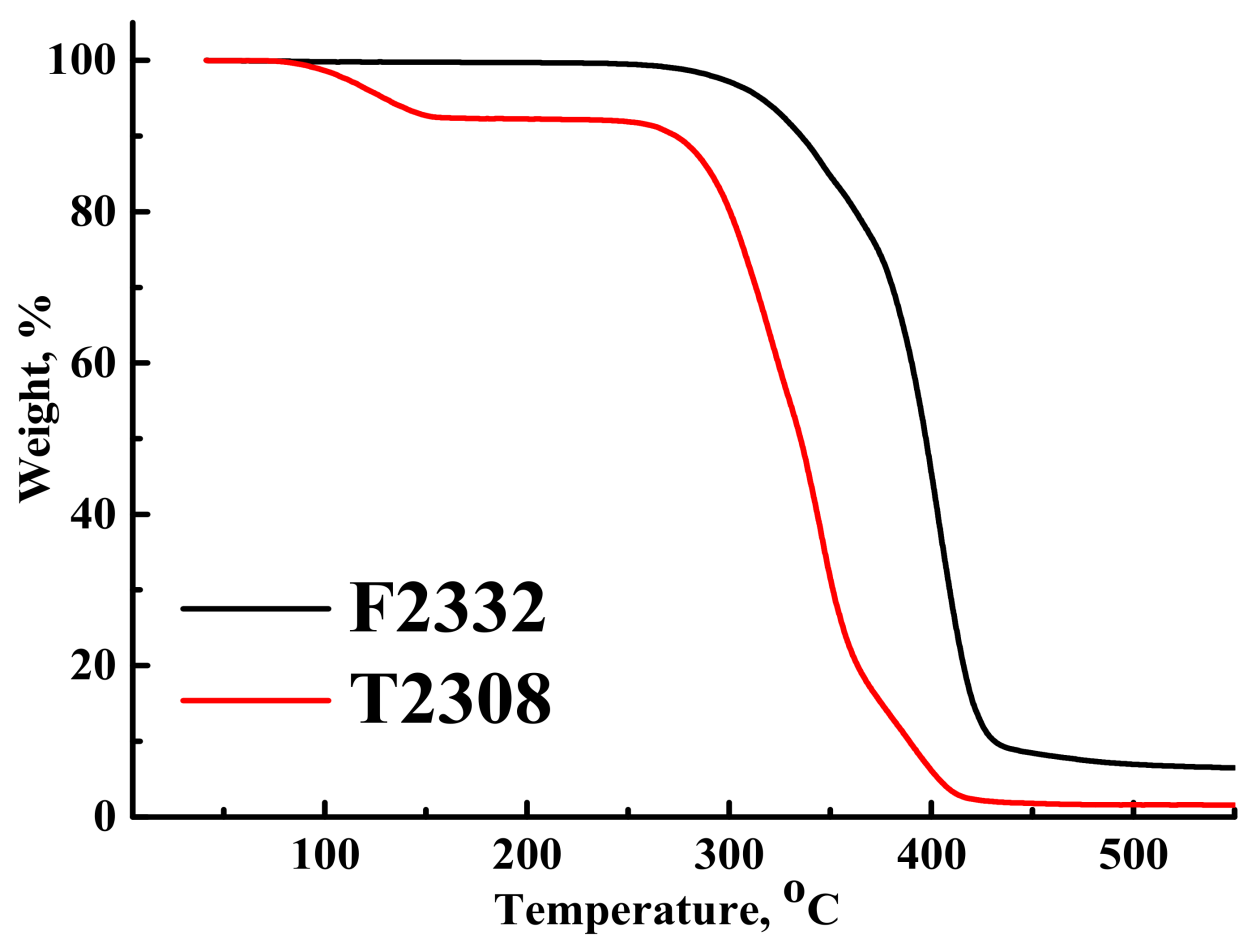
TG curves for the studied T2308 and F2332 films.

The thermal properties of the films from pristine T2308 and F2332 were studied by TGA to demonstrate the difference in PLA/PBAT composition. Figure 6 displays the thermogravimetric (TG) curves for the samples.

The TGA of the T2308 film shows an initial weight loss of about 10% near 150 °C, followed by rapid and nearly complete degradation between 280 and 400 °C, corresponding to the melting and thermal decomposition temperatures of PLA. In contrast, the F2332 film demonstrates greater thermal stability, remaining stable up to 300 °C, with degradation occurring between 320 and 450 °C, which is characteristic of the PBAT phase. The improved thermal stability of the F2332 film can be attributed to its morphological structure: PBAT forms the continuous phase, while PLA exists as dispersed domains within this matrix. Due to the lower PLA concentration and its dispersion within the PBAT network, the degradation of PLA is not observed in the TG curve for the F2332 sample.

**Figure 7:**
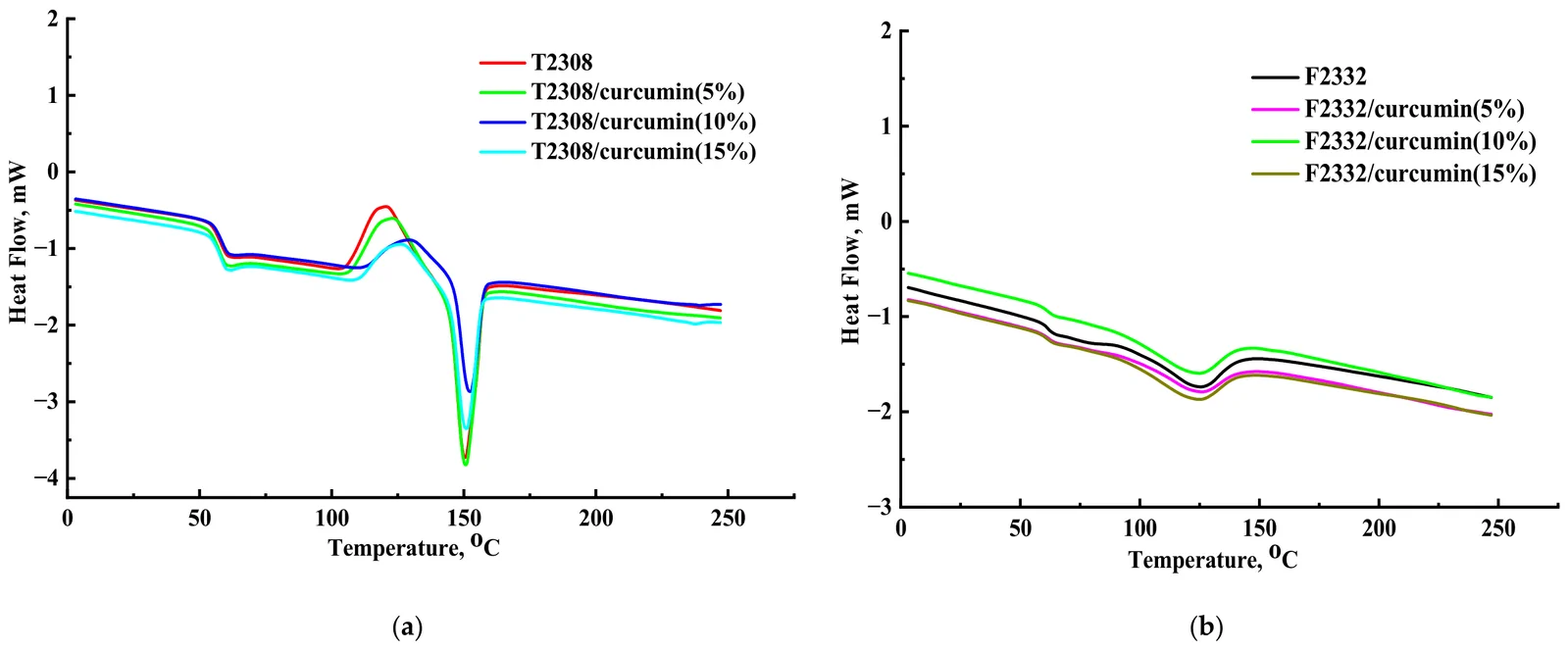
DSC curves for the second heating scan for the films (**a**) from T2308 and (**b**) F2332 with curcumin.

**Figure 8:**
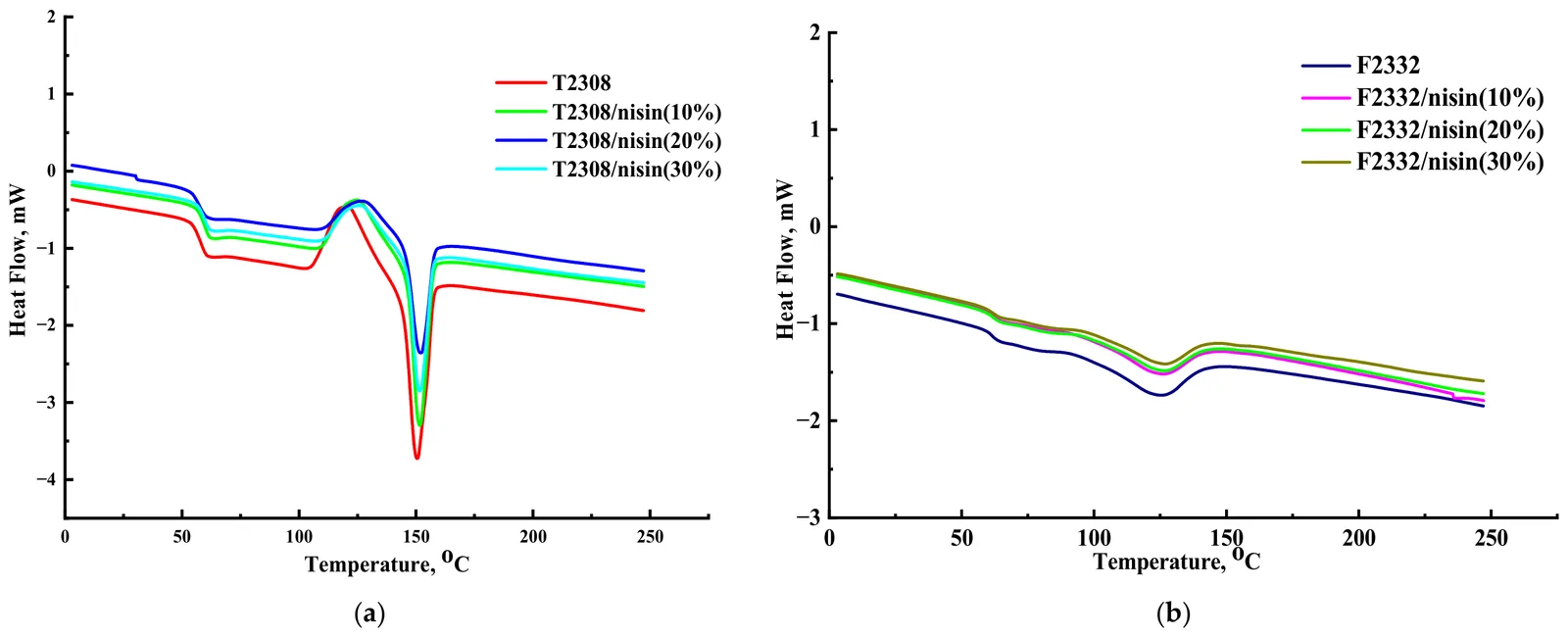
DSC curves for the second heating scan for the films (**a**) from T2308 and (**b**) F2332 with nisin.

**Figure 9:**
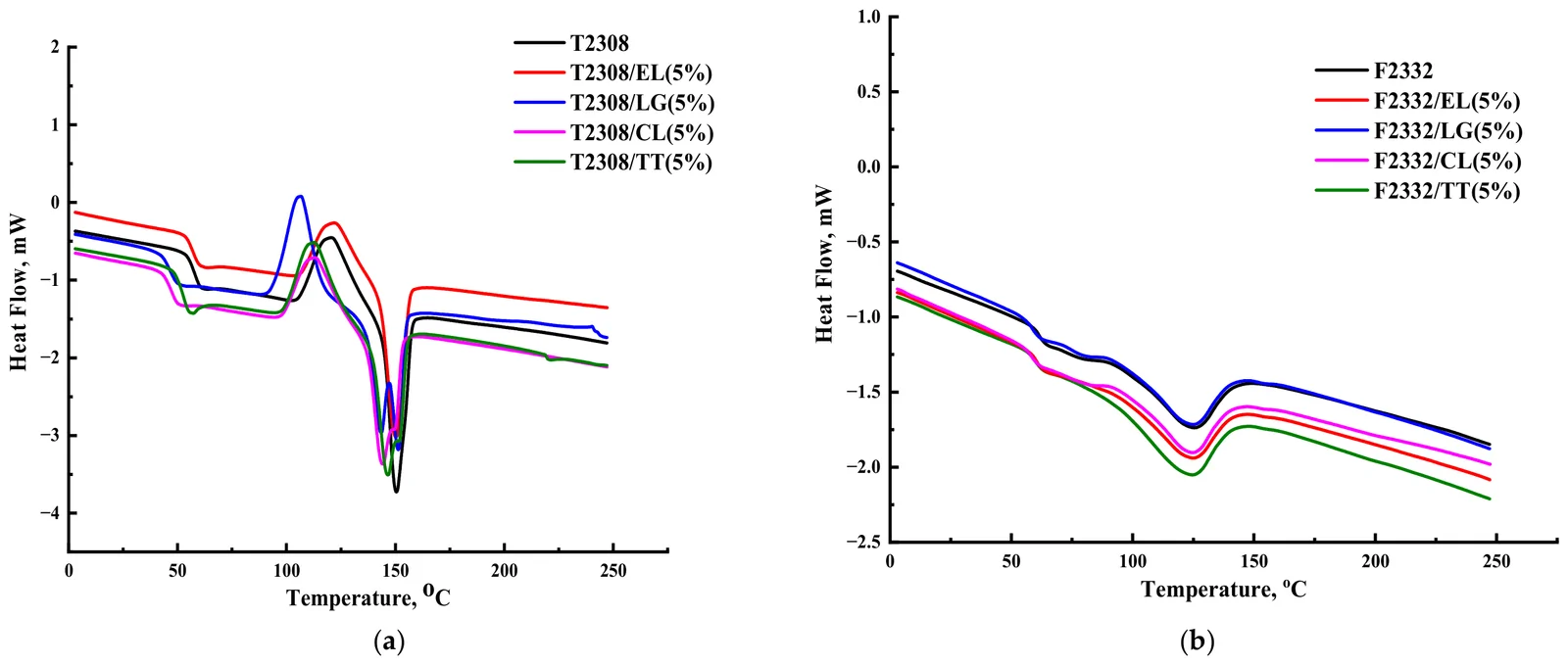
DSC curves for the second heating scan for the films (**a**) from T2308 and (**b**) F2332 with oils.

DSC was used to examine the thermal properties and polymer–additive compatibility of the formulations; the findings for films with curcumin, nisin, and oils are presented in Figure 7, Figure 8 and Figure 9, respectively. DSC curves for the first heating scan for the films are presented in Figure S1 of the Supplementary Materials.

When comparing T2308 and F2332 samples, clear distinctions in their thermograms are evident. T2308 films exhibited sharper and more intense melting transitions, indicative of higher crystallinity and a more defined melting event. The second scan allowed the determination of the glass transition temperatures (Tg) for PLA, which were 59–62 °C [46]. Upon further heating, the T2308 film displays an exothermic peak between 120 and 130 °C. This peak is associated with cold crystallization corresponding to PLA phase. This event occurs because the rapid cooling rate applied prior to the second scan kinetically restricts PLA chain ordering, yielding a predominantly amorphous matrix that recrystallizes upon heating. During cooling, racemic PLA chains (containing both D- and L-enantiomers) often fail to form ordered crystalline domains due to stereochemical irregularities, resulting in a predominantly amorphous structure that lacks a distinct melting transition. This cold crystallization is immediately followed by a sharp endothermic melting peak at ~150–165 °C, characteristic of the PLA crystalline domains. In contrast, the F2332 matrix exhibits broader, less intense thermal transitions, with Tg values at ~58 °C (PLA phase). Due to the lower PLA content and its fine dispersion within the continuous PBAT matrix, cold crystallization and subsequent melting events in F2332 are considerably suppressed. Consequently, no distinct melting event is detected in the second scan, reflecting the slow cold crystallization kinetics of the PLA phase rather than a shift in its melting temperature. This observation is also attributed to the stereocomplexation behavior of D,L-PLA mixtures [47].

DSC was employed to investigate the thermal transitions of T2308 and F2332 matrices in the presence of curcumin at concentrations of 5, 10, and 15 wt.% (Figure 7). During the second heating cycle, the melting peak of T2308 was apparent, with all curcumin-containing samples exhibiting reduced peak intensities and a marginal shift to lower temperatures relative to the neat T2308 film. This indicates that the effect of curcumin is at least partially retained after the thermal history imparted by the first heating, reflecting the persistent influence of curcumin on the polymer morphology. The incorporation of the additive into the mixture results in a reduction in the polymer content within the film matrix. As the polymer concentration decreases, the number of crystalline regions diminishes accordingly, which is reflected in a subsequent decrease in the melting enthalpy.

In contrast, the DSC traces obtained for F2332-based films with and without curcumin showed different thermal behavior. The second heating scans for F2332 displayed broader, less intense endothermic events. The addition of curcumin led to only subtle changes in the DSC profiles, with marginal reductions in peak intensity and slight shifts in transition temperatures. This demonstrates that curcumin has a less pronounced impact on the crystalline structure of F2332 than on that of T2308, likely due to differences in the polymeric blend composition.

Figure 8 displays the second heating DSC profiles for films incorporated with nisin (10, 20, and 30 wt.%). For the T2308 matrix, increasing the nisin concentration leads to a systematic suppression of the cold crystallization corresponding to the PLA phase and a reduction in the area of the PLA melting endotherm. This trend confirms that high loadings of the peptide-based additive disrupt polymer chain mobility and hinder regular PLA chain packing. In the F2332 matrix, the thermal profiles remain broad and featureless across all nisin concentrations, confirming that the predominantly amorphous character of F2332 is largely insensitive to nisin loading.

DSC analysis was also performed to evaluate the impact of various essential oils on the thermal behavior of films based on T2308 and F2332 matrices. For both T2308 and F2332 matrices, the decrease in melting peak intensity correlates with a reduction in crystalline fraction, attributable to the change in blend composition from pure polymer to mixtures containing varying oil/polymer ratios, which effectively diminishes the polymer content and thus the crystalline regions. Notably, the shifts in the glass transition temperature (Tg) are particularly significant, reflecting the extent of plasticization imparted by the oils.

The addition of all oils results in a measurable decrease in Tg, with the magnitude varying depending on the oil’s molecular structure. Oils like EL (Eucalyptol), LG (Citral), and TT (Terpinen-4-ol), which lack aromatic rings and consist predominantly of flexible molecules with mainly sp^3^ hybridized carbons, exhibit a stronger plasticizing effect due to their conformational flexibility and ability to freely rotate around σ bonds. This flexibility enhances their capacity to penetrate within the polymer network, disrupting intermolecular interactions and increasing free volume, thus lowering Tg. Conversely, CL oil, with eugenol as a principal component, contains an aromatic benzene ring. The aromatic structure introduces rigidity and the potential for reinforcing interactions within the polymer matrix, which can counterintuitively lead to localized stiffening or a less pronounced shift in Tg. The aromatic ring acts as a rigidifying unit when embedded in accessible regions of the film, reinforcing the network and resisting the plasticizing effect. Overall, the molecular structure and main components of the oils fundamentally influence their interaction with the polymer matrices. The flexible, non-aromatic compounds favor increased chain mobility and lower Tg, while the aromatic eugenol in CL can act as a rigidifying agent, partially resisting the softening effect. These molecular differences are reflected in the temperature shifts in the glass transition, with the matrix’s specific composition and its susceptibility to reinforcement or plasticization being dictated by the chemical nature of the added oil components.

Furthermore, the plasticizing efficiency of the incorporated essential oils was quantitatively evaluated by correlating the shift in the glass transition temperature with the tensile properties. The addition of 5 wt.% of flexible oils (EL, LG, TT) into T2308-based films led to a systematic decrease in Tg, which quantitatively correlated with an increase in the elongation (Table S5 of the Supplementary Materials). This relationship confirms that the highly mobile terpenoid molecules effectively increase the free volume of the polymer blend, lowering the energy barrier for macromolecular chain sliding under mechanical loading.

Based on the comprehensive data, it can be concluded that the additive type significantly influences the properties of the T2308 and F2332 matrices, with effects that are strongly dependent on the specific blend composition and the nature of the additive introduced. T2308 exhibits a higher initial Young’s modulus, indicating greater intrinsic stiffness, but this value is reduced upon the incorporation of various additives such as curcumin, nisin, and essential oils; these additives tend to soften the material and enhance ductility.

Thermally, TGA and DSC analyses demonstrate that T2308 possesses a more crystalline structure with distinct melting transitions, which are significantly affected by the incorporation of additives such as curcumin and oils, leading to reductions in crystallinity and melting enthalpy. In contrast, F2332 exhibits lower crystallinity and broader, less defined thermal transitions, with additive effects being subtler and primarily involving plasticization rather than crystallinity disruption. The presence of oils generally causes minor shifts in thermal transition temperatures in both matrices, with T2308 showing more noticeable changes due to its more ordered crystalline structure.

Overall, the data reveal that T2308, with its higher mechanical stiffness and crystallinity, responds more markedly to additive-induced modifications, which can be used to tailor its properties for specific applications. F2332, being more amorphous and flexible, shows less drastic but still significant adjustments mainly related to its plasticization and morphology. These findings underscore the importance of selecting the appropriate matrix and additive combination to achieve the desired mechanical and thermal performance in packaging or biomaterial applications, emphasizing the crucial role of matrix–additive compatibility in efficiently tuning such properties.

### 3.3. Physicochemical Properties

The films were characterized by measuring the water contact angle, moisture absorption (MA), and water vapor permeability (WVP) (Table 3, Table 4, Table 5 and Table 6), because these metrics provide critical information on surface wettability, liquid uptake, barrier performance, and resistance—properties that collectively determine the films’ suitability for packaging applications.

In the case of curcumin, increasing its content from 5 to 15% resulted in a general decrease in water vapor permeability (WVP) among the modified films, indicating a slight improvement in barrier performance at higher loadings, although overall permeability remained within a comparable range. This trend was accompanied by a reduction in MA, which decreased, consistent with the hydrophobic nature of curcumin. Simultaneously, the water contact angle decreased from about 79° in control films to 65–66°, signaling increased surface wettability contrary to the expected hydrophobic behavior—possibly due to morphological surface changes or additive distribution effects, which may be related to the hydrophilic nature of turmeric, from which curcumin is derived.

The addition of nisin, evaluated at 10–30 wt.% yielded a significant reduction in WVP, whereby the permeability decreased to approximately 1.48–1.63 × 10^−11^ for T2308 samples, with a concomitant slight increase in MA at higher concentrations. Interestingly, films with nisin demonstrated a decrease in water contact angle from 79° to around 65°, implying an increase in surface hydrophilicity. Conversely, in the F2332 matrix, nisin’s inclusion did not substantially alter WVP or MA.

Incorporation of zinc oxide nanoparticles at loadings of 5–10 wt.% generally resulted in a slight increase in WVP, while MA remained minimal, often at negligible levels. The films exhibited reduced water contact angles upon ZnO incorporation [38], indicating elevated surface wettability, which is consistent with the hydrophilic characteristics of ZnO particles. Morphological analysis (Figure 3) demonstrates effective dispersion of nanoparticles within the matrices, though some clustering was evident in the SEM micrographs.

Oils derived from EL, LG, TT, and CL at 5 wt% each affected the films differently. EL oil notably increased micro-void formation and porosity (Figure 3), resulting in higher WVP and a decrease in the water contact angle, indicative of enhanced permeability and surface wettability. LG oil promoted the development of honeycomb-like structures on the surface morphology and further reduced barrier properties further due to increased surface roughness. Films with TT oil showed a more balanced profile, maintaining relatively lower WVP values and similar contact angles. Conversely, CL oil, characterized predominantly by eugenol, resulted in decreased WVP values and the lowest contact angles, indicating improved barrier properties and surface wettability, likely owing to its aromatic components, which reinforce the polymer network.

Thus, the nature and concentration of additives distinctly modulate the films’ barrier, surface, and physicochemical properties. Generally, hydrophobic additives such as curcumin and certain essential oils tend to reduce WVP and MA, while hydrophilic particles like ZnO increase wettability. Surface morphology, microstructure, and additive–polymer interactions fundamentally govern these properties, emphasizing the importance of matrix composition and additive type in designing biodegradable films.

**Figure 10:**
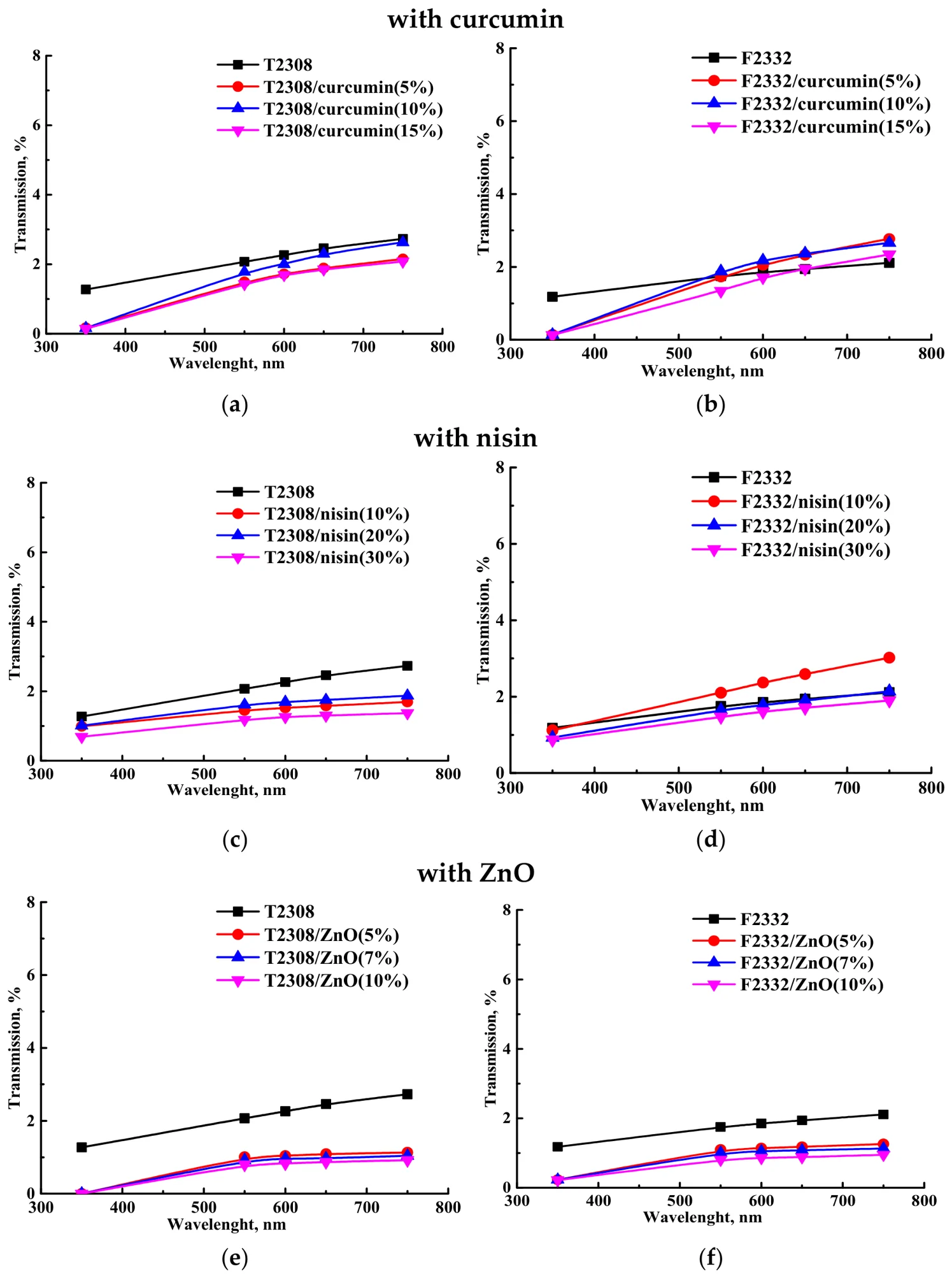

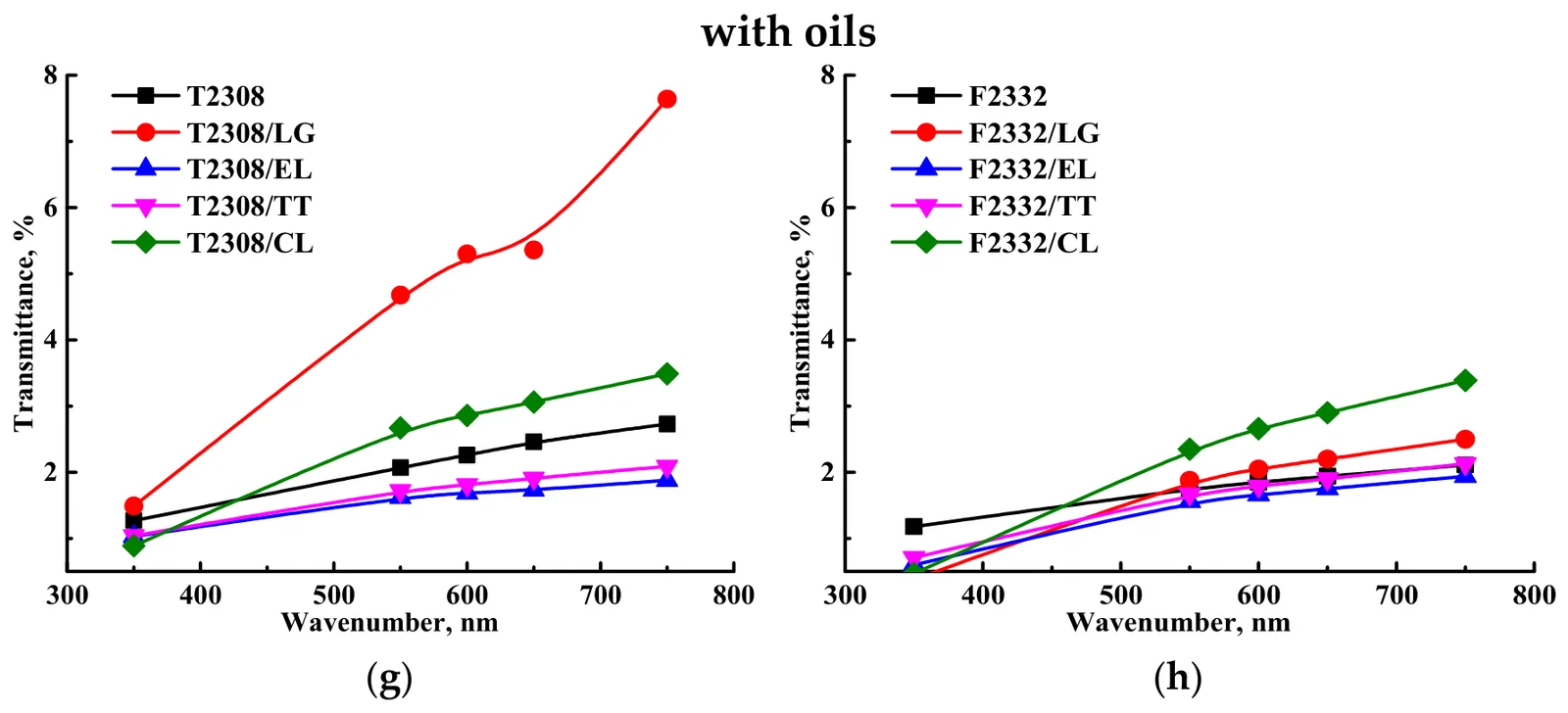
Light transmittance of the films from (**a**,**c**,**e**,**g**) T2308 and (**b**,**d**,**f**,**h**) F2332.

The transmittance of the films was evaluated across a spectral range from 350 nm to 750 nm (Figure 10) to analyze their optical performance under both visible and ultraviolet (UV) light exposure. This wavelength interval encompasses the majority of the visible spectrum and is particularly influential in maintaining product quality. Specifically, visible light spans from approximately 380–400 nm at its lower boundary to around 760–780 nm at its upper limit, with the potential for radiation to penetrate up to roughly 810 nm. The UV spectrum, on the other hand, covers wavelengths from 100 nm to 400 nm and is subdivided into three regions: UV-A (315–400 nm), UV-B (280–315 nm), and UV-C (100–280 nm). When sunlight travels through Earth’s atmosphere, most UV-C and approximately 90% of UV-B radiation are absorbed by atmospheric gases such as carbon dioxide, water vapor, ozone, and oxygen. As a result, the UV radiation that ultimately reaches the surface predominantly consists of UV-A rays, with only a minimal presence of UV-B radiation.

In the control samples without additives, the films from T2308 and F2332 matrices exhibited high transmittance within the visible spectrum, with notable decreases in the UV region, particularly below 400 nm, reflecting their inherent UV-shielding properties.

In films containing curcumin at varying concentrations (5–15 wt.%), a clear trend was observed: increasing curcumin content led to a substantial reduction in transmittance across the entire spectrum, which was especially pronounced at wavelengths below 500 nm, signifying enhanced UV-blocking capability. Similarly, F2332 films showed a comparable reduction, albeit with slightly higher transmittance values at the same additive levels, indicating that curcumin’s absorption properties effectively attenuate UV radiation but also influence visible light transmission.

Moreover, to demonstrate the pH-responsive behavior of curcumin-modified films, samples were immersed in HCl (1 M), NaOH (1 M) and a set of buffer solutions spanning pH of 1.68, 3.56, 4.01, 6.86, 9.18, and 12.45. The color of the films and solutions was assessed every 60 min for up to 24 h (Table S6 in the Supplementary Materials). Significant color changes were observed in weakly alkaline and alkaline media due to tautomeric transitions and deprotonation. The color change in the indicator is clearly visible under mildly to strongly alkaline conditions, indicating that the pH-indicating properties of curcumin can be effectively utilized to monitor the quality of foods that undergo an alkaline pH shift during spoilage. During the spoilage of various meat products, enzymes and microbial activity degrade proteins, leading to the accumulation of volatile amines and other nitrogenous compounds, which increase the pH of the meat product [48,49].

The incorporation of nisin at different concentrations into the films resulted in modest decreases in transmittance, primarily within the UV and shorter visible wavelengths. At 30 wt.% nisin, the transmittance was reduced relative to the control, with the most noticeable effects observed below 400 nm. This indicates that nisin contributes additionally to UV shielding, although to a lesser extent than curcumin.

Additive incorporation of ZnO nanoparticles led to a further decline in transmittance, which was especially notable in the UVA and UVB regions (315–400 nm). The films with 10 wt.% ZnO displayed a transmittance reduction of up to 30–40% compared to the pristine films, emphasizing the role of ZnO as an effective UV absorber. In the visible range, the transmittance remained relatively high, thus preserving the film’s transparency for practical applications.

Furthermore, films embedded with natural oils showed diverse effects on transmittance. Oils containing aromatic or phenolic compounds, such as CL oil, further decreased transmittance in the UV and blue regions, contributing to UV attenuation and potential antioxidant properties. Films with LG and EL oils showed moderate reductions in UV transmittance, while TT oil had a less pronounced impact, maintaining higher clarity in the visible spectrum.

Overall, the data underscore that increasing the content of additives, particularly curcumin and ZnO, enhances UV protective properties via absorption mechanisms, albeit with concomitant reductions in visible transmittance. T2308 samples tend to exhibit more substantial transmittance attenuation at equivalent additive concentrations compared to F2332, likely due to differences in their microstructure and coloration. These findings highlight the importance of optimizing additive type and dosage to balance transparency and protective functionality, thereby tailoring the films for specific packaging applications that require UV protection without compromising optical clarity.

A comprehensive analysis of the presented data indicates that the selection of optimal concentrations for each additive is critical to balancing the barrier, mechanical, and functional properties of the final films. It is advisable to maintain turmeric (curcumin) content at a relatively low level (approximately 5 wt.%) since higher concentrations tend to impair the mechanical integrity of the films. Despite this, a 5% concentration is sufficient to confer both visual and functional responsiveness to pH variations, providing a functional advantage for smart packaging applications. Nisin, a potent antimicrobial agent, should be incorporated at modest levels (around 10 wt.%). When combined with zinc oxide (preferably at higher concentrations, up to 10 wt.%), this approach can achieve an antimicrobial effect while simultaneously reducing material costs. Zinc oxide’s role is especially crucial, as it enhances hydrophobicity, an essential attribute for moisture resistance when packaging products exposed to atmospheric humidity.

Among the studied essential oils, CL oil was identified as the most promising candidate due to its ability to significantly improve the Young’s modulus and barrier properties—in particular, by reducing vapor permeability. Its addition, combined with other components, can help tailor the composite’s mechanical strength and moisture resistance. Moreover, CL possesses a pleasing aroma that can mask undesirable odors associated with product spoilage, and it also exhibits reported antitumor activity.

The strategic combination of low to moderate concentrations of turmeric, nisin, zinc oxide, and CL oil offers promising avenues for developing environmentally friendly packaging materials. Such formulations can provide enhanced barrier properties, mechanical robustness, antimicrobial protection, and sensory attributes, making them highly suitable for smart packaging solutions. Future research should focus on fine-tuning these concentrations, understanding additive interactions at the molecular level, and assessing long-term stability and efficacy in real-world applications.

### 3.4. Film Biodegradability

**Figure 11:**
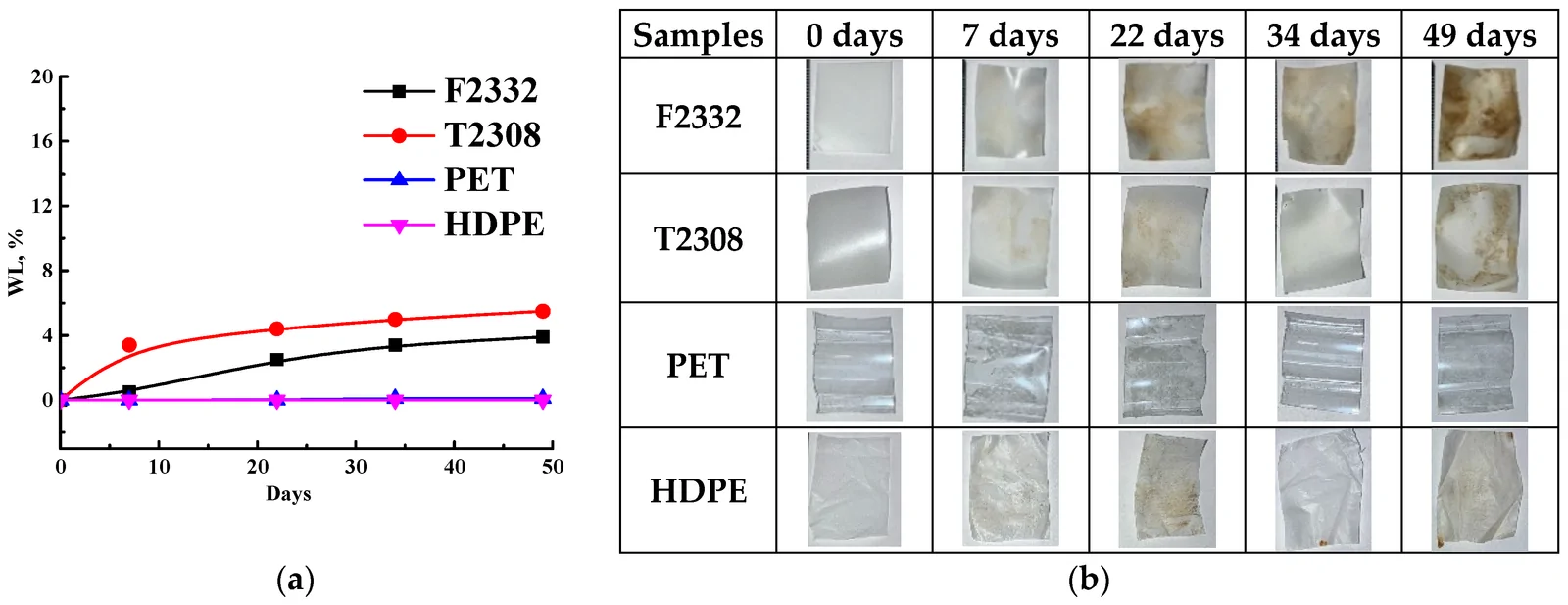
(**a**) Weight loss curves and (**b**) photos of films from T2308 and F2332 under composting conditions.

To assess the biodegradation behavior of the T2308 and F2332 films, samples were fabricated following the original preparation protocol without incorporating any additives. For comparative purposes, the biodegradation of reference materials—namely polyethylene terephthalate (PET) and high-density polyethylene (HDPE)—was also examined under identical conditions. The investigation is still in progress, with preliminary findings reported after 49 days of incubation, based on weekly sampling sessions. Figure 11 presents both the weight loss data and visual changes in films of the T2308 and F2332 subjected to composting conditions over a 49-day period (Figure 11).

The data reveal that the F2332 films exhibit more gradual degradation, with weight loss reaching approximately 4% after 49 days. In contrast, T2308 films show a slightly higher rate of degradation rate, attaining around 6% weight loss at the end of the period. No noticeable weight change was observed for PET and HDPE over 49 days. Significant degradation of these polymers under natural conditions can take 450–1000 years, which is a significant drawback. Even when simulating soil conditions, completely eliminating UV radiation is impossible, so its minimal impact on degradation processes should be taken into account.

The F2332 samples maintain their integrity with slight surface roughening over time, and after 49 days, they display noticeable discoloration and surface erosion. The T2308 films demonstrate a similar trend but with more pronounced surface deterioration and fragmentation. Conversely, PET and HDPE remain largely unchanged throughout the experiment, retaining their original appearance. All samples, with the exception of PET, acquire a characteristic yellow tint. The intensity of this tint increases over the course of the experiment. The yellowing of materials is caused by the breakdown of the main polymer chain, resulting in the formation of unsaturated bonds due to disproportionation. Further breakdown leads to the formation of more such bonds and potential conjugation, which results in light absorption and the development of the characteristic color. For films, the primary degradation mechanism under the conditions of this study is hydrolytic degradation—the cleavage of ester bonds in the polymer backbone when PLA is exposed to moisture, resulting in the formation of linear polymers with carboxyl and hydroxyl groups [50]. The mechanism causing yellowing in polyethylene and PLA was described in detail in the work [51], the authors attributed this observation to changes in the content of carbonyl groups (C=O) and the ratio of carbon and oxygen in the polymers. These observations highlight the differential biodegradation profiles of the tested materials, with F2332 and T2308 showing significant biological breakdown, whereas PET and HDPE are comparatively stable during the study period.

**Figure 12:**
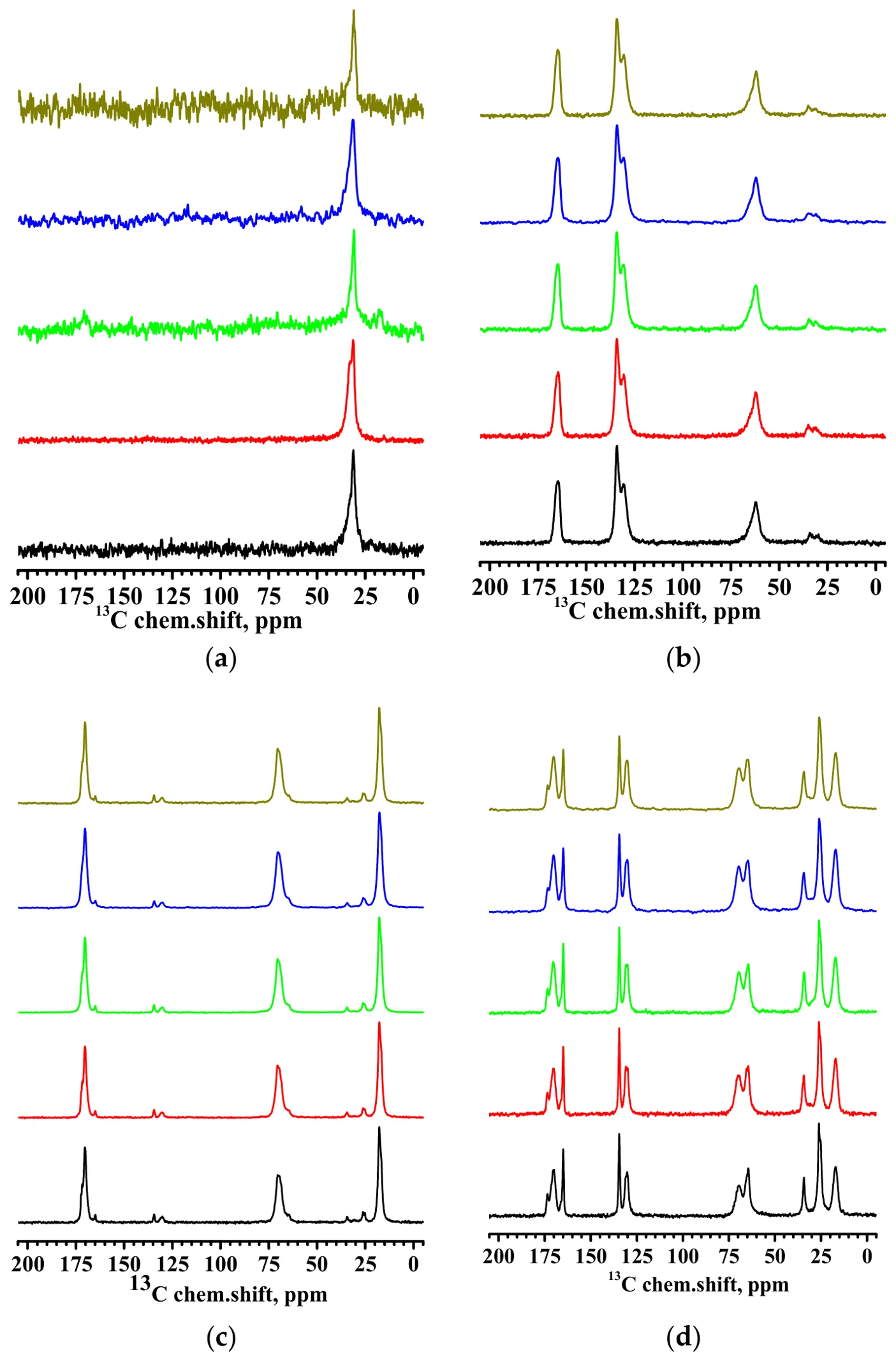
^13^C NMR spectra of (**a**) HDPE, (**b**) PET, (**c**) T2308, and (**d**) F2332 (bottom–up: 0, 7, 22, 34, 49 days).

Figure 12 presents the ^13^C NMR spectra of the investigated films during biodegradation under composting conditions. The observed resonances are in agreement with literature data (HDPE: [52]; PET: [53]; PLA: [54,55]; PBAT: [56]). Throughout the experiment, none of the spectra exhibited significant changes, indicating that no chemical transformations occurred in any of the polymers during biodegradation. In particular, the HDPE and PET spectra remained essentially identical, confirming their structural stability under the experimental conditions. Samples T2308 and F2332 are mixtures of PLA and PBAT polymers at different ratios, as reflected by the redistribution of the integrated spectral intensities. The spectra of T2308 exhibit prominent peaks from PLA and lower-intensity peaks from PBAT, whereas in F2332, the intensities of peaks from both polymers are comparable. Analysis of spectral variations after soil exposure over different periods reveals a broadening of all spectral components, indicating increased disorder within the polymer structures. Notably, this broadening affects spectral features associated with both PLA and PBAT components.

## 4. Conclusions

In this study, the matrices based on Ecovio^®^ blends, specifically T2308 and F2332 (PLA/PBAT with different ratios) were effectively functionalized with various agents, such as nisin, curcumin, zinc oxide nanoparticles, and essential oils (clove, eucalyptus, lemongrass, and tea tree) and systematically characterized to develop biodegradable packaging films. FTIR spectroscopy confirmed successful incorporation of additives into the polymer matrix and identified interactions, including hydrogen bonding and potential covalent-like interactions, which influence the thermal behavior assessed by DSC and TGA. Morphological analysis via SEM revealed that additive integration induces significant microstructural modifications, which are matrix-dependent. T2308 exhibited a denser, more heterogeneous structure, while F2332 presented a more homogeneous and flexible morphology. Mechanical testing, complemented by DMA, showed that the Young’s modulus ranged from approximately 983 MPa in unmodified T2308 to about 100 MPa in F2332, with additive incorporation generally decreasing stiffness. Physicochemical characterization indicated that hydrophobic additives such as curcumin and certain essential oils reduce water vapor permeability and moisture absorption, thereby improving barrier performance. Conversely, hydrophilic zinc oxide nanoparticles marginally increased surface wettability. Optical assessment confirmed that curcumin and zinc oxide effectively attenuate UV transmittance, offering protection against UV radiation without significant loss of visible transparency—a crucial characteristic for packaging applications.

Based on the comprehensive analysis, the optimal choice of essential oil for incorporation into the T2308 and F2332 matrices was clove oil (CL), which demonstrated a balanced enhancement of mechanical strength and barrier properties, attributable to its aromatic compounds such as eugenol, which contributed to network reinforcement. For T2308, with higher crystallinity and stiffness, optimal additive levels are 10–15 wt.% nisin, providing effective antimicrobial activity while reducing Young’s modulus. Curcumin (5–15 wt.%) offers UV protection, with DSC indicating decreased crystallinity. ZnO (5–10 wt.%) enhances barrier and antimicrobial properties, as confirmed by FTIR and SEM, which showed well-dispersed particles. In F2332, a more flexible, amorphous matrix, optimal levels are 10–20 wt.% nisin and 5–10 wt.% ZnO, with FTIR, XPS and SEM confirming their uniform distribution. Mechanical tests show a Young’s modulus of around 100 MPa decreasing slightly with additives. Curcumin (5–10 wt.%) improves UV blocking, with minimal thermal changes due to its amorphous nature. Overall, T2308 is more sensitive to crystallinity-disrupting additives, whereas F2332 responds with greater flexibility. Both matrices, with optimized additive levels, achieve suitable properties for packaging applications.

Future research should explore the effects of combined additives via factorial designs and multivariate optimization to further fine-tune the films’ properties. Molecular-level studies, such as spectroscopic analyses and molecular modeling, are necessary to further elucidate additive–matrix interactions. The development of optimized formulations can lead to advanced, environmentally sustainable packaging solutions that meet stringent performance criteria, expanding the scope of biodegradable films for packaging applications.

## Figures and Tables

**Table 1:** The characteristics of commercial T2308 and F2332 from ref. [16].

T2308	F2332
Opaque, semi-crystalline structure	Translucent white color
High but controlled vapor permeability rate	Moisture resistance (e.g., required for the production of organic waste bags)
High melt strength	High melt strength
Average barrier to oxygen	Good mechanical properties
Good heat resistance during processing up to 205 °C	Good heat resistance up to 230 °C
Good processability on traditional sheet extrusion lines	Wide welding interval required for welding layers of multilayer film structures
Suitable for sealing	Good processability on bag making equipment

**Table 2:** The main substances contained in each oil.

Oil	CL	TT	EL	LG
The main component	Eugenol [41]	Terpinen-4-ol [41]	Eucalyptol [41]	Citral [42]
Structure	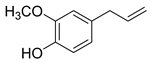	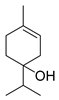	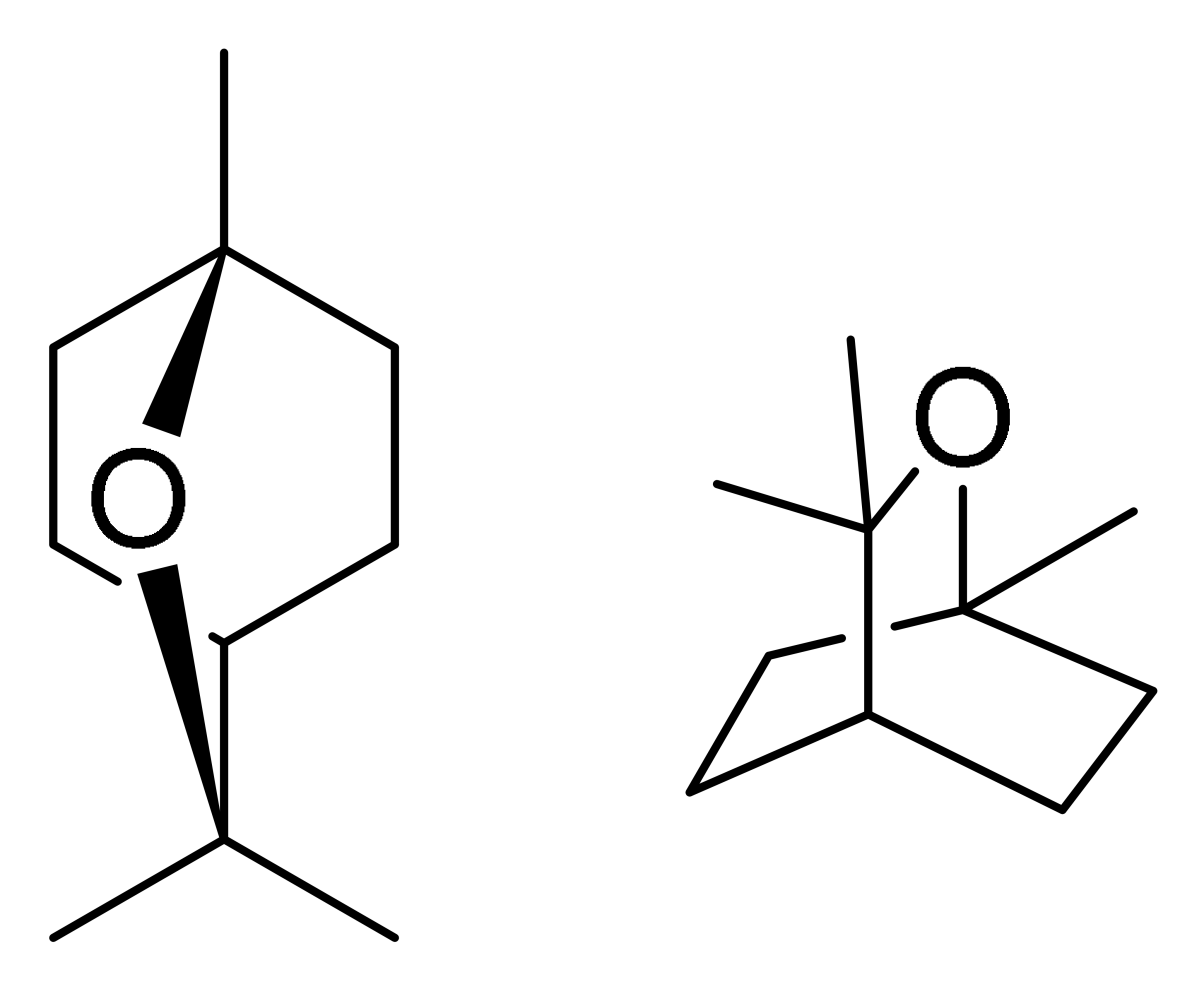	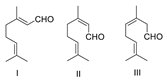

**Table 3:** Water vapor permeability (WVP), moisture absorption (MA) and water contact angle for the films with curcumin.

Sample	WVP × 10^−11^, g/(s·m·Pa)	MA, %	Water Contact Angle, °
T2308	2.08 ± 0.05 c *	0.3 ± 0.2 a	79 ± 1 a
T3208/curcumin (5%)	3.72 ± 0.09 a	0.2 ± 0.2 a	67 ± 1 b
T3208/curcumin (10%)	3.02 ± 0.13 b	0.2 ± 0.2 a	66 ± 1 b
T3208/curcumin (15%)	3.27 ± 0.07 ab	0.1 ± 0.2 a	65 ± 1 b
F2332	2.28 ± 0.09 c	1.2 ± 0.1 a	80 ± 1 a
F2332/curcumin (5%)	6.33 ± 0.09 a	1.0 ± 0.1 a	70 ± 1 b
F2332/curcumin (10%)	5.30 ± 0.08 b	1.0 ± 0.1 a	66 ± 2 b
F2332/curcumin (15%)	5.16 ± 0.11 b	1.0 ± 0.1 a	59 ± 1 c

* Tukey grouping (lowercase letters).

**Table 4:** Water vapor permeability (WVP), moisture absorption (MA) and water contact angle for the films with nisin.

Sample	WVP × 10^−11^, g/(s·m·Pa)	MA, %	Water Contact Angle, °
T2308	2.08 ± 0.05 a *	0.3 ± 0.2 c	79 ± 1 a
T3208/nisin (10%)	1.63 ± 0.05 b	0.4 ± 0.2 bc	70 ± 1 b
T3208/nisin (20%)	1.59 ± 0.07 b	0.5 ± 0.2 ab	69 ± 1 b
T3208/nisin (30%)	1.48 ± 0.02 c	0.6 ± 0.2 a	65 ± 1 c
F2332	2.28 ± 0.09 a	1.2 ± 0.1 a	80 ± 1 a
F2332/nisin (10%)	2.24 ± 0.13 a	0.4 ± 0.1 c	71 ± 2 b
F2332/nisin (20%)	2.25 ± 0.11 a	0.6 ± 0.2 b	68 ± 1 b
F2332/nisin (30%)	2.22 ± 0.12 a	0.7 ± 0.2 b	61 ± 1 c

* Tukey grouping (lowercase letters).

**Table 5:** Water vapor permeability (WVP), moisture absorption (MA) and water contact angle for the films with ZnO.

Sample	WVP × 10^−11^, g/(s·m·Pa)	MA, %	Water Contact Angle, °
T2308	2.08 ± 0.05 b *	0.3 ± 0.1 a	79 ± 1 a
T3208/ZnO (5%)	2.09 ± 0.16 b	0 b	72 ± 1 b
T3208/ZnO (7%)	2.42 ± 0.20 ab	0 b	68 ± 1 bc
T3208/ZnO (10%)	2.79 ± 0.13 a	0 b	67 ± 1 c
F2332	2.28 ± 0.09 b	1.2 ± 0.1 a	80 ± 1 a
F2332/ZnO (5%)	2.39 ± 0.24 ab	0.1 ± 0.1 b	79 ± 1 a
F2332/ZnO (7%)	2.48 ± 0.34 ab	0.1 ± 0.1 b	75 ± 1 ab
F2332/ZnO (10%)	2.64 ± 0.23 a	0 b	71 ± 2 b

* Tukey grouping (lowercase letters).

**Table 6:** Water vapor permeability (WVP), moisture absorption (MA) and water contact angle for the films with oils.

Sample	WVP × 10^−11^, g/(s·m·Pa)	MA, %	Water Contact Angle, °
T2308	2.08 ± 0.05 b *	0.3 ± 0.2 a	79 ± 1 a
T2308/CL (5%)	1.54 ± 0.13 b	0.3 ± 0.2 a	66 ± 1 c
T2308/TT (5%)	1.70 ± 0.17 b	0.3 ± 0.2 a	68± 1 c
T2308/EL (5%)	2.88 ± 0.13 a	0.3 ± 0.2 a	71 ± 2 b
T2308/LG (5%)	1.87 ± 0.08 b	0.2 ± 0.2 a	72 ± 2 b
F2332	2.28 ± 0.09 c	1.2 ± 0.1 a	80 ± 1 a
F2332/CL (5%)	1.91 ± 0.27 c	0.5 ± 0.1 b	63 ± 1 cd
F2332/TT (5%)	2.75 ± 0.17 ab	0.2 ± 0.1 c	73 ± 1 b
F2332/EL (5%)	2.96 ± 0.27 a	0.2± 0.1 c	63 ± 2 d
F2332/LG (5%)	2.35 ± 0.27 bc	0 d	67 ± 1 c

* Tukey grouping (lowercase letters).

## Data Availability

The original contributions presented in this study are included in the article/Supplementary Materials. Further inquiries can be directed to the corresponding authors.

## Supplementary Materials

The following supporting information can be downloaded at https://www.mdpi.com/article/10.3390/polym18172158/s1: Section S1. Film Characterization. Table S1: C1s, O1s, N1s XPS spectra for films; Table S2: The elemental composition and component ratios for films; Table S3: SEM image with EDX of cross-sectional of films; Table S4: SEM image with EDX of surface of films; Table S5: Maximum elongation and tensile strength; Figure S1: DSC curves for the first heating scan for the films (a,c,e) from T2308 and (b,d,f) F2332 with additives; Figure S2: Keto–enol tautomers of curcumin; Table S6: Photos of the resulting solutions before and after 24 h, showing the color change depending on pH (from left to right: solutions with pH 0.01, 1.68, 3.56, 4.01, 6.86, 9.18, 12.45, 13.80). Section S2. ANOVA and Tukey Test Results.
