# Supplementary material for: Natural and Nanomaterial Additives in Biodegradable PLA/PBAT Films: Towards Advanced Packaging Materials
Source: Polymers (Basel). 2026 Sep 3;18(17):2158. doi: 10.3390/polym18172158 (PMC13568014; doi:10.3390/polym18172158)
Supplement: Supplementary file 1 [file polymers-18-02158-s001.zip › polymers-4470452-supplementary.pdf]

Supporting Information

Natural and Nanomaterial Additives in Biodegradable PLA/PBAT Films: Towards Advanced Packaging Materials

Mariia Dmitrenko [1,*], Ilnur Dzhakashov [1], Daniel Pasquini [2], Anna Kuzminova [1], Anton Mazur [1], Sabu Thomas [3], Rongxin Su [4], Anastasia Penkova [1,*]

1 *St. Petersburg State University, 7/9 Universitetskaya nab, St. Petersburg, 199034, Russia;* st117543@student.spbu.ru (I.D.), a.kuzminova@spbu.ru (A.K.); a.mazur@spbu.ru (A.M.)

2 *Instituto de Química, Universidade Federal de Uberlândia, Campus Santa Mônica, Av. João Naves de Ávila, 2121,Uberlândia, 38400-902, Minas Gerais, Brazil;* daniel.pasquini@ufu.br (D.P.)

3 *Trivandrum Engineering Science & Technology Research Park, Mahatma Gandhi University, Kottayam, 686560, Kerala, India;* sabuthomas@mgu.ac.in (S.T.)

4 *State Key Laboratory of Chemical Engineering, School of Chemical Engineering and Technology, Tianjin University, Tianjin 300072, China;* surx@tju.edu.cn (R.S.)

* Correspondence: m.dmitrienko@spbu.ru (M.D.); a.penkova@spbu.ru (A.P.); Tel.: +7-(812)-363-60-00 (ext. 3367) (M.D. & A.P.)

S1. Film Characterization

Surface chemical analysis of films was conducted via X-ray photoelectron spectroscopy (XPS) on an ESCALAB 250Xi instrument equipped with a monochromatic Al Kα source (hν= 1486.6 eV). Wide-scan survey spectra were acquired at a constant pass energy of 100 eV, whereas high-resolution core-level regions were collected at 50 eV, using an X-ray spot diameter of 650 μm. Surface charge neutralization was accomplished via a dual electron–ion flood gun system. The overall spectral resolution for core-level scans was approximately 0.8 eV, with a binding energy determination accuracy of ±0.2 eV and a relative uncertainty in elemental quantification of ~10%. The component ratios for C1s, O1s and N1s were calculated from Table S1. Table S2 presents the elemental composition and component ratios for films.

Table S1: C1s, O1s, N1s XPS spectra for films.

Film	C1s	O1s	N1s
T2308	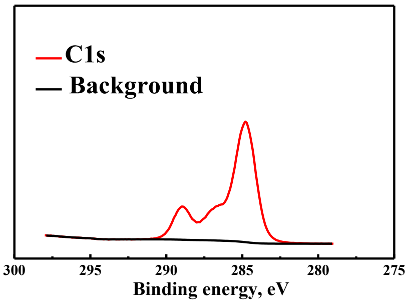	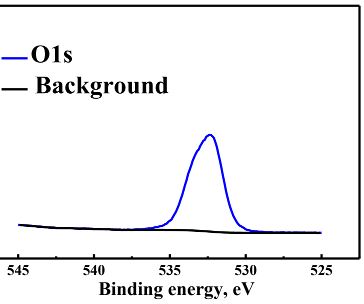	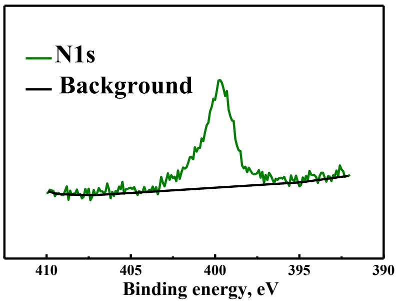

F2332	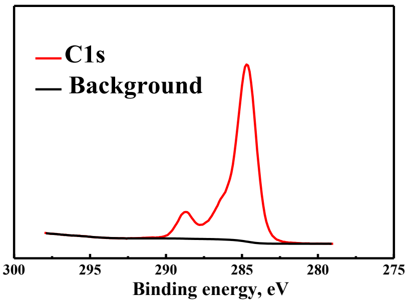	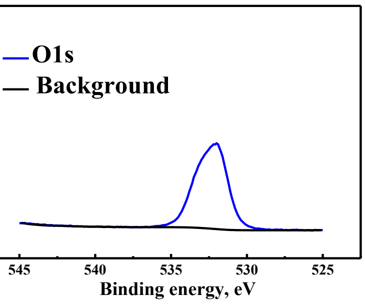	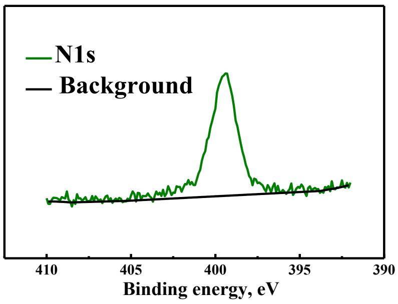
T2308+Curcumin	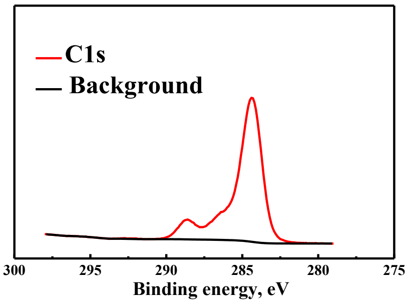	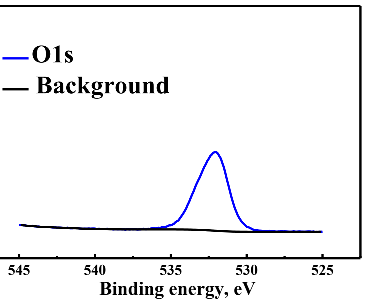	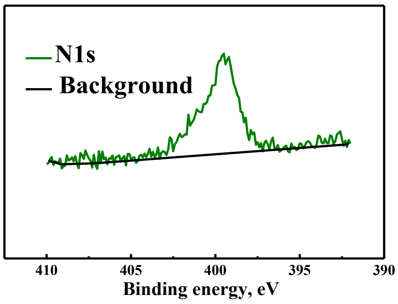
F2332+ Curcu-min	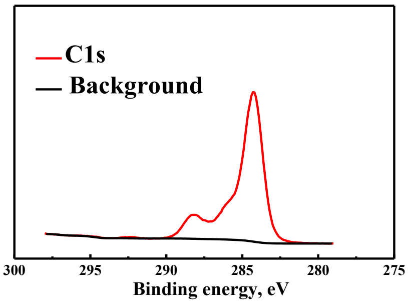	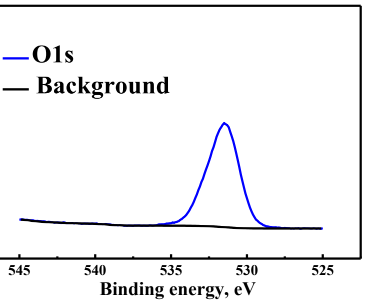	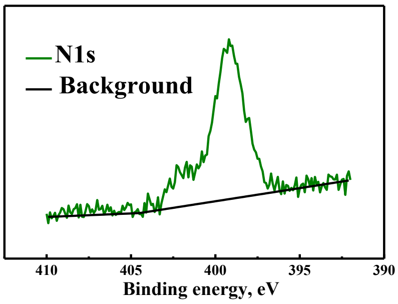
T2308+Nisin	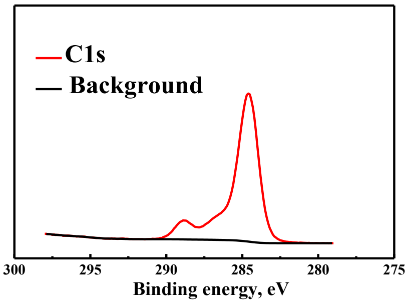	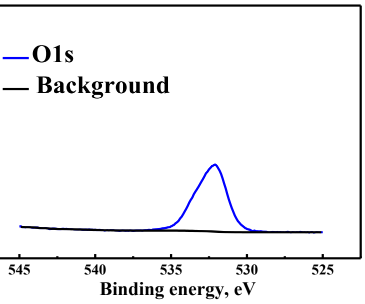	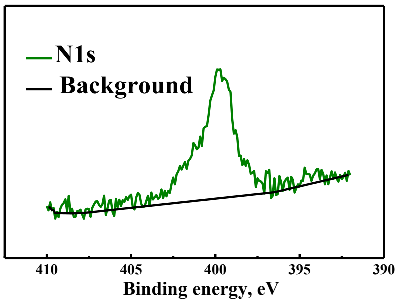
F2332+Nisin	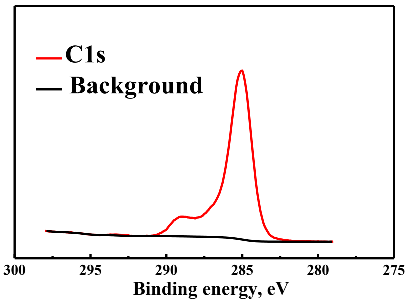	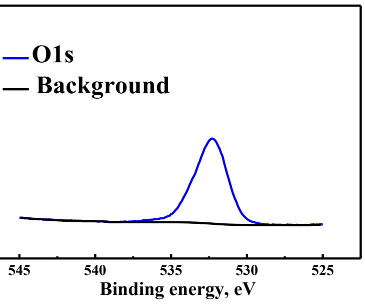	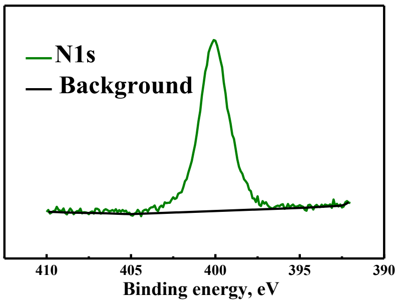

T2308+ZnO	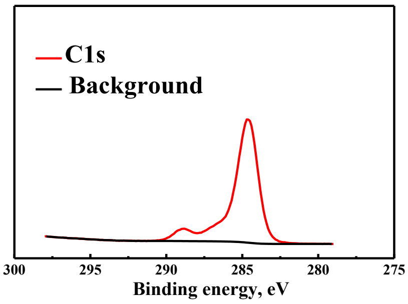	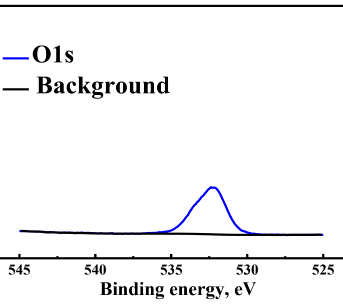	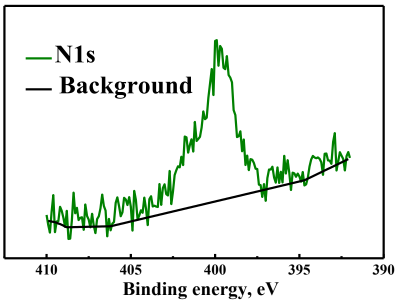
F2332+ZnO	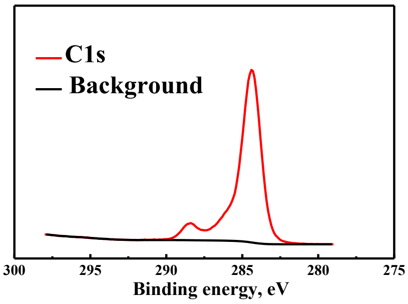	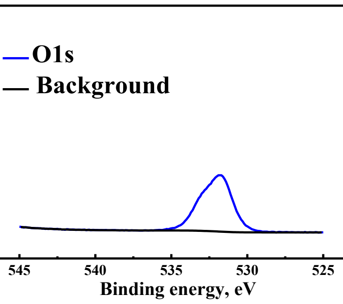	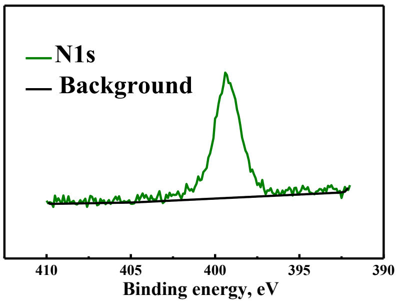
T2308+EL oil	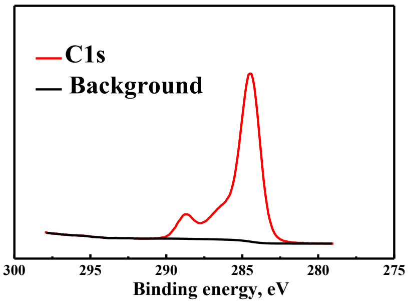	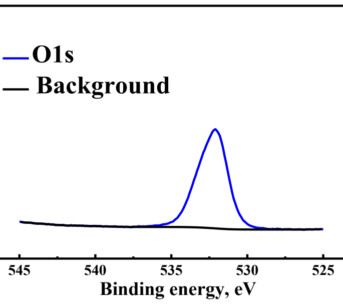	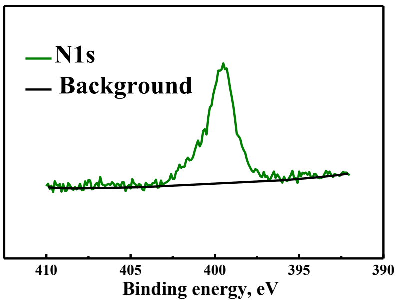
F2332+EL oil	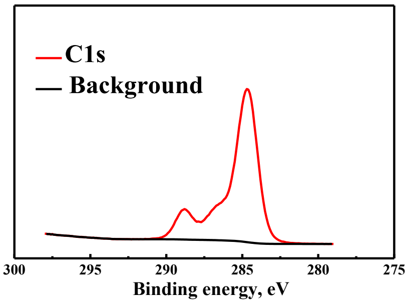	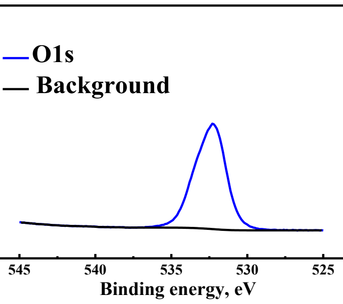	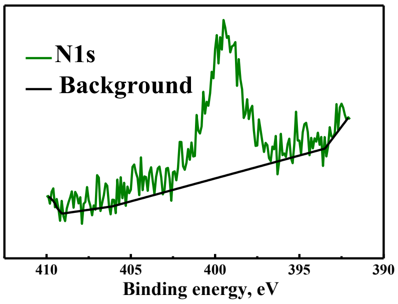
T2308+LG oil	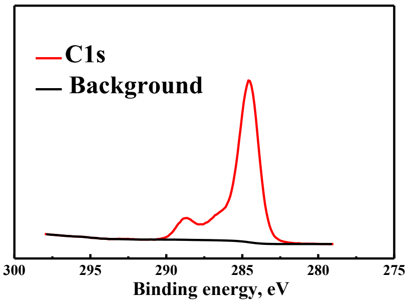	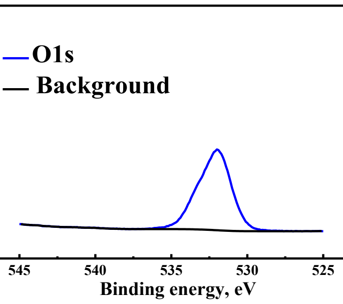	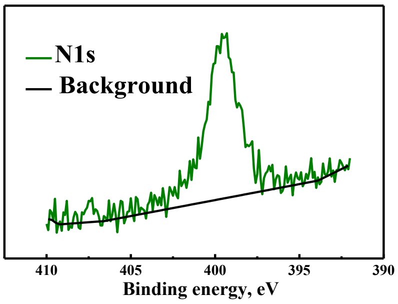

**F2332+LG oil**	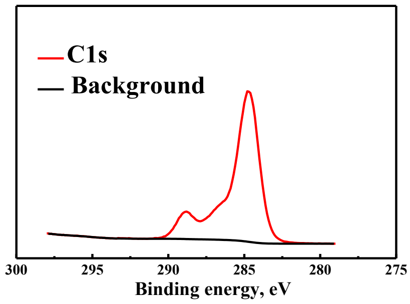	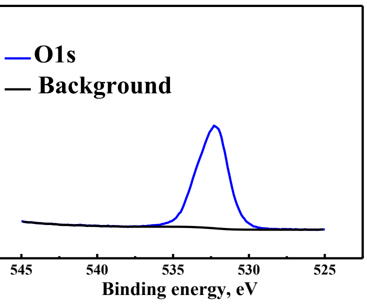	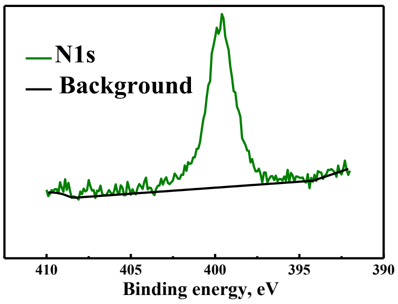
**T2308+TT oil**	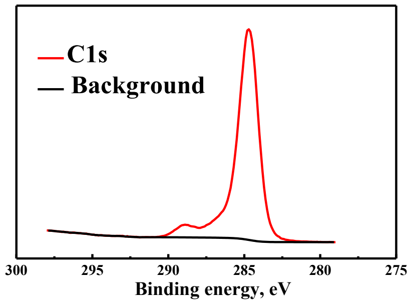	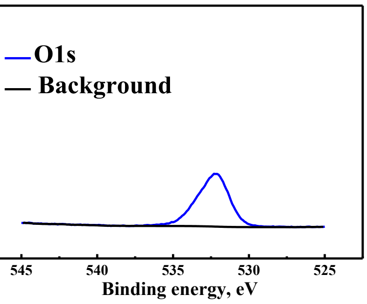	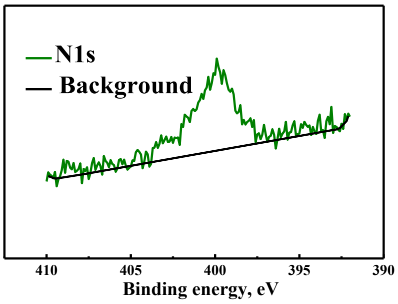
**F2332+TT oil**	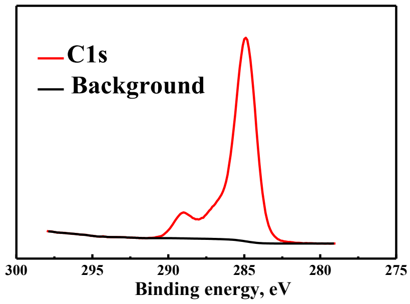	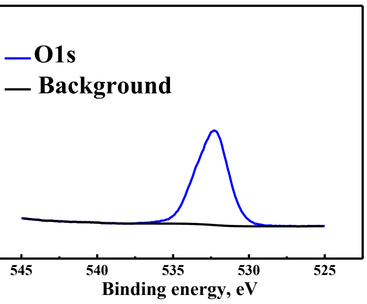	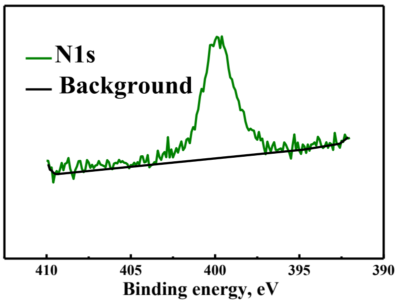
**T2308+CL oil**	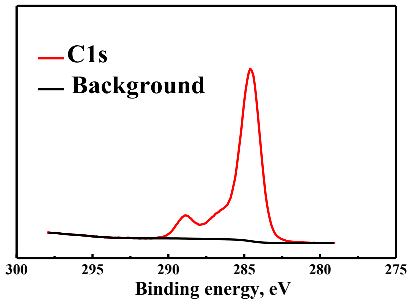	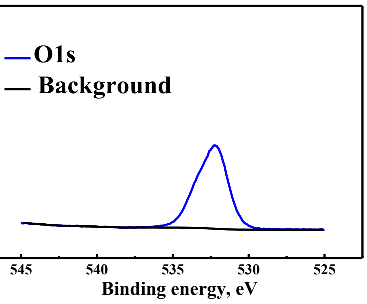	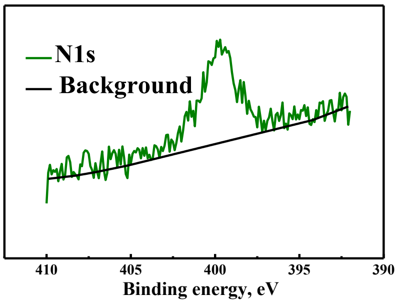
**F2332+CL oil**	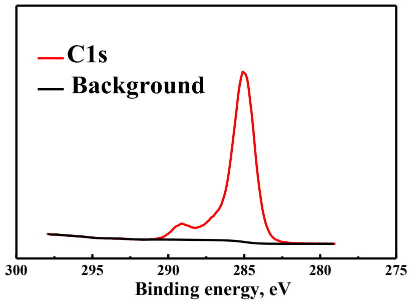	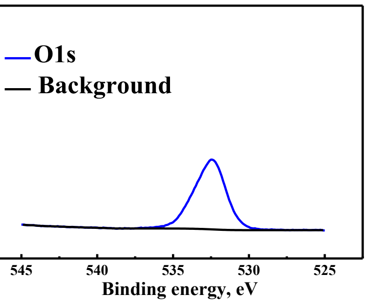	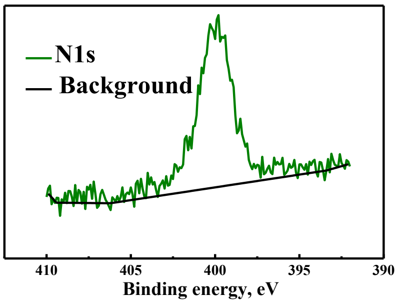

Table S2: The elemental composition and component ratios for films.

film \ element	C1s	O1s	N1s	Cl2p	S2p	Zn2p	P2p
**T2308**	73.21	25.32	0.79	0.5	0.17	-	-
**F2332**	79.14	19.65	1.1	-	0.11	-	-
**T2308+Curcumin**	77.48	20.56	0.87	0.79	0.3	-	-
**F2332+ Curcumin**	72.71	25	1.04	0.55	0.15	-	0.55
**T2308+Nisin**	79.78	17.85	0.89	1.14	0.27	0.07	-
**F2332+Nisin**	76.41	19.38	2.51	0.98	0.65	0.08	-
**T2308+ZnO**	81.57	16.26	0.72	1.12	0.23	0.1	-
**F2332+ZnO**	83.14	15.37	1.32	-	0.11	0.07	-
**T2308+EL oil**	77.02	20.94	1.09	0.58	0.27	0.11	-
**F2332+EL oil**	76.3	23.1	0.47	-	0.13	-	-
**T2308+LG oil**	78.87	20.03	0.65	0.07	0.31	0.06	-
**F2332+LG oil**	75.49	23.01	1.11	0.17	0.16	0.06	-
**T2308+TT oil**	85	12.38	0.62	1.7	0.3	-	-
**F2332+TT oil**	80.07	78.76	0.75	0.08	0.23	0.12	-
**T2308+CL oil**	78.89	19.16	0.48	1.25	0.23	-	-
**F2332+CL oil**	81.71	17.01	0.9	0.14	0.24	-	-

XPS surface analysis of the film formulations confirmed the dominant presence of carbon (C1s, 72.7–85.0 at.%) and oxygen (O1s, 12.4–25.3 at.%) originating from the polymer matrix backbone, alongside minor amounts of nitrogen, chlorine, and sulfur (Table S2). The incorporation of bioactive additives introduced distinct surface chemical signatures: nisin addition substantially enriched surface nitrogen (N1s up to 2.51 at.% for F2332+Nisin) and sulfur (S2p = 0.65 at.%), confirming the surface localization of the peptide structure. ZnO embedding was verified by characteristic Zn2p peaks (0.07–0.10 at.%), where low surface atomic fractions indicate that inorganic nanoparticles are mostly encapsulated within the bulk polymer matrix. Lipophilic essential oils (most notably TT oil, T2308+TT oil, with C1s = 85.00 at.%) significantly increased surface carbon levels owing to their hydrocarbon-rich terpenoid constituents. And curcumin preserved the organic carbon-oxygen balance with trace elements (P2p = 0.55 at.% in F2332+Curcumin). Overall, these surface elemental trends validate the successful integration and characteristic physical distribution of all functional active agents within both T2308 and F2332 film matrices.

The morphological features and surface elemental distribution of the membrane samples were evaluated using a Zeiss Merlin field-emission scanning electron microscope (FE-SEM) integrated with an INCA X-Act energy-dispersive X-ray (EDX) detector (Oxford Instruments). Quantitative elemental composition and EDX mapping were performed under an accelerating voltage of 15 kV and a beam current of 2 nA. For high-resolution structural imaging, the operating conditions were adjusted to 2 kV and a reduced probe current of 100 pA. To prevent electrostatic charge accumulation during analysis, all specimens were sputter-coated with a ~15 nm carbon layer prior to measurement. SEM combined with EDX spectroscopy was utilized to investigate the internal morphology (cross-section, Table S3) and surface characteristics (Table S4) of the developed films, along with the spatial distribution of embedded active agents.

Table S3: SEM image with EDX of cross-sectional of films.

Film	SEM image with EDX	C Kα1_2	O Kα1	Zn Lα1_2
**T2308**	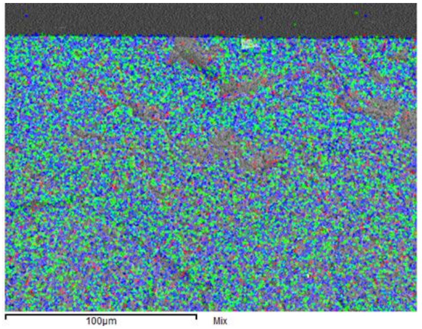	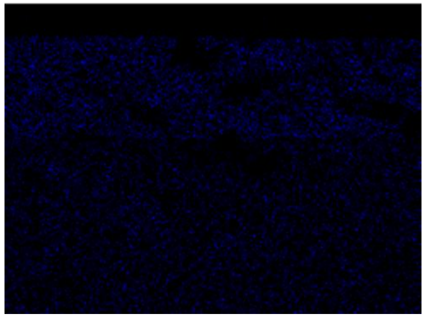	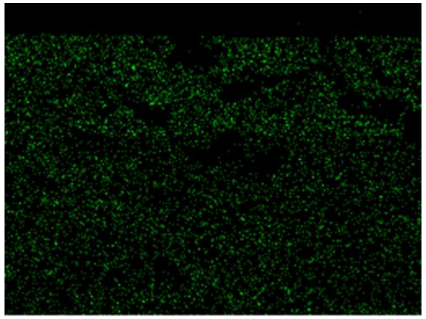	-
**F2332**	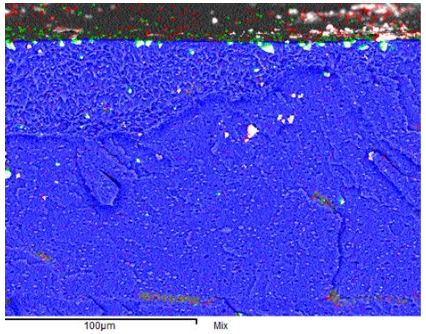	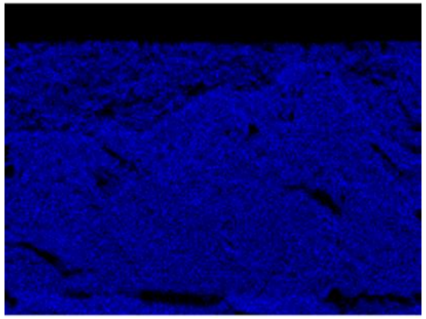	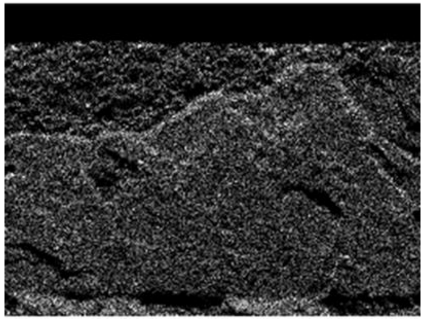	-

T2308+Cur-cumin	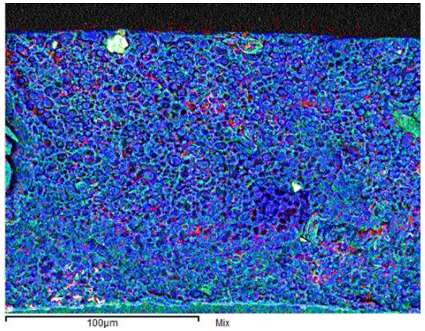	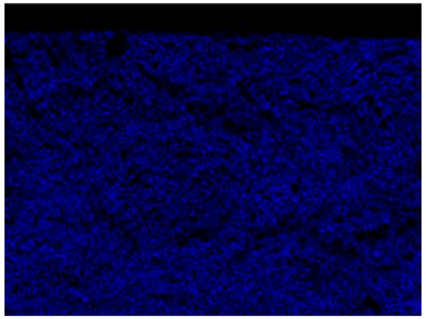	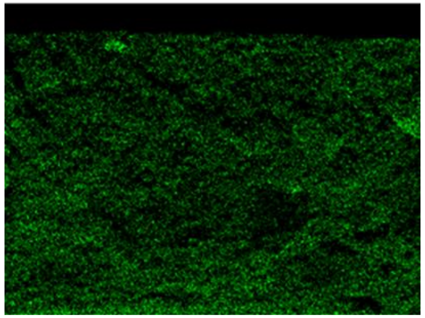	-
F2332+ Cur-cumin	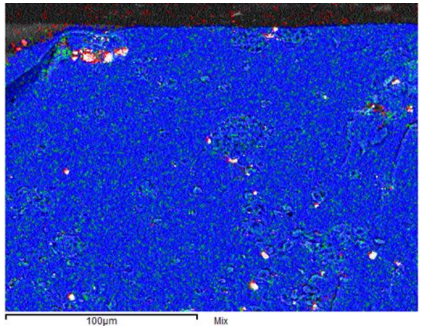	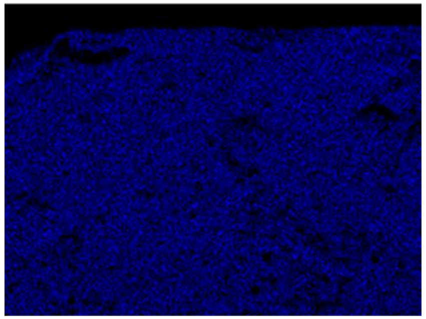	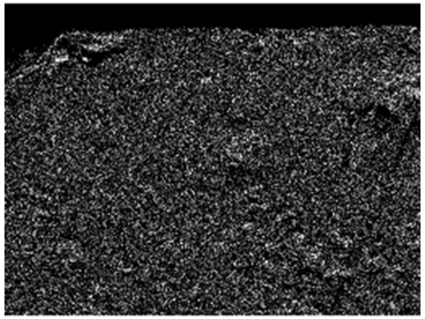	-
T2308+Nisin	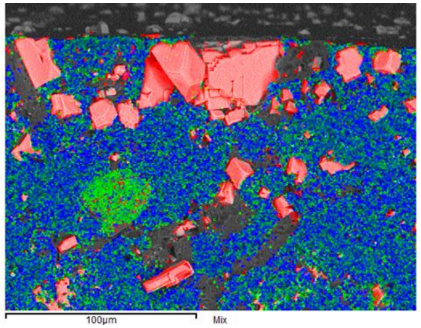	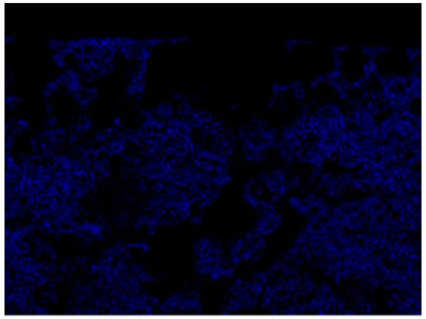	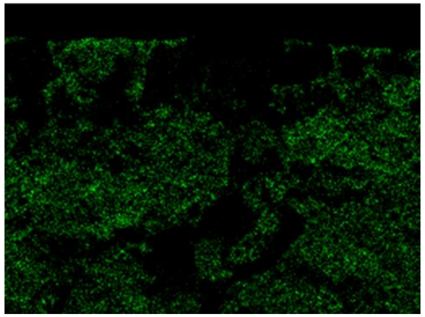	-

**F2332+Nisin**	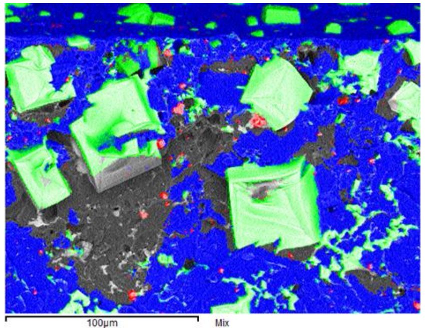	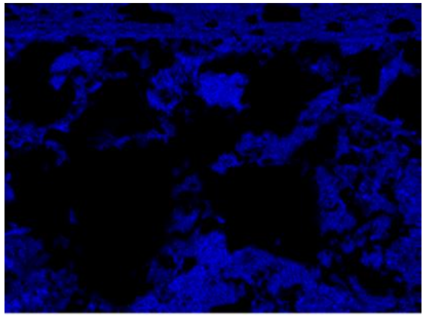	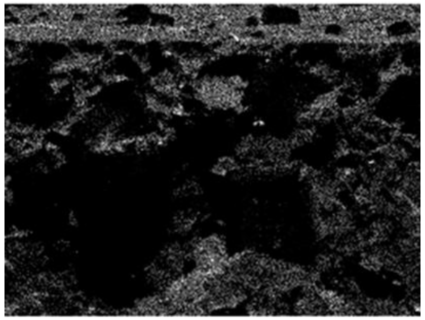	-
**T2308+ZnO**	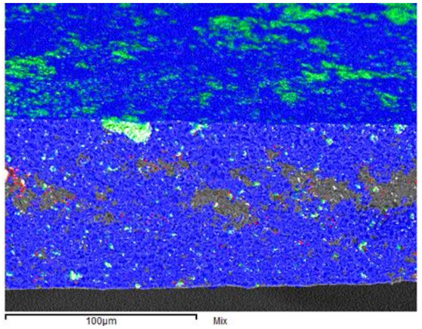	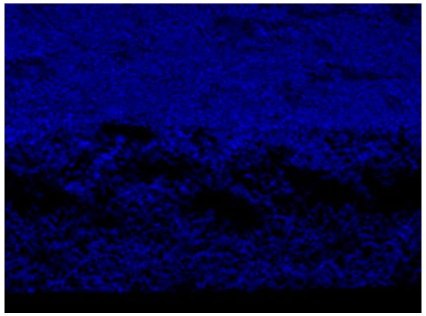	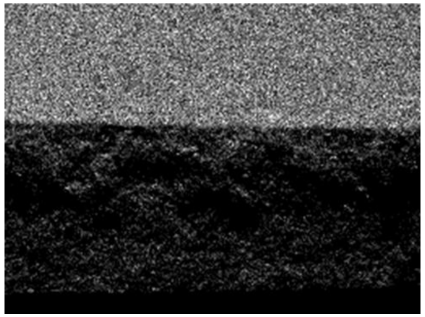	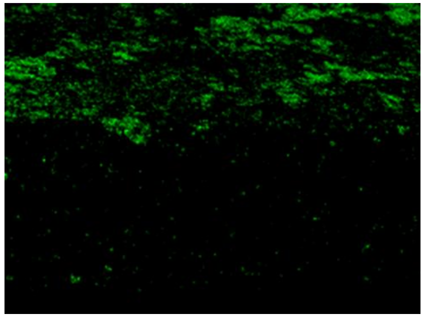
**F2332+ZnO**	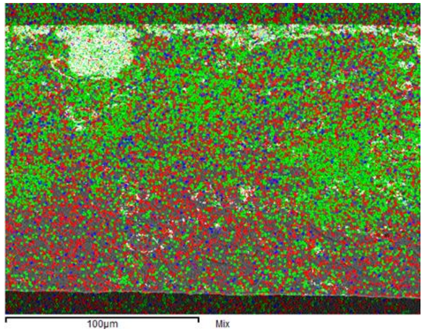	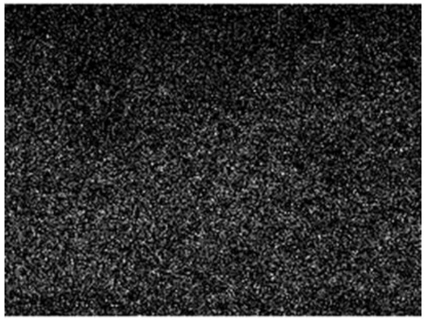	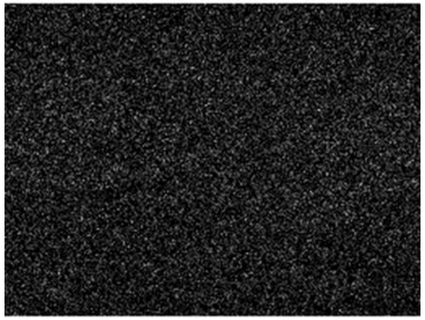	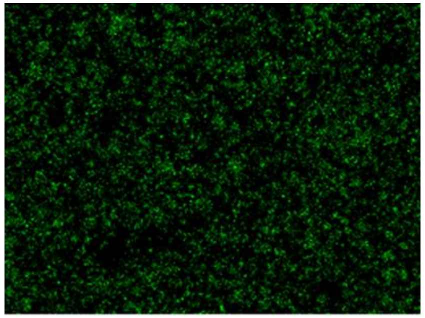

**T2308+EL oil**	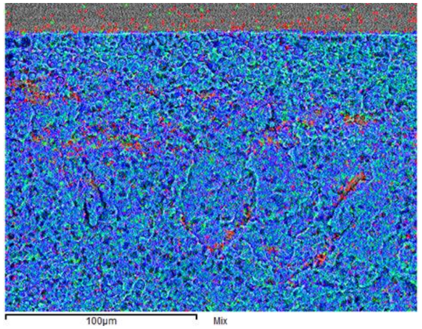	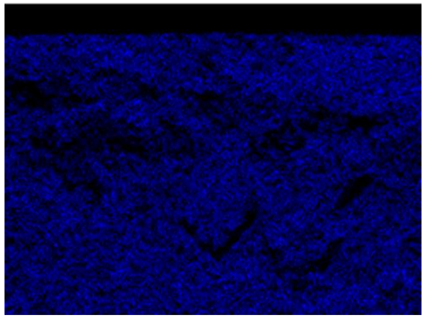	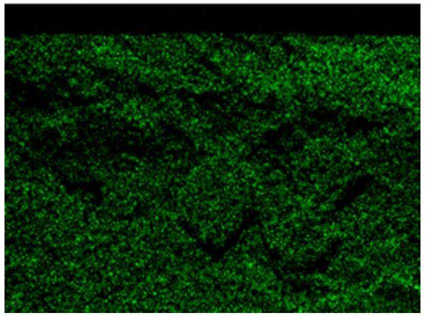	-
**F2332+EL oil**	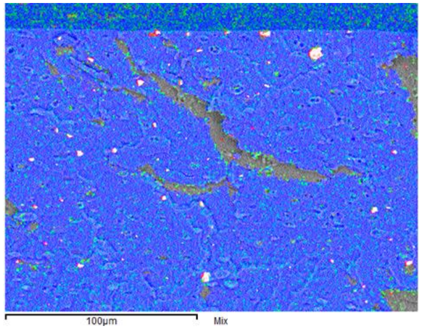	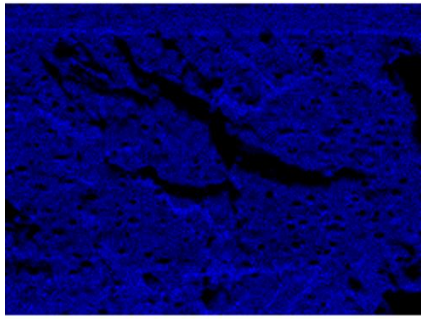	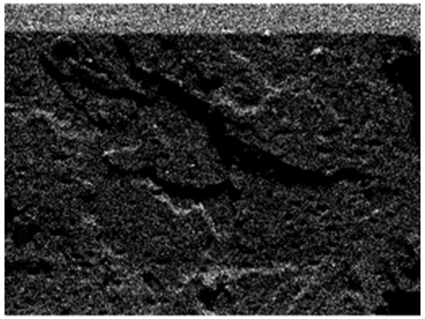	-
**T2308+LG oil**	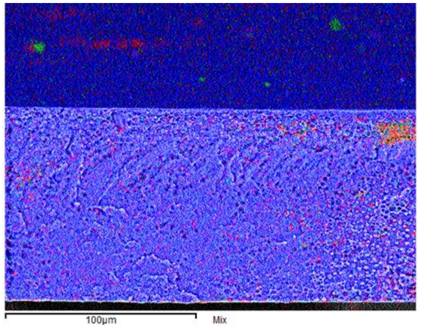	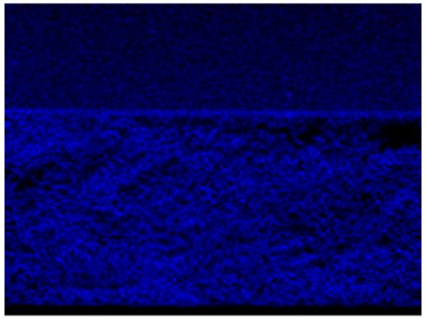	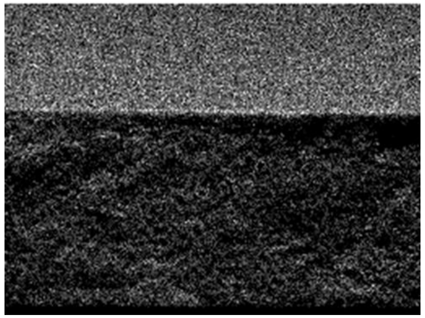	-

**F2332+LG oil**	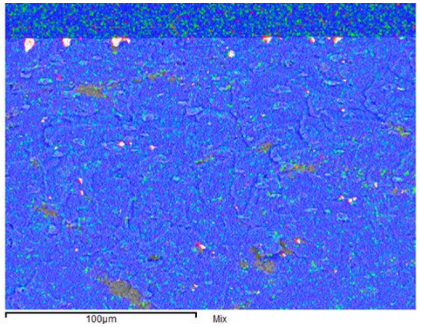	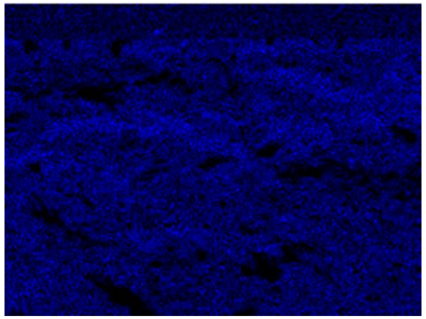	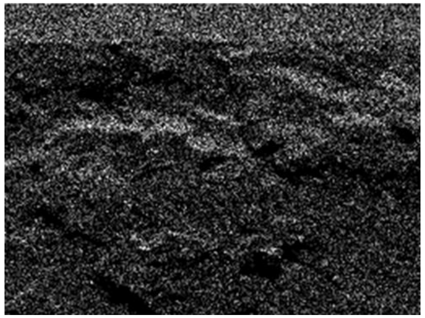	-
**T2308+TT oil**	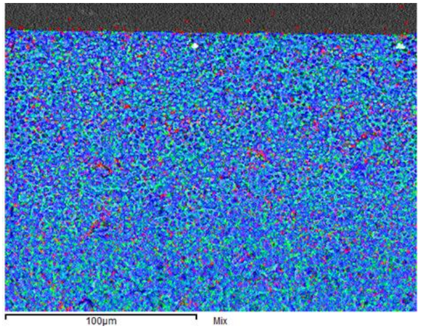	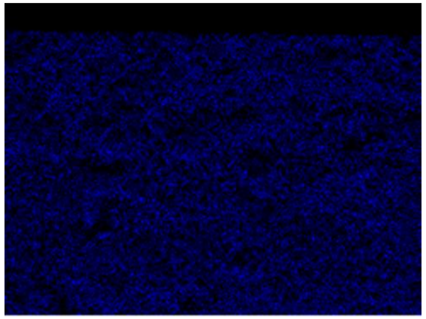	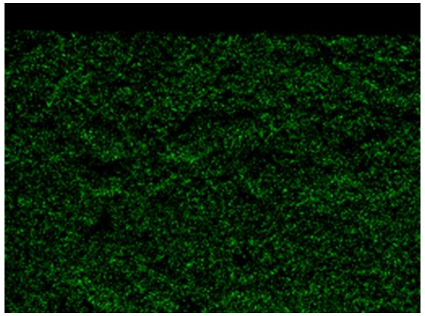	-
**F2332+TT oil**	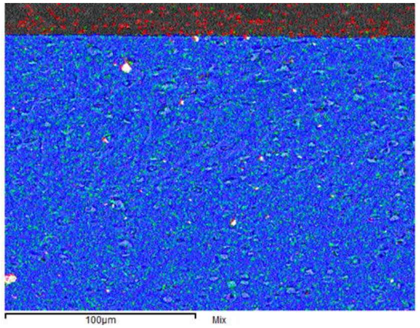	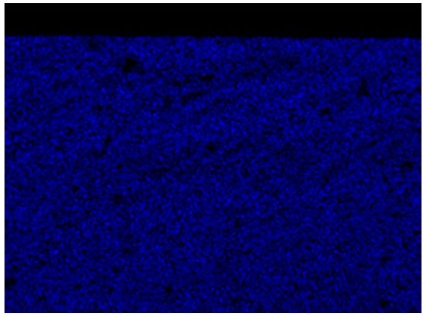	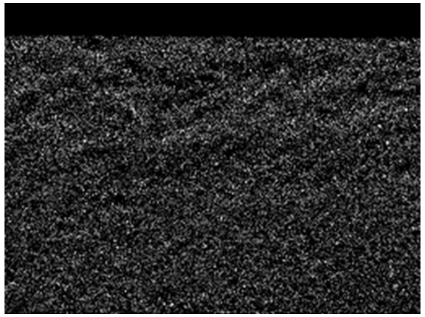	-

**T2308+CL oil**	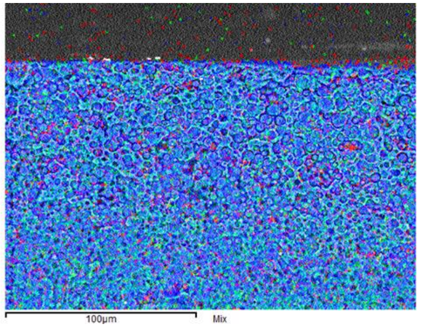	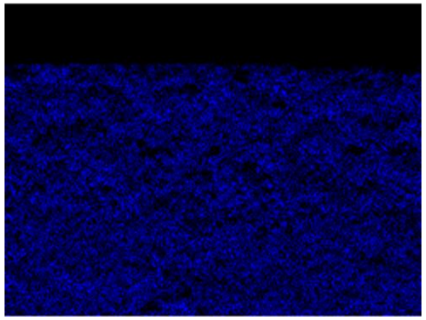	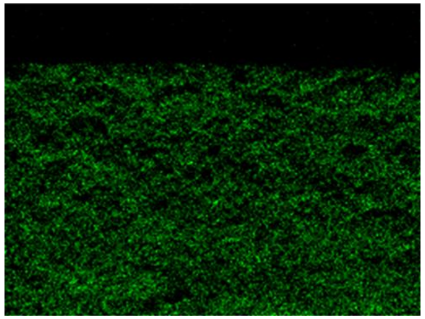	-
**F2332+CL oil**	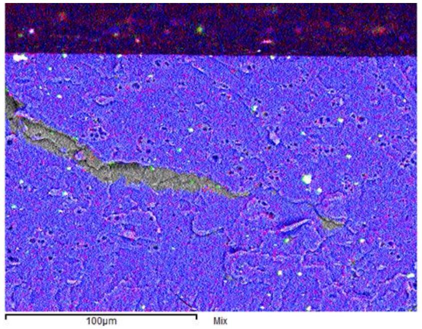	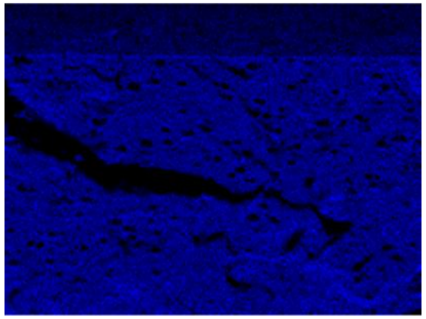	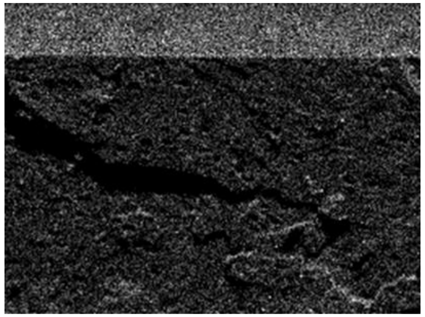	-

Table S4: SEM image with EDX of surface of films.

Film	SEM image with EDX	C Kα1_2	O Kα1	Zn Lα1_2
**T2308**	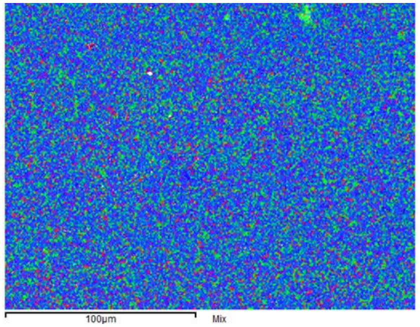	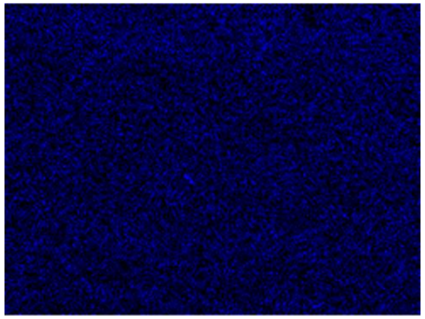	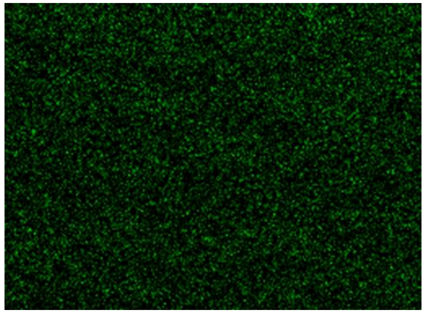	-

**F2332**	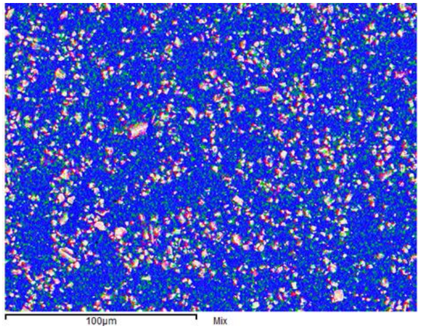	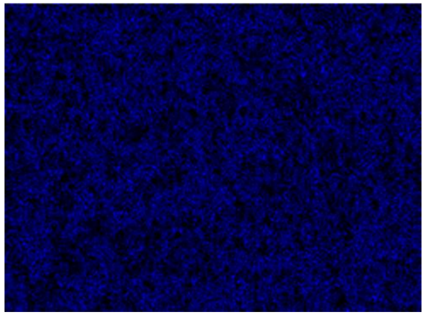	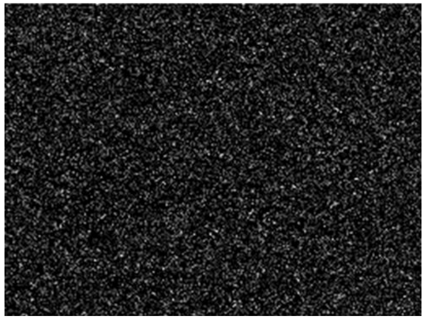	-
**T2308+Cur-cumin**	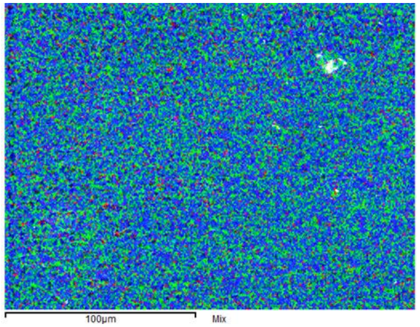	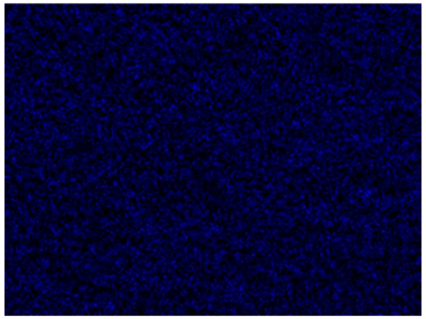	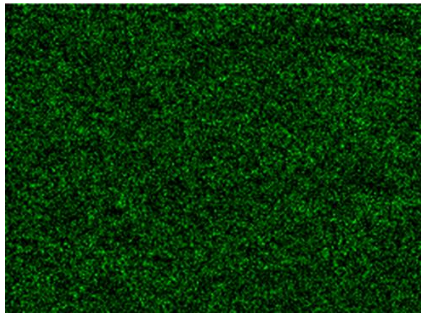	-
**F2332+Cur-cumin**	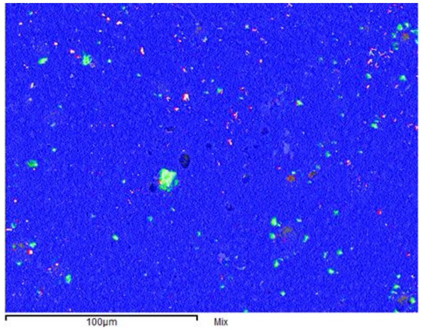	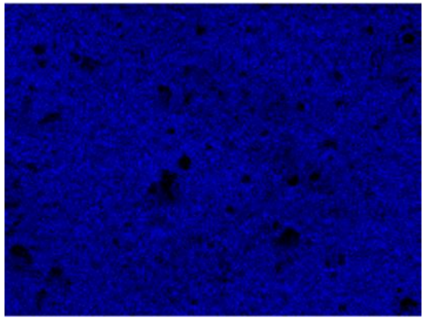	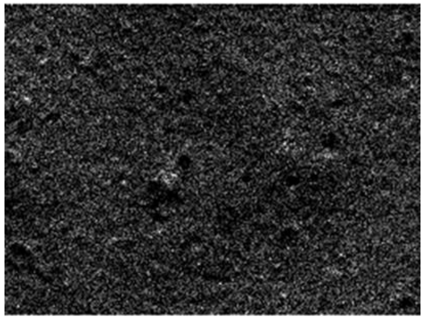	-

**T2308+Nisin**	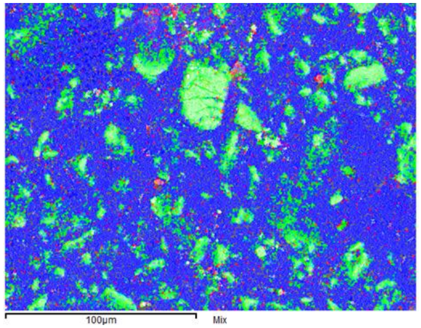	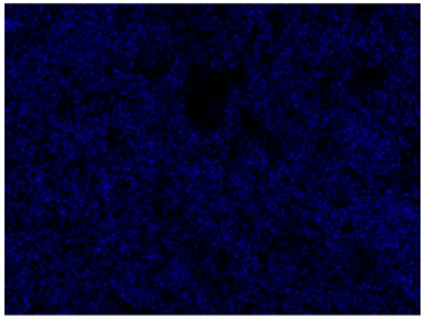	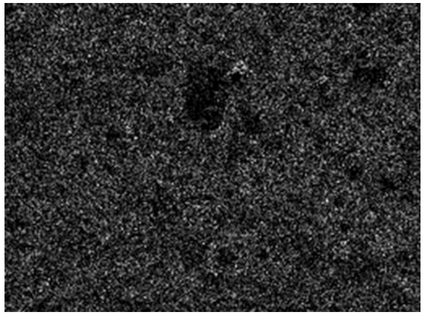	-
**F2332+Nisin**	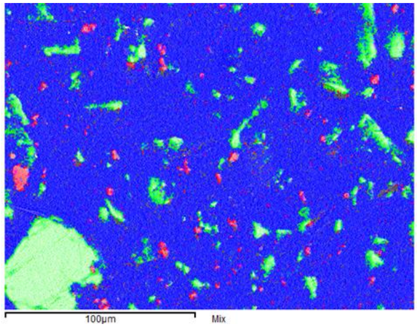	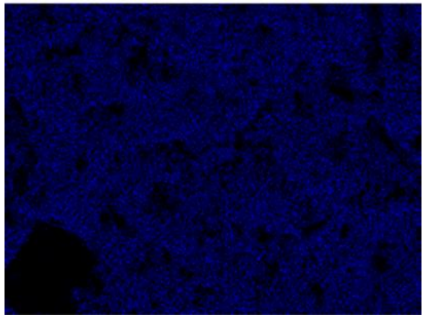	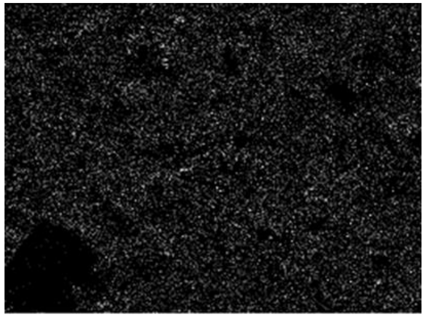	-
**T2308+ZnO**	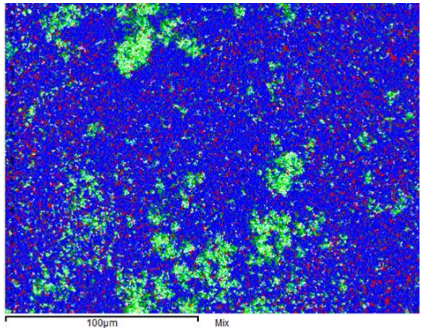	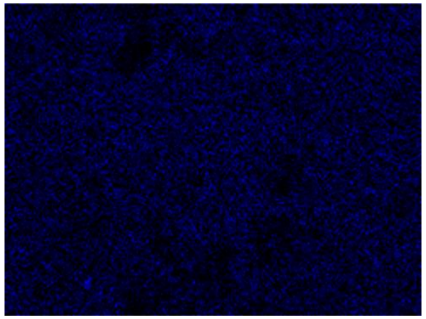	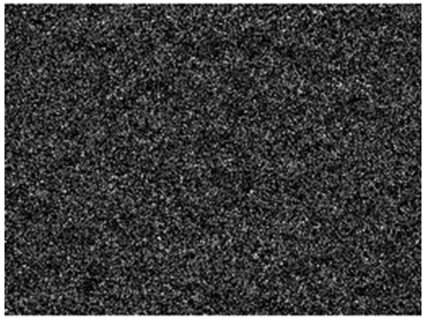	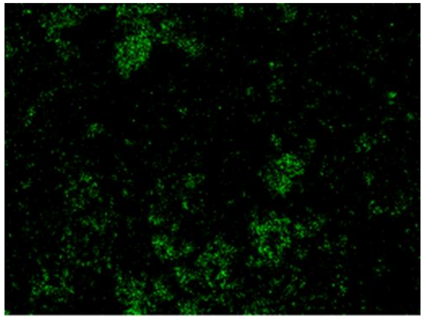

**F2332+ZnO**	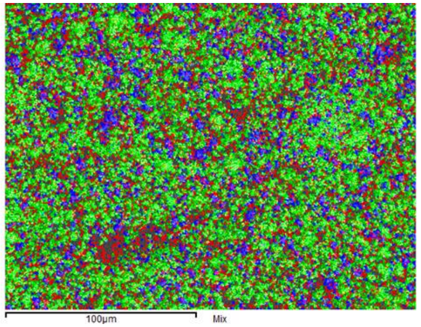	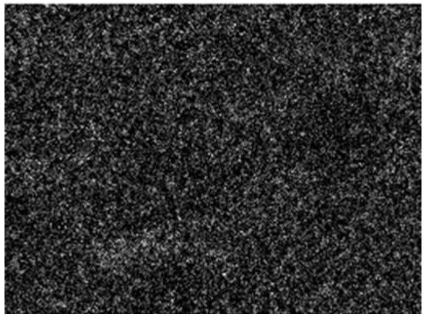	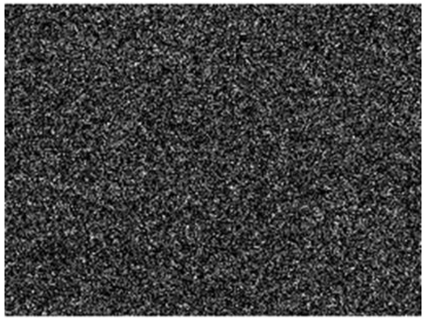	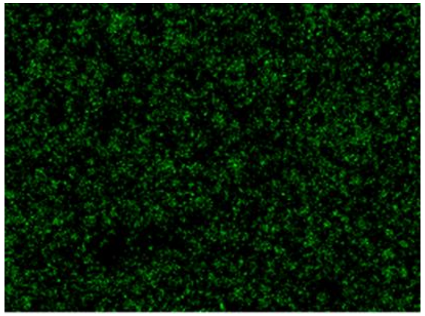
**T2308+EL oil**	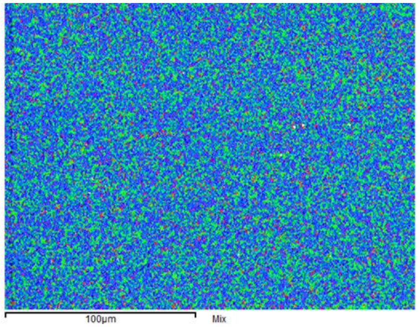	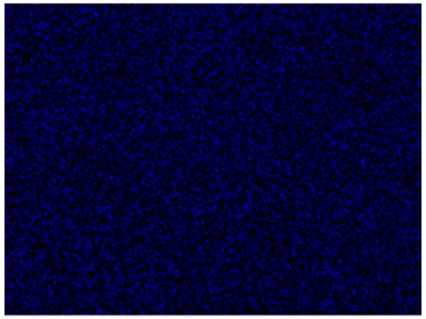	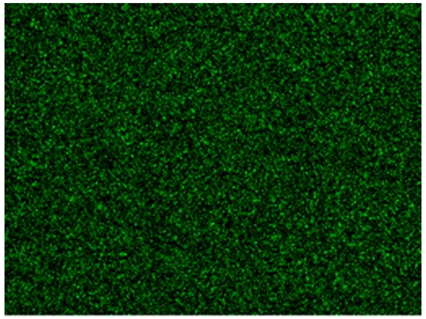	-
**F2332+EL oil**	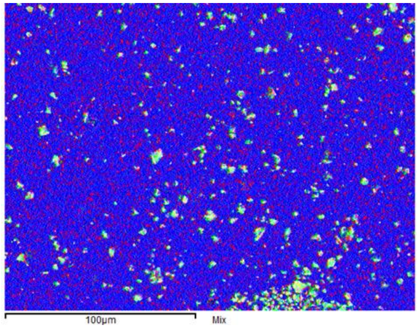	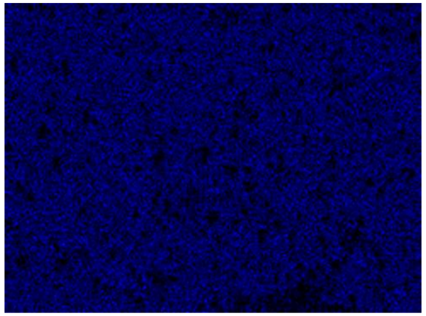	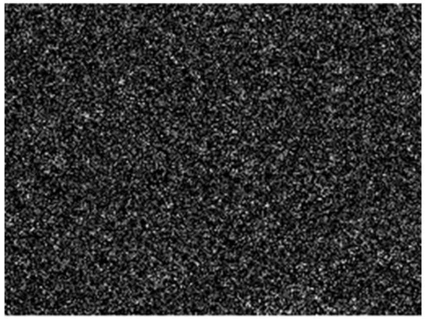	-

**T2308+LG oil**	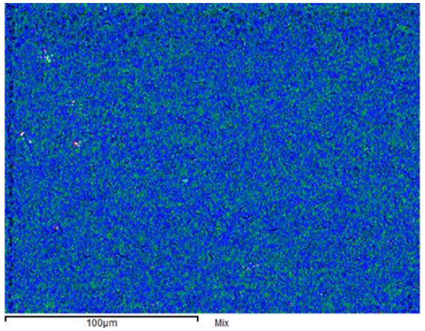	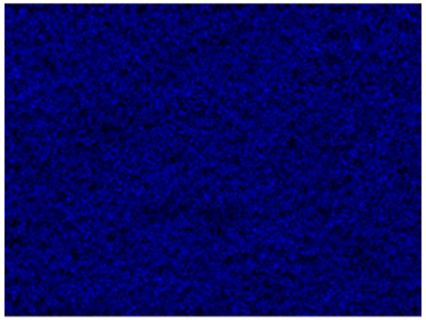	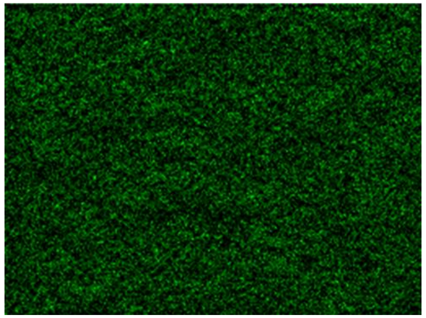	-
**F2332+LG oil**	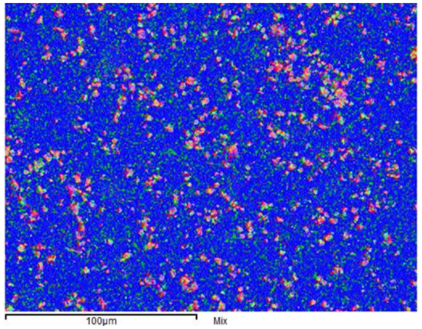	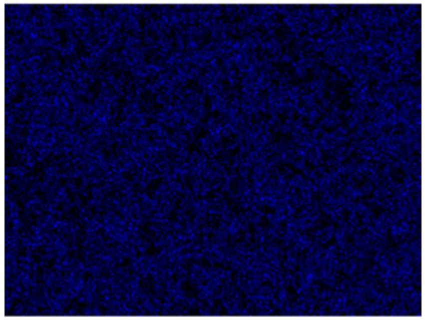	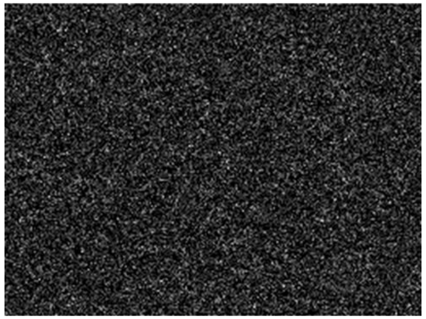	-
**T2308+TT oil**	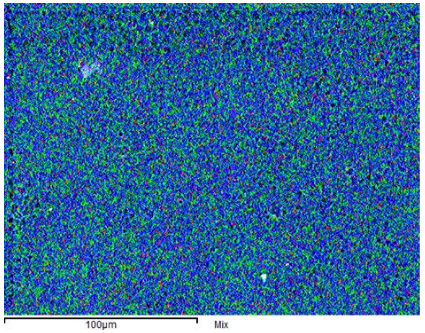	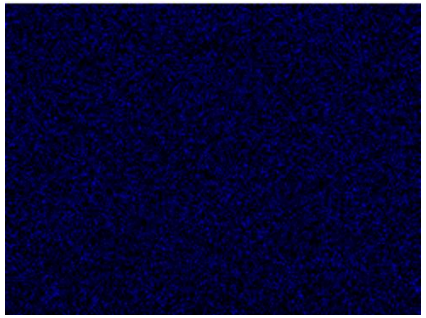	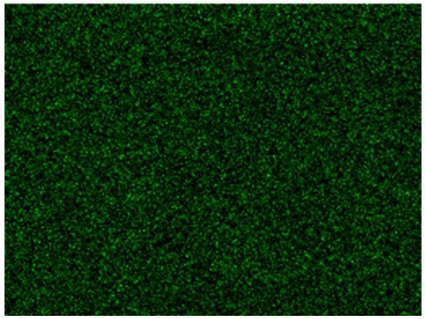	-

**F2332+TT oil**	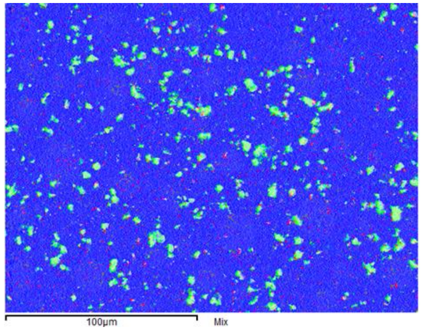	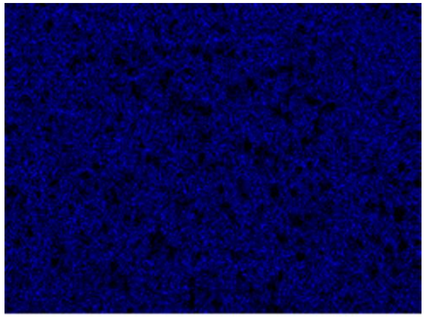	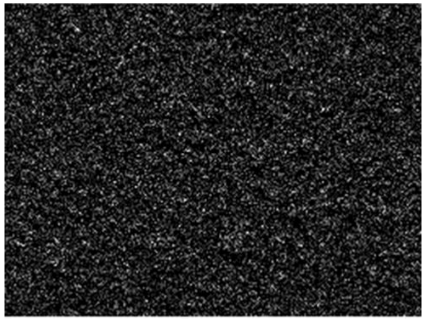	-
**T2308+CL oil**	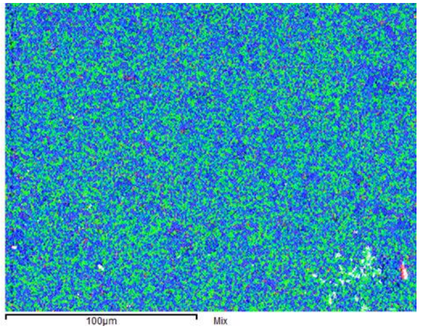	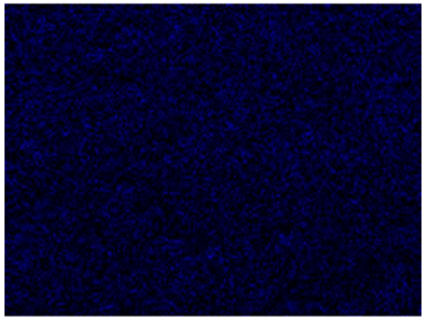	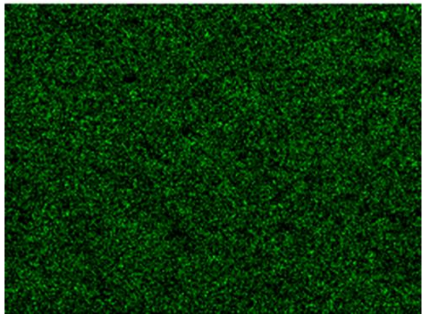	-
**F2332+CL oil**	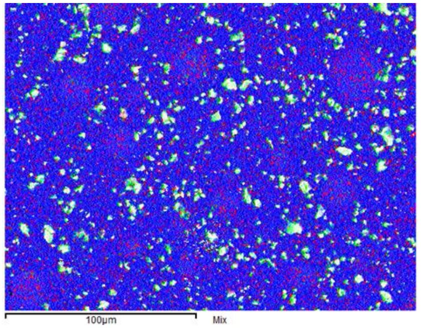	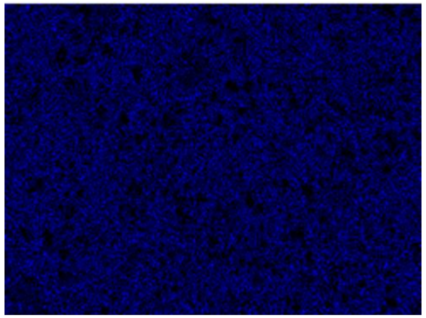	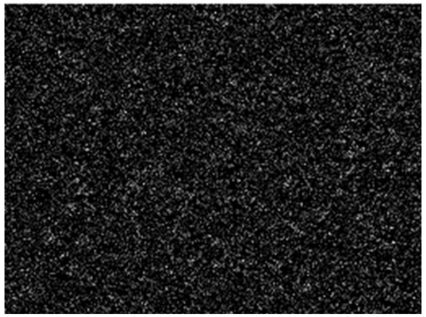	-

Pristine PLA/PBAT films (T2308 and F2332) exhibited a dense, homogeneous structure without visible phase separation or particulate matter, characterized by uniform carbon and oxygen elemental maps. Upon incorporation of active additives, distinct morphological changes and elemental localizations were observed: nisin-modified films (T2308+Nisin, F2332+Nisin) displayed discrete, micron-sized aggregates in both cross-sectional and surface SEM/EDX overlays, indicating the formation of distinct nisin-rich domains. For ZnO-modified films (T2308+ZnO, F2332+ZnO), SEM images clearly revealed embedded nanoparticles, confirmed by pronounced zinc elemental maps (Zn Lα1_2) distributed throughout the cross-section and on the film surface, albeit with some degree of particle aggregation. Conversely, films incorporating curcumin (T2308+Curcumin, F2332+Curcumin) and various essential oils generally maintained a highly homogeneous morphology in both cross-section and surface views, with EDX maps primarily showing uniform carbon and oxygen distribution. This suggests that these additives are either finely dispersed at a molecular or nanoscale level, or effectively integrated into the polymer matrix without forming large, distinguishable aggregates.

Maximum elongation and tensile strength of films are presented in Table S5. The load applied for all specimens ranged from 0.10 to 2.50 N, approaching the lower limit of the testing instrument. The mechanical behavior of the PLA/PBLAT blends is strongly governed by matrix–additive interactions, with a clear, matrix-specific ductility–strength trade-off. In T2308, TT and LG oils markedly increase elongation (to about 66 and 53 mm) while preserving high strength (≈35 and 33 MPa). Curcumin boosts elongation modestly (~11–12 mm) but drastically reduces strength. ZnO and nisin yield modest elongation (≈3–5 mm) with higher strengths (≈14–22 MPa), and EL and CL oils provide intermediate improvements. In F2332, LG oil drives an exceptionally large elongation (~167 mm) with reduced strength (~16 MPa), while CL also gives large elongation (~117 mm) with ~16 MPa. TT oil yields ~84 mm elongation and ~11 MPa, ZnO - ~45 mm with ~12 MPa, and nisin - ~38 mm with ~9 MPa. Curcumin substantially lowers both properties. Overall, the data indicate that achieving higher ductility often compromises strength, and the optimal additive and loading are strongly dependent on the specific PLA/PBLAT formulation. For applications prioritizing ductility, TT/LG and LG/CL oils are promising in T2308 and F2332, respectively. For higher strength, ZnO is preferred, albeit with reduced elongation, while curcumin generally diminishes both properties, especially in F2332.

Table S5: Maximum elongation and tensile strength.

Sample	Maximum elongation, mm	Tensile strength, MPa
**T2308**	3.8	33.0
**T2308+15%Curcumin**	11.5	3.85
**T2308+30%Nisin**	3.4	14.6
**T2308+10%ZnO**	4.6	21.8
**T2308+5%EL**	9.2	24.3
**T2308+5%LG**	66.1	33.5
**T2308+5%TT**	52.8	35.1
**T2308+5%CL**	23.4	31.9
**F2332**	182.1	18.2
**F2332+15% Curcumin**	49.8	8.0
**F2332+30%Nisin**	38.4	9.1
**F2332+10%ZnO**	44.9	11.8
**F2332+5%EL**	34.1	8.6
**F2332+5%LG**	166.8	15.7
**F2332+5%TT**	83.6	11.4
**F2332+5%CL**	116.9	16.1

DSC was used to examine the thermal properties and polymer–additive compatibility of the formulations; the findings (for the first heating) for films with curcumin, nisin, and oils are presented in Figure S1.

the first heating

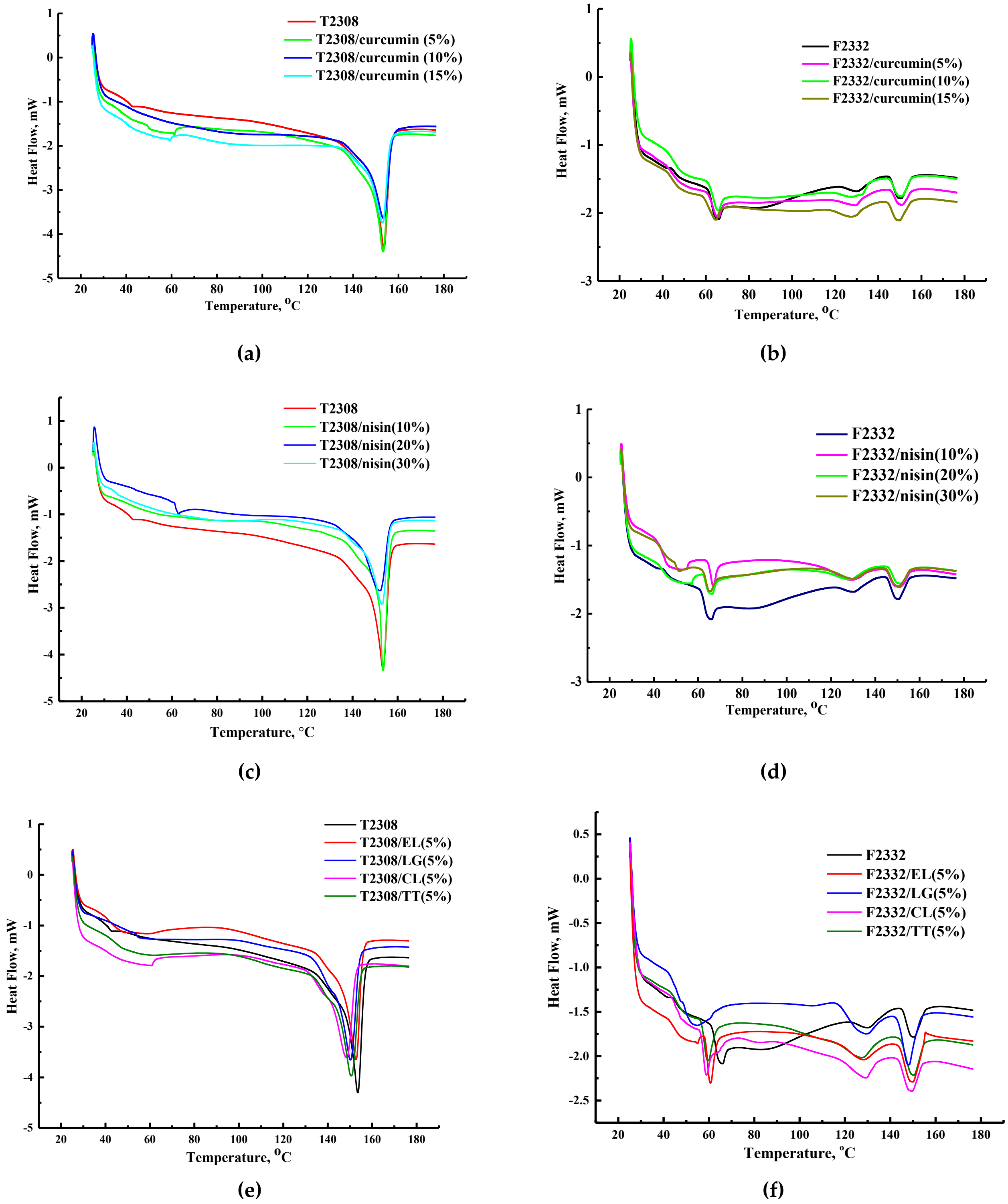

Figure S1: DSC curves for the first heating scan for the films (**a, c, e**) from T2308 and (**b, d, f**) F2332 with additives.

To demonstrate the pH-response of curcumin-modified films, they were immersed in HCl (1M), NaOH (1M) and buffer solutions of different pH levels: potassium tetraoxalate ($KH_3C_4O8 \cdot 2H_2O$) 0.05M (pH = 1.68), saturated potassium tartrate acid ($KC_4H_5O_6$) (pH= 3.56), potassium phthalate acid ($KC_8H_5O_4$) (pH = 4.01), potassium phosphate (KH_2PO_4) and sodium phosphate (Na_2HPO_4) (pH= 6.86), sodiumtetraborate ($Na_2B_4O_7 \cdot 10H_2O$) (pH = 9.18), and saturated calciumoxyhydrate ($Ca(OH)_2$) (pH= 12.45). The color of the films and solutions was assessed every 60 min for up to 24 h

(Table S6). Significant color changes were observed in weakly alkaline and alkaline media due to tautomeric transitions and deprotonation (Figure S2).

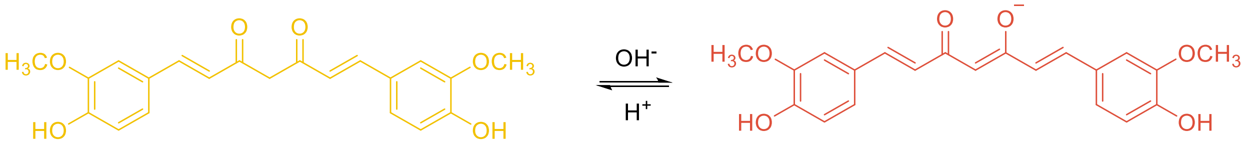

Figure S2: Keto-enol tautomers of curcumin

Table S6: Photos of the resulting solutions before and after 24 h, showing the color change depending on pH (from left to right: solutions with pH 0.01, 1.68, 3.56, 4.01, 6.86, 9.18, 12.45, 13.80)

**T2308+15%Curcumin**	
**before**	**After 24h**
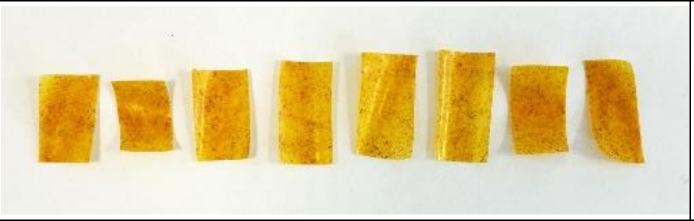	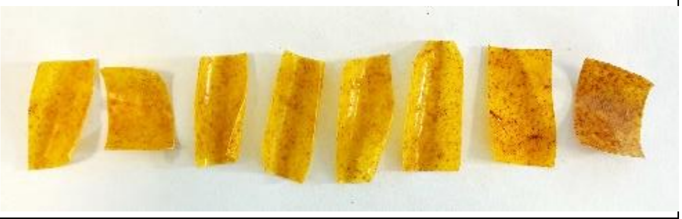
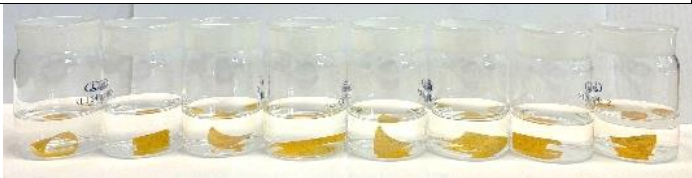	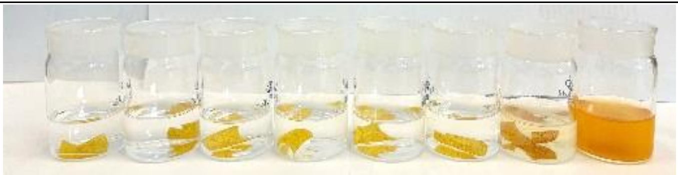
**F2332+15%Curcumin**	
**before**	**After 24h**
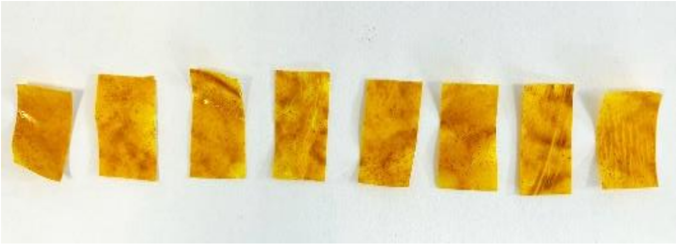	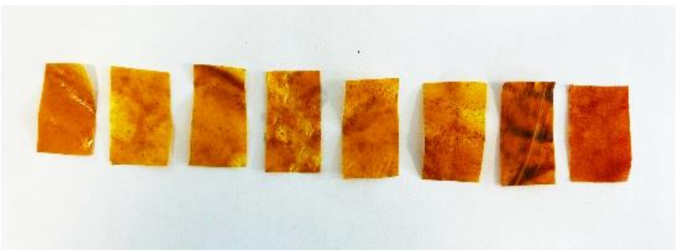
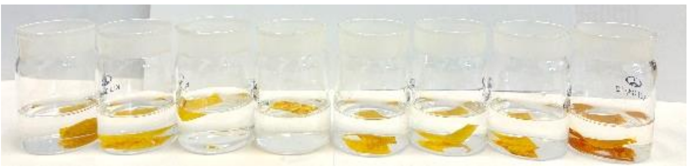	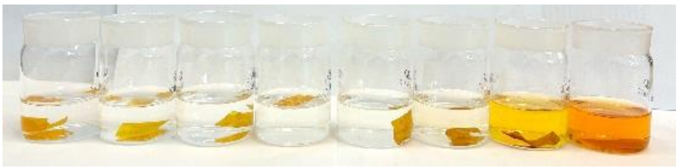

The color change of the indicator is clearly visible under mildly to strongly alkaline conditions. Therefore, the pH-indicating properties of curcumin can be effectively utilized to monitor the quality of foods that undergo an alkaline pH shift during spoilage. During the spoilage of various meat products, enzymes and microbial activity degrade proteins, leading to the accumulation of volatile amines and other nitrogenous compounds. This accumulation typically elevates the pH of the meat product [46]. Demonstrating this application, researchers [47] observed a distinct color transition from yellow to orange in gum/polyvinyl alcohol films incorporating curcumin, confirming its potential as a reliable colorimetric indicator.

S2. ANOVA and Tukey Test Results

Interpretation: groups sharing at least one lowercase letter are not significantly different by Tukey's test at $\alpha = 0.05$; groups with no letter in common are significantly different.

Origin convention: Sig = 1 indicates a significant mean difference at $\alpha = 0.05$; Sig = 0 indicates no significant difference.

S2.1 WVP data

1. T2308 – Curcumin

Overall ANOVA

Source	DF	Sum of Squares	Mean Square	F Value	Prob > F
Model	3	10.060154	3.353385	25.970509	1.037187e-07
Error	24	3.098947	0.129123	—	—
Total	27	13.159101	—	—	—

Tukey Test

Comparison	MeanDiff	SEM	q Value	Prob	Alpha	Sig	LCL	UCL
0.05 0	1.642104	0.192073	12.090612	1.204647e-07	0.05	1	1.112249	2.17196
0.1 0	0.931026	0.192073	6.855031	0.000336	0.05	1	0.40117	1.460882
0.1 0.05	-0.711078	0.192073	5.235581	0.005732	0.05	1	-1.240934	-0.181222
0.15 0	1.186037	0.192073	8.732645	1.256213e-05	0.05	1	0.656181	1.715893
0.15 0.05	-0.456067	0.192073	3.357967	0.109414	0.05	0	-0.985923	0.073788
0.15 0.1	0.255011	0.192073	1.877614	0.554992	0.05	0	-0.274845	0.784867

Tukey grouping (lowercase letters)

Level	Tukey group
0%	c
0.05	a
0.10	b
0.15	ab

2. F2332 – Curcumin

Overall ANOVA

Source	DF	Sum of Squares	Mean Square	F Value	Prob > F
Model	3	63.452516	21.150839	1145.952999	0
Error	24	0.442968	0.018457	—	—
Total	27	63.895484	—	—	—

Tukey Test

Comparison	MeanDiff	SEM	q Value	Prob	Alpha	Sig	LCL	UCL
0.05 0	4.046825	0.072618	78.81032	0	0.05	1	3.8465	4.247151
0.1 0	3.024288	0.072618	58.896812	2.362962e-08	0.05	1	2.823963	3.224614
0.1 0.05	-1.022537	0.072618	19.913508	0	0.05	1	-1.222863	-0.822212
0.15 0	2.884185	0.072618	56.168354	2.245302e-08	0.05	1	2.683859	3.08451
0.15 0.05	-1.162641	0.072618	22.641967	0	0.05	1	-1.362966	-0.962315
0.15 0.1	-0.140103	0.072618	2.728459	0.242779	0.05	0	-0.340429	0.060222

Tukey grouping (lowercase letters)

Level	Tukey group
0%	c
0.05	a
0.10	b
0.15	b

3. T2308 – Nisin

Overall ANOVA

Source	DF	Sum of Squares	Mean Square	F Value	Prob > F
Model	3	1.500548	0.500183	102.605561	7.982504e-14
Error	24	0.116995	0.004875	—	—
Total	27	1.617543	—	—	—

Tukey Test

Comparison	MeanDiff	SEM	q Value	Prob	Alpha	Sig	LCL	UCL
0.1 0	-0.454838	0.03732	17.235605	0	0.05	1	-0.55779	-0.351886
0.2 0	-0.491527	0.03732	18.625927	0	0.05	1	-0.594479	-0.388575
0.2 0.1	-0.03669	0.03732	1.390322	0.760274	0.05	0	-0.139642	0.066262
0.3 0	-0.608538	0.03732	23.059915	0	0.05	1	-0.71149	-0.505586
0.3 0.1	-0.1537	0.03732	5.82431	0.002069	0.05	1	-0.256652	-0.050748
0.3 0.2	-0.11701	0.03732	4.433988	0.02176	0.05	1	-0.219962	-0.014058

Tukey grouping (lowercase letters)

Level	Tukey group
0%	a
0.10	b
0.20	b
0.30	c

4. F2332 – Nisin

Overall ANOVA

Source	DF	Sum of Squares	Mean Square	F Value	Prob > F
Model	3	0.013405	0.004468	0.07807	0.971261
Error	24	1.373594	0.057233	—	—
Total	27	1.386999	—	—	—

Tukey Test

Comparison	MeanDiff	SEM	q Value	Prob	Alpha	Sig	LCL	UCL
0.1 0	-0.04245	0.127876	0.469466	0.987066	0.05	0	-0.39521	0.31031
0.2 0	-0.028859	0.127876	0.319153	0.995834	0.05	0	-0.381619	0.323902
0.2 0.1	0.013592	0.127876	0.150312	0.999557	0.05	0	-0.339169	0.366352
0.3 0	-0.059827	0.127876	0.661636	0.965384	0.05	0	-0.412587	0.292934
0.3 0.1	-0.017376	0.127876	0.19217	0.999078	0.05	0	-0.370137	0.335384
0.3 0.2	-0.030968	0.127876	0.342483	0.994869	0.05	0	-0.383728	0.321792

Tukey grouping (lowercase letters)

Level	Tukey group
0%	a
0.10	a
0.20	a
0.30	a

5. F 2332 ZnO

Overall ANOVA

Source	DF	Sum of Squares	Mean Square	F Value	Prob > F
Model	3	0.475152	0.158384	2.960903	0.056929
Error	20	1.069835	0.053492	—	—
Total	23	1.544987	—	—	—

Tukey Test

Comparison	MeanDiff	SEM	q Value	Prob	Alpha	Sig	LCL	UCL
0.05 0	0.138196	0.133531	1.463623	0.731372	0.05	0	-0.235549	0.511942
0.07 0	0.232124	0.133531	2.458397	0.331068	0.05	0	-0.141621	0.605869
0.07 0.05	0.093927	0.133531	0.994774	0.894536	0.05	0	-0.279818	0.467673
0.1 0	0.386561	0.133531	4.094026	0.041031	0.05	1	0.012816	0.760307
0.1 0.05	0.248365	0.133531	2.630403	0.276192	0.05	0	-0.12538	0.62211
0.1 0.07	0.154437	0.133531	1.635629	0.660001	0.05	0	-0.219308	0.528183

Tukey grouping (lowercase letters)

Level	Tukey group
0%	b
0.05	ab
0.07	ab
0.10	a

6. T2308 – ZnO

Overall ANOVA

Source	DF	Sum of Squares	Mean Square	F Value	Prob > F
Model	3	2.061985	0.687328	8.5462	0.000749
Error	20	1.6085	0.080425	—	—
Total	23	3.670486	—	—	—

Tukey Test

Comparison	MeanDiff	SEM	q Value	Prob	Alpha	Sig	LCL	UCL
0.05 0	0.023181	0.163733	0.200226	0.998952	0.05	0	-0.435095	0.481458
0.07 0	0.355898	0.163733	3.074011	0.164843	0.05	0	-0.102379	0.814175
0.07 0.05	0.332716	0.163733	2.873785	0.209787	0.05	0	-0.12556	0.790993
0.1 0	0.720064	0.163733	6.219441	0.00146	0.05	1	0.261788	1.178341
0.1 0.05	0.696883	0.163733	6.019215	0.002017	0.05	1	0.238606	1.15516
0.1 0.07	0.364167	0.163733	3.14543	0.150809	0.05	0	-0.09411	0.822443

Tukey grouping (lowercase letters)

Level	Tukey group
0%	b
0.05	b
0.07	ab
0.10	a

7. T2308 – CL / TT / EL / LG (5%)

Overall ANOVA

Source	DF	Sum of Squares	Mean Square	F Value	Prob > F
Model	4	18.27923	4.569807	6.527689	0.000665
Error	30	21.001953	0.700065	—	—
Total	34	39.281183	—	—	—

Tukey Test

Comparison	MeanDiff	SEM	q Value	Prob	Alpha	Sig	LCL	UCL
CL 5% 0%	-0.534894	0.447234	1.691406	0.753564	0.05	0	-1.832146	0.762358
TT 5% 0%	-0.376937	0.447234	1.191923	0.914955	0.05	0	-1.674189	0.920315
TT 5% CL 5%	0.157958	0.447234	0.499482	0.996478	0.05	0	-1.139294	1.45521
EL 5% 0%	1.468797	0.447234	4.644529	0.020323	0.05	1	0.171545	2.766049
EL 5% CL 5%	2.003692	0.447234	6.335934	0.000892	0.05	1	0.70644	3.300944
EL 5% TT 5%	1.845734	0.447234	5.836452	0.002324	0.05	1	0.548482	3.142986
LG 5% 0%	-0.22012	0.447234	0.696049	0.987501	0.05	0	-1.517372	1.077132
LG 5% CL 5%	0.314774	0.447234	0.995356	0.954021	0.05	0	-0.982478	1.612026
LG 5% TT 5%	0.156816	0.447234	0.495874	0.996575	0.05	0	-1.140436	1.454068
LG 5% EL 5%	-1.688918	0.447234	5.340578	0.005872	0.05	1	-2.98617	-0.391666

Tukey grouping (lowercase letters)

Level	Tukey group
0%	b
CL 5%	b
TT 5%	b
EL 5%	a
LG 5%	b

8. F2332 – CL / TT / EL / LG (5%)

Overall ANOVA

Source	DF	Sum of Squares	Mean Square	F Value	Prob > F
Model	4	4.585327	1.146332	13.970497	1.513945e-06
Error	30	2.461612	0.082054	—	—
Total	34	7.046939	—	—	—

Tukey Test

Comparison	MeanDiff	SEM	q Value	Prob	Alpha	Sig	LCL	UCL
CL 5% 0%	-0.338762	0.153114	3.12892	0.202715	0.05	0	-0.782886	0.105362
TT 5% 0%	0.486192	0.153114	4.490637	0.026419	0.05	1	0.042068	0.930317
TT 5% CL 5%	0.824954	0.153114	7.619558	7.210329e-05	0.05	1	0.38083	1.269079
EL 5% 0%	0.68171	0.153114	6.2965	0.000963	0.05	1	0.237585	1.125834
EL 5% CL 5%	1.020472	0.153114	9.42542	2.083597e-06	0.05	1	0.576347	1.464596
EL 5% TT 5%	0.195517	0.153114	1.805862	0.706918	0.05	0	-0.248607	0.639641
LG 5% 0%	0.089789	0.153114	0.829323	0.976086	0.05	0	-0.354335	0.533913
LG 5% CL 5%	0.428551	0.153114	3.958243	0.062689	0.05	0	-0.015573	0.872675
LG 5% TT 5%	-0.396403	0.153114	3.661315	0.098063	0.05	0	-0.840527	0.047721
LG 5% EL 5%	-0.59192	0.153114	5.467177	0.004647	0.05	1	-1.036045	-0.147796

Tukey grouping (lowercase letters)

Level	Tukey group
0%	c
CL 5%	c
TT 5%	ab
EL 5%	a
LG 5%	bc

S2.2 MA data

1. T2308 – Curcumin

Overall ANOVA

Source	DF	Sum of Squares	Mean Square	F Value	Prob > F
Model	3	0.161662	0.053887	1.435733	0.26531
Error	18	0.675592	0.037533	—	—
Total	21	0.837254	—	—	—

Tukey Test

Comparison	MeanDiff	SEM	q Value	Prob	Alpha	Sig	LCL	UCL
0.05 0	-0.068278	0.125055	0.772144	0.946437	0.05	0	-0.42172	0.285163
0.1 0	-0.065497	0.111852	0.828118	0.935149	0.05	0	-0.381625	0.25063
0.1 0.05	0.002781	0.125055	0.031453	0.999996	0.05	0	-0.35066	0.356222
0.15 0	-0.223923	0.111852	2.831183	0.223906	0.05	0	-0.54005	0.092205
0.15 0.05	-0.155644	0.125055	1.760143	0.607864	0.05	0	-0.509086	0.197797
0.15 0.1	-0.158426	0.111852	2.003065	0.505751	0.05	0	-0.474553	0.157702

Tukey grouping (lowercase letters)

Group / formulation	Letter
0	a
0.1	a
0.05	a
0.15	a

2. F2332 – Curcumin

Overall ANOVA

Source	DF	Sum of Squares	Mean Square	F Value	Prob > F
Model	3	0.255742	0.085247	1.418092	0.266899
Error	20	1.202283	0.060114	—	—
Total	23	1.458025	—	—	—

Tukey Test

Comparison	MeanDiff	SEM	q Value	Prob	Alpha	Sig	LCL	UCL
0.05 0	-0.178319	0.141556	1.781495	0.597826	0.05	0	-0.574524	0.217887
0.1 0	-0.239172	0.141556	2.38945	0.35485	0.05	0	-0.635378	0.157033
0.1 0.05	-0.060853	0.141556	0.607955	0.97265	0.05	0	-0.457059	0.335352
0.15 0	-0.264183	0.141556	2.639319	0.273526	0.05	0	-0.660388	0.132023
0.15 0.05	-0.085864	0.141556	0.857824	0.928793	0.05	0	-0.48207	0.310342
0.15 0.1	-0.025011	0.141556	0.249869	0.997973	0.05	0	-0.421216	0.371195

Tukey grouping (lowercase letters)

Group / formulation	Letter
0	a
0.05	a
0.1	a
0.15	a

3. T2308 – Nisin

Overall ANOVA

Source	DF	Sum of Squares	Mean Square	F Value	Prob > F
Model	3	0.388382	0.129461	12.195052	9.273156e-05
Error	20	0.212317	0.010616	—	—
Total	23	0.600698	—	—	—

Tukey Test

Comparison	MeanDiff	SEM	q Value	Prob	Alpha	Sig	LCL	UCL
0.1 0	0.102602	0.059486	2.439248	0.337573	0.05	0	-0.063896	0.2691
0.2 0	0.230916	0.059486	5.489742	0.004726	0.05	1	0.064418	0.397414
0.2 0.1	0.128313	0.059486	3.050495	0.169688	0.05	0	-0.038185	0.294811
0.3 0	0.336144	0.059486	7.991423	8.614881e-05	0.05	1	0.169646	0.502642
0.3 0.1	0.233542	0.059486	5.552176	0.004277	0.05	1	0.067044	0.40004
0.3 0.2	0.105228	0.059486	2.501681	0.316651	0.05	0	-0.06127	0.271727

Tukey grouping (lowercase letters)

Group / formulation	Letter
0.3	a
0.2	ab
0.1	bc
0	c

4. F2332 – Nisin

Overall ANOVA

Source	DF	Sum of Squares	Mean Square	F Value	Prob > F
Model	3	2.193734	0.731245	48.041984	2.519086e-09
Error	20	0.304419	0.015221	—	—
Total	23	2.498153	—	—	—

Tukey Test

Comparison	MeanDiff	SEM	q Value	Prob	Alpha	Sig	LCL	UCL
0.1 0	-0.829021	0.07123	16.459631	0	0.05	1	-1.028388	-0.629654
0.2 0	-0.595816	0.07123	11.829522	3.173750e-07	0.05	1	-0.795183	-0.396449
0.2 0.1	0.233204	0.07123	4.630109	0.0183	0.05	1	0.033837	0.432571
0.3 0	-0.484614	0.07123	9.621675	7.169451e-06	0.05	1	-0.683981	-0.285247
0.3 0.1	0.344407	0.07123	6.837956	0.000539	0.05	1	0.14504	0.543774
0.3 0.2	0.111202	0.07123	2.207846	0.421976	0.05	0	-0.088165	0.310569

Tukey grouping (lowercase letters)

Group / formulation	Letter
0	a
0.3	b
0.2	b
0.1	c

5. T2308 – ZnO

Overall ANOVA

Source	DF	Sum of Squares	Mean Square	F Value	Prob > F
Model	3	0.370468	0.123489	9.87572	0.000934
Error	14	0.175061	0.012504	—	—
Total	17	0.545529	—	—	—

Tukey Test

Comparison	MeanDiff	SEM	q Value	Prob	Alpha	Sig	LCL	UCL
0.05 0	-0.304331	0.072181	5.962599	0.004234	0.05	1	-0.514128	-0.094533
0.07 0	-0.304331	0.072181	5.962599	0.004234	0.05	1	-0.514128	-0.094533
0.07 0.05	0	0.079071	0	1	0.05	0	-0.229822	0.229822
0.1 0	-0.304331	0.072181	5.962599	0.004234	0.05	1	-0.514128	-0.094533
0.1 0.05	0	0.079071	0	1	0.05	0	-0.229822	0.229822
0.1 0.07	0	0.079071	0	1	0.05	0	-0.229822	0.229822

Tukey grouping (lowercase letters)

Group / formulation	Letter
0	a
0.05	b
0.07	b
0.1	b

6. F2332 – ZnO

Overall ANOVA

Source	DF	Sum of Squares	Mean Square	F Value	Prob > F
Model	3	5.45357	1.817857	521.877635	1.343370e-14
Error	14	0.048766	0.003483	—	—
Total	17	5.502336	—	—	—

Tukey Test

Comparison	MeanDiff	SEM	q Value	Prob	Alpha	Sig	LCL	UCL
0.05 0	-1.154425	0.038097	42.853957	0	0.05	1	-1.265155	-1.043695
0.07 0	-1.123988	0.038097	41.724073	0	0.05	1	-1.234718	-1.013258
0.07 0.05	0.030437	0.041733	1.031438	0.883728	0.05	0	-0.090861	0.151736
0.1 0	-1.21853	0.038097	45.233626	0	0.05	1	-1.32926	-1.1078
0.1 0.05	-0.064105	0.041733	2.172331	0.443625	0.05	0	-0.185404	0.057194
0.1 0.07	-0.094542	0.041733	3.203769	0.153737	0.05	0	-0.215841	0.026756

Tukey grouping (lowercase letters)

Group / formulation	Letter
0	a
0.07	b
0.05	b
0.1	b

7. T2308 – CL / TT / EL / LG (5%)

Overall ANOVA

Source	DF	Sum of Squares	Mean Square	F Value	Prob > F
Model	3	0.002159	0.00072	0.070506	0.975171
Error	25	0.255136	0.010205	—	—
Total	28	0.257295	—	—	—

Tukey Test

Comparison	MeanDiff	SEM	q Value	Prob	Alpha	Sig	LCL	UCL
CL(5%) 0%	0.018929	0.061172	0.437621	0.989471	0.05	0	-0.149333	0.187191
TT 5% 0%	0.007813	0.058325	0.189437	0.999118	0.05	0	-0.152619	0.168244
TT 5% CL(5%)	-0.011117	0.061172	0.257	0.997811	0.05	0	-0.179378	0.157145
EL 5% 0%	-0.004621	0.050511	0.129382	0.999718	0.05	0	-0.143559	0.134317
EL 5% CL(5%)	-0.02355	0.053773	0.619368	0.97131	0.05	0	-0.171461	0.12436
EL 5% TT 5%	-0.012434	0.050511	0.348125	0.994622	0.05	0	-0.151372	0.126504

Tukey grouping (lowercase letters)

Group / formulation	Letter
CL(5%)	a
TT 5%	a
0%	a
EL 5%	a

8. F2332 – CL / TT / EL / LG (5%)

Overall ANOVA

Source	DF	Sum of Squares	Mean Square	F Value	Prob > F
Model	4	4.965066	1.241267	378.905855	0
Error	19	0.062243	0.003276	—	—
Total	23	5.027309	—	—	—

Tukey Test

Comparison	MeanDiff	SEM	q Value	Prob	Alpha	Sig	LCL	UCL
CL 5% F2332	-0.753283	0.034658	30.737635	0	0.05	1	-0.857506	-0.649059
TT 5% F2332	-0.996907	0.036945	38.160004	0	0.05	1	-1.10801	-0.885804
TT 5% CL 5%	-0.243624	0.038395	8.973511	3.857619e-05	0.05	1	-0.359086	-0.128163
EL 5% F2332	-1.056081	0.034658	43.093275	0	0.05	1	-1.160304	-0.951857
EL 5% CL 5%	-0.302798	0.036199	11.829624	7.296075e-07	0.05	1	-0.411656	-0.19394
EL 5% TT 5%	-0.059174	0.038395	2.179566	0.550026	0.05	0	-0.174635	0.056288
LG 5% F2332	-1.21853	0.036945	46.643377	0	0.05	1	-1.329633	-1.107427
LG 5% CL 5%	-0.465247	0.038395	17.136641	0	0.05	1	-0.580709	-0.349786
LG 5% TT 5%	-0.221623	0.040472	7.744225	0.000241	0.05	1	-0.34333	-0.099916
LG 5% EL 5%	-0.162449	0.038395	5.983564	0.003658	0.05	1	-0.277911	-0.046988

Tukey grouping (lowercase letters)

Group / formulation	Letter
F2332	a
CL 5%	b
TT 5%	c
EL 5%	c
LG 5%	d

S2.3 CA data

1. T2308 – Curcumin

Overall ANOVA

Source	DF	Sum of Squares	Mean Square	F Value	Prob > F
Model	3	1034.246393	344.748798	49.196797	3.632421e-10
Error	23	161.17355	7.007546	—	—
Total	26	1195.419942	—	—	—

Tukey Test

Comparison	MeanDiff	SEM	q Value	Prob	Alpha	Sig	LCL	UCL
0.05 0	-11.909125	1.621058	10.389538	1.020902e-06	0.05	1	-16.395087	-7.423163
0.1 0	-13.633232	1.370044	14.072759	6.283892e-07	0.05	1	-17.424562	-9.841902
0.1 0.05	-1.724107	1.659206	1.469532	0.728648	0.05	0	-6.315636	2.867421
0.15 0	-14.102438	1.323588	15.068021	1.039195e-05	0.05	1	-17.76521	-10.439665
0.15 0.05	-2.193313	1.621058	1.913449	0.540108	0.05	0	-6.679275	2.29265
0.15 0.1	-0.469205	1.370044	0.484332	0.985825	0.05	0	-4.260535	3.322125

Tukey grouping (lowercase letters)

Level	Tukey group
0%	a
0.05	b
0.10	b
0.15	b

2. F2332 – Curcumin

Overall ANOVA

Source	DF	Sum of Squares	Mean Square	F Value	Prob > F
Model	3	1620.837117	540.279039	23.538176	2.502277e-07
Error	24	550.879425	22.953309	—	—
Total	27	2171.716542	—	—	—

Tukey Test

Comparison	MeanDiff	SEM	q Value	Prob	Alpha	Sig	LCL	UCL
0.05 0	-10.324794	2.414418	6.047613	0.001399	0.05	1	-16.985227	-3.66436
0.1 0	-12.817371	2.8053	6.46152	0.000674	0.05	1	-20.556096	-5.078647
0.1 0.05	-2.492578	2.67227	1.319117	0.787702	0.05	0	-9.864327	4.879171
0.15 0	-21.373643	2.560876	11.80334	1.503024e-07	0.05	1	-28.438099	-14.309186
0.15 0.05	-11.048849	2.414418	6.471719	0.000662	0.05	1	-17.709283	-4.388416
0.15 0.1	-8.556271	2.8053	4.313406	0.02639	0.05	1	-16.294996	-0.817547

Tukey grouping (lowercase letters)

Level	Tukey group
0%	a
0.05	b
0.10	b
0.15	c

3. T2308 – Nisin

Overall ANOVA

Source	DF	Sum of Squares	Mean Square	F Value	Prob > F
Model	3	824.023524	274.674508	57.455143	7.555512e-12
Error	27	129.078291	4.780677	—	—
Total	30	953.101815	—	—	—

Tukey Test

Comparison	MeanDiff	SEM	q Value	Prob	Alpha	Sig	LCL	UCL
0.1 0	-9.670319	1.062436	12.872203	9.060525e-08	0.05	1	-12.577744	-6.762895
0.2 0	-10.567188	1.093238	13.669722	9.076994e-08	0.05	1	-13.558903	-7.575472
0.2 0.1	-0.896868	1.062436	1.193825	0.832934	0.05	0	-3.804292	2.010556
0.3 0	-14.284458	1.180832	17.107662	0	0.05	1	-17.51588	-11.053037
0.3 0.1	-4.614139	1.152374	5.662552	0.002333	0.05	1	-7.767683	-1.460594
0.3 0.2	-3.717271	1.180832	4.451958	0.019587	0.05	1	-6.948692	-0.485849

Tukey grouping (lowercase letters)

Level	Tukey group
0%	a
0.10	b
0.20	b
0.30	c

4. F2332 – Nisin

Overall ANOVA

Source	DF	Sum of Squares	Mean Square	F Value	Prob > F
Model	3	1397.86018	465.953393	22.506717	1.277386e-07
Error	28	579.680064	20.702859	—	—
Total	31	1977.540244	—	—	—

Tukey Test

Comparison	MeanDiff	SEM	q Value	Prob	Alpha	Sig	LCL	UCL
0.1 0	-8.553516	2.293004	5.275393	0.004516	0.05	1	-14.814133	-2.292899
0.2 0	-12.373071	2.354869	7.430632	7.800150e-05	0.05	1	-18.802599	-5.943544
0.2 0.1	-3.819556	2.210922	2.443174	0.328897	0.05	0	-9.856062	2.216951
0.3 0	-18.913696	2.354869	11.358595	3.548737e-08	0.05	1	-25.343224	-12.484169
0.3 0.1	-10.360181	2.210922	6.626878	0.000362	0.05	1	-16.396687	-4.323674
0.3 0.2	-6.540625	2.27502	4.065829	0.036184	0.05	1	-12.752139	-0.329111

Tukey grouping (lowercase letters)

Level	Tukey group
0%	a
0.10	b
0.20	b
0.30	c

5. T2308 – ZnO

Overall ANOVA

Source	DF	Sum of Squares	Mean Square	F Value	Prob > F
Model	3	733.441065	244.480355	17.861121	5.425256e-07
Error	32	438.011223	13.687851	—	—
Total	35	1171.452288	—	—	—

Tukey Test

Comparison	MeanDiff	SEM	q Value	Prob	Alpha	Sig	LCL	UCL
0.05 0	-7.049775	1.754926	5.681086	0.001807	0.05	1	-11.804506	-2.295044
0.07 0	-10.830475	1.754926	8.727776	3.804084e-06	0.05	1	-15.585206	-6.075744
0.07 0.05	-3.7807	1.65456	3.231503	0.122868	0.05	0	-8.263504	0.702104
0.1 0	-12.12925	1.849855	9.272809	1.242310e-06	0.05	1	-17.141177	-7.117323
0.1 0.05	-5.079475	1.754926	4.093313	0.032655	0.05	1	-9.834206	-0.324744
0.1 0.07	-1.298775	1.754926	1.046622	0.880164	0.05	0	-6.053506	3.455956

Tukey grouping (lowercase letters)

Level	Tukey group
0%	a
0.05	b
0.07	bc
0.10	c

6. F2332 – ZnO

Overall ANOVA

Source	DF	Sum of Squares	Mean Square	F Value	Prob > F
Model	3	450.117067	150.039022	5.401941	0.004822
Error	27	749.925623	27.775023	—	—
Total	30	1200.042689	—	—	—

Tukey Test

Comparison	MeanDiff	SEM	q Value	Prob	Alpha	Sig	LCL	UCL
0.05 0	-0.492571	2.655932	0.262281	0.997679	0.05	0	-7.760697	6.775554
0.07 0	-5.395155	2.932069	2.602224	0.277293	0.05	0	-13.418948	2.628639
0.07 0.05	-4.902583	2.77764	2.496112	0.311552	0.05	0	-12.503771	2.698604
0.1 0	-8.913571	2.655932	4.746241	0.011892	0.05	1	-16.181697	-1.645446
0.1 0.05	-8.421	2.484397	4.793555	0.010961	0.05	1	-15.219709	-1.622291
0.1 0.07	-3.518417	2.77764	1.791374	0.591252	0.05	0	-11.119604	4.082771

Tukey grouping (lowercase letters)

Level	Tukey group
0%	a
0.05	a
0.07	ab
0.10	b

7. T2308 – CL / TT / EL / LG (5%)

Overall ANOVA

Source	DF	Sum of Squares	Mean Square	F Value	Prob > F
Model	4	902.546054	225.636514	30.487127	3.558265e-12
Error	44	325.645853	7.401042	—	—
Total	48	1228.191907	—	—	—

Tukey Test

Comparison	MeanDiff	SEM	q Value	Prob	Alpha	Sig	LCL	UCL
CL 5% T2308	-12.819575	1.29044	14.049178	0	0.05	1	-16.489723	-9.149427
TT 5% T2308	-11.76783	1.264101	13.165261	0	0.05	1	-15.363069	-8.17259
TT 5% CL 5%	1.051745	1.188666	1.251313	0.900987	0.05	0	-2.328947	4.432438
EL 5% T2308	-8.404148	1.264101	9.402141	3.610409e-07	0.05	1	-11.999387	-4.808908
EL 5% CL 5%	4.415427	1.188666	5.253249	0.004926	0.05	1	1.034735	7.79612
EL 5% TT 5%	3.363682	1.160019	4.100764	0.043718	0.05	1	0.064464	6.6629

LG 5% T2308	-6.985319	1.321918	7.473028	3.575363e-05	0.05	1	-10.744997	-3.225642
LG 5% CL 5%	5.834256	1.249977	6.600829	0.000267	0.05	1	2.279188	9.389323
LG 5% TT 5%	4.78251	1.222767	5.531298	0.002769	0.05	1	1.304829	8.260191
LG 5% EL 5%	1.418828	1.222767	1.640971	0.773464	0.05	0	-2.058853	4.896509

Tukey grouping (lowercase letters)

Level	Tukey group
T2308	a
CL 5%	c
TT 5%	c
EL 5%	b
LG 5%	b

8. F2332 – CL / TT / EL / LG (5%)

Overall ANOVA

Source	DF	Sum of Squares	Mean Square	F Value	Prob > F
Model	4	1925.610614	481.402654	39.122588	4.152234e-14
Error	45	553.724089	12.30498	—	—
Total	49	2479.334703	—	—	—

Tukey Test

Comparison	MeanDiff	SEM	q Value	Prob	Alpha	Sig	LCL	UCL
CL 5% F2332	-16.500225	1.644503	14.189599	0	0.05	1	-21.173002	-11.827448
TT 5% F2332	-6.672134	1.815483	5.197418	0.005428	0.05	1	-11.83074	-1.513528
TT 5% CL 5%	9.828091	1.57628	8.817607	1.360292e-06	0.05	1	5.349167	14.307016
EL 5% F2332	-17.353705	1.605673	15.284459	0	0.05	1	-21.916148	-12.791262
EL 5% CL 5%	-0.853479	1.329236	0.908042	0.967238	0.05	0	-4.63044	2.923481
EL 5% TT 5%	-10.681571	1.535726	9.836404	1.086291e-07	0.05	1	-15.045263	-6.317879
LG 5% F2332	-12.640071	1.875022	9.533626	2.356428e-07	0.05	1	-17.967857	-7.312286
LG 5% CL 5%	3.860154	1.644503	3.319593	0.149143	0.05	0	-0.812623	8.532931
LG 5% TT 5%	-5.967937	1.815483	4.648868	0.016029	0.05	1	-11.126544	-0.809331
LG 5% EL 5%	4.713633	1.605673	4.151582	0.039748	0.05	1	0.15119	9.276076

Tukey grouping (lowercase letters)

Level	Tukey group
F2332	a
CL 5%	cd
TT 5%	b
EL 5%	d
LG 5%	c

S2.4 DMA data

Sample	Young's Modulus
	MPa
T2308	983.4 ± 157.9 a
T2308/curcumin(5%)	551 ± 43.1 b
T2308/curcumin(10%)	458.1 ± 109.1 b
T2308/curcumin(15%)	461.5 ± 87.4 b
F2332	100 ± 5.0 a
F2332/curcumin(5%)	77.9 ± 18.9 a
F2332/curcumin(10%)	87.5 ± 29.3 a
F2332/curcumin(15%)	82.1 ± 6.4 a

Sample	Young's Modulus
	MPa
T2308	983.4 ± 157.9 a
T2308/nisin(10%)	932 ± 80.5 a
T2308/nisin(20%)	933.9 ± 32.5 a
T2308/nisin(30%)	878.5 ± 30.8 a
F2332	100 ± 5.0 a
F2332/nisin(10%)	120.9 ± 8.0 a
F2332/nisin(20%)	260.1 ± 8.2 b
F2332/nisin(30%)	233.8 ± 19.0 b

1. T2308 – Curcumin

Overall ANOVA

Source	DF	Sum of Squares	Mean Square	F Value	Prob > F
Model	3	5.639259e+05	1.879753e+05	16.262825	0.000913
Error	8	9.246870e+04	1.155859e+04	—	—
Total	11	6.563946e+05	—	—	—

Tukey Test

Comparison	MeanDiff	SEM	q Value	Prob	Alpha	Sig	LCL	UCL
0.05 0	-432.4	87.782259	6.966168	0.005064	0.05	1	-713.509654	-151.290346
0.1 0	-525.3	87.782259	8.462831	0.00148	0.05	1	-806.409654	-244.190346
0.1 0.05	-92.9	87.782259	1.496663	0.722382	0.05	0	-374.009654	188.209654
0.15 0	-521.866667	87.782259	8.407518	0.001545	0.05	1	-802.976321	-240.757012
0.15 0.05	-89.466667	87.782259	1.44135	0.74358	0.05	0	-370.576321	191.642988
0.15 0.1	3.433333	87.782259	0.055313	0.999976	0.05	0	-277.676321	284.542988

Tukey grouping (lowercase letters)

Level	Tukey group
0%	a
0.05	b
0.10	b
0.15	b

2. F2332 – Curcumin

Overall ANOVA

Source	DF	Sum of Squares	Mean Square	F Value	Prob > F
Model	3	983.396669	327.79889	1.140228	0.384137
Error	9	2587.369008	287.485445	—	—
Total	12	3570.765677	—	—	—

Tukey Test

Comparison	MeanDiff	SEM	q Value	Prob	Alpha	Sig	LCL	UCL
0.05 0	-22.049167	12.949897	2.407913	0.376086	0.05	0	-62.476105	18.377771
0.1 0	-12.4525	12.949897	1.359895	0.773717	0.05	0	-52.879438	27.974438
0.1 0.05	9.596667	13.844023	0.980332	0.897151	0.05	0	-33.621548	52.814881
0.15 0	-17.885833	12.949897	1.95325	0.539925	0.05	0	-58.312771	22.541105
0.15 0.05	4.163333	13.844023	0.425299	0.989905	0.05	0	-39.054881	47.381548
0.15 0.1	-5.433333	13.844023	0.555033	0.978247	0.05	0	-48.651548	37.784881

Tukey grouping (lowercase letters)

Level	Tukey group
0%	a
0.05	a
0.10	a
0.15	a

3. T2308 – Nisin

Overall ANOVA

Source	DF	Sum of Squares	Mean Square	F Value	Prob > F
Model	3	1.652382e+04	5507.940833	0.661051	0.598764
Error	8	6.665676e+04	8332.095	—	—
Total	11	8.318058e+04	—	—	—

Tukey Test

Comparison	MeanDiff	SEM	q Value	Prob	Alpha	Sig	LCL	UCL
0.1 0	-51.4	74.530061	0.975319	0.89822	0.05	0	-290.071457	187.271457
0.2 0	-49.466667	74.530061	0.938634	0.907779	0.05	0	-288.138124	189.204791
0.2 0.1	1.933333	74.530061	0.036685	0.999993	0.05	0	-236.738124	240.604791
0.3 0	-104.9	74.530061	1.990485	0.528857	0.05	0	-343.571457	133.771457
0.3 0.1	-53.5	74.530061	1.015167	0.887319	0.05	0	-292.171457	185.171457
0.3 0.2	-55.433333	74.530061	1.051852	0.876822	0.05	0	-294.104791	183.238124

Tukey grouping (lowercase letters)

Level	Tukey group
0%	a
0.10	a
0.20	a
0.30	a

4. F2332 – Nisin

Overall ANOVA

Source	DF	Sum of Squares	Mean Square	F Value	Prob > F
Model	3	6.332911e+04	2.110970e+04	178.866014	2.443387e-08
Error	9	1062.176808	118.019645	—	—
Total	12	6.439129e+04	—	—	—

Tukey Test

Comparison	MeanDiff	SEM	q Value	Prob	Alpha	Sig	LCL	UCL
0.1 0	20.9475	8.297276	3.570357	0.122067	0.05	0	-4.954904	46.849904
0.2 0	160.114167	8.297276	27.290356	0	0.05	1	134.211763	186.016571
0.2 0.1	139.166667	8.870161	22.188028	2.493393e-07	0.05	1	111.475832	166.857501
0.3 0	133.880833	8.297276	22.819066	1.700142e-07	0.05	1	107.978429	159.783237
0.3 0.1	112.933333	8.870161	18.005518	2.142339e-06	0.05	1	85.242499	140.624168
0.3 0.2	-26.233333	8.870161	4.18251	0.064026	0.05	0	-53.924168	1.457501

Tukey grouping (lowercase letters)

Level	Tukey group
0%	a
0.10	a
0.20	b
0.30	b
